# Synthesis of Substituted Tetralins via Nitrogen Deletion/Diels–Alder Cascade Reaction

**DOI:** 10.1021/acs.joc.4c01959

**Published:** 2024-11-22

**Authors:** Zixuan Zang, Wen Ye, Kehang Cheng, Xiaotai Wang, Xiaodong Jin

**Affiliations:** Department of Chemistry, Xi’an Jiaotong-Liverpool University, Suzhou 215123, China

## Abstract

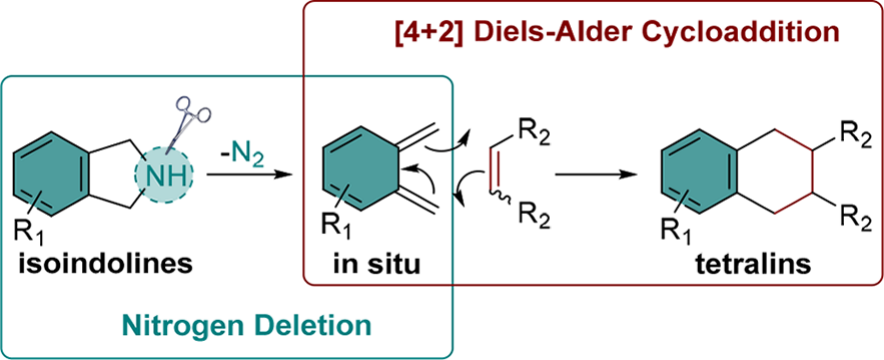

Skeletal editing is an important approach for the modification and diversification of biologically active molecules. The utilization of nitrogen deletion strategies in skeletal editing has recently emerged as a new method for compound modification. Here, we report an unexpected nitrogen deletion in isoindolines. Contrary to the anticipated outcome of cyclobutane formation via intramolecular radical couplings, the nitrogen deletion of isoindoline triggers a Diels–Alder cycloaddition facilitated by the in situ formation of *ortho*-xylylene to yield tetraline. Inspired by this reaction, we developed a new strategy for synthesizing substituted tetralins, employing isoindoline, nitrogen deletion reagent (anomeric amide), and dienophiles. This methodology demonstrates a new pathway for tetralin synthesis and the modification and diversification of isoindolines.

## Introduction

**Scheme 1:**
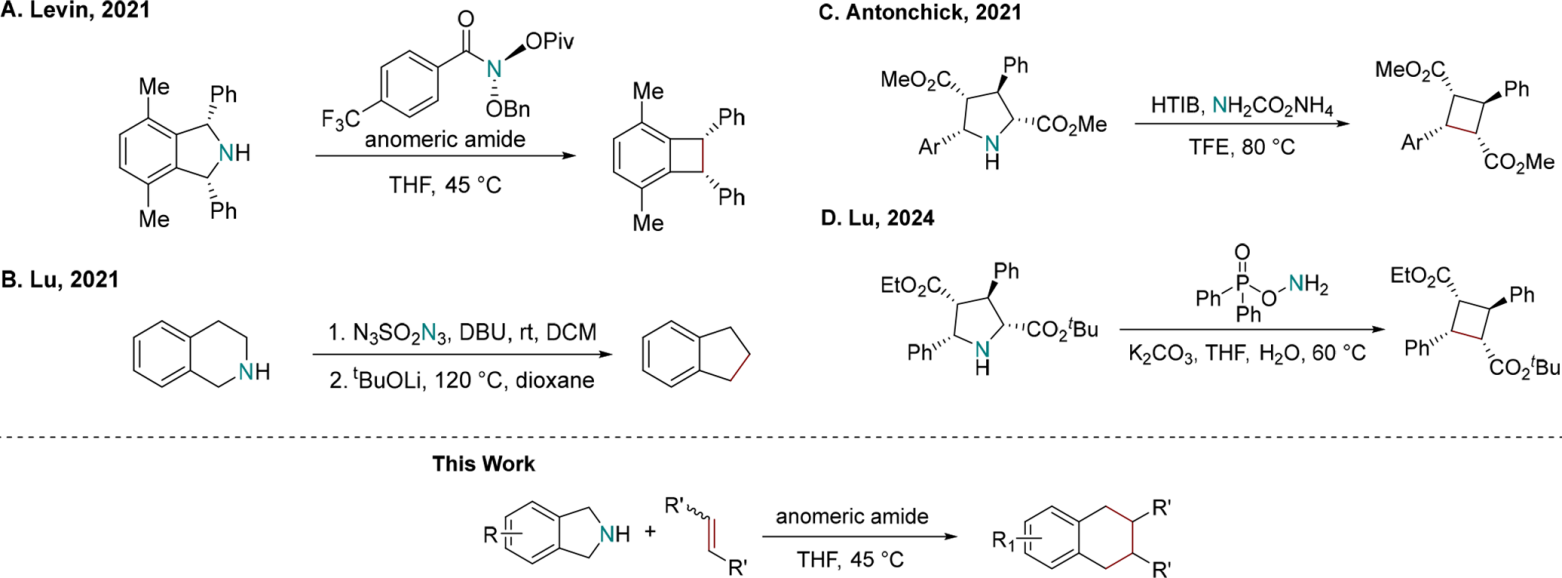
Examples of Skeletal Editing Strategy through Nitrogen Deletion and This Work

The utilization of skeletal editing strategies through nitrogen deletion has attracted significant attention in the synthesis of cycloalkanes by contracting N-heterocycles.^1−6^ In particular, the contraction is realized by the single atom deletion of nitrogen in an N-heterocycle followed by intramolecular new C–C bond formation. Several nitrogen deletion reagents, including anomeric amide,^7^ sulfuryl diazide,^8,9^ iodonitrene,^10^ and *O*-diphenylphosphinylhydroxylamine (DPPH)^11^ have been used and effectively enable the ring contractions by activation of secondary amines in heterocycles (Scheme 1).

Up to date, the nitrogen deletion strategy has shown promising results in synthesizing cyclobutanes,^7,9−12^ bicyclo[1.1.1]pentanes (BCPs),^13^ and polycyclic aromatics.^14,15^ Additionally, anomeric amide has demonstrated good yields in direct deaminative functionalizations.^16,17^ Despite the different deletion reagents, the generation of diradicals after nitrogen extrusion is generally accepted. Mechanistic studies investigating the nitrogen deletion strategy using anomeric amide have suggested the formation of isodiazene intermediates during the reaction.^7,18^ Diradicals are then formed after nitrogen extrusion, and these diradicals undergo intramolecular radical couplings, resulting in the formation of new carbon–carbon bonds.^7,18^ To enhance the reactivity, conjugated radicals are preferred, requiring at least two benzylic substitutions in cyclic systems.^7^

Our interest lies in isoindolines, wherein the secondary amine could potentially generate diradicals stabilized by the same benzene ring. Instead of yielding cyclobutanes after nitrogen deletion, this reaction undergoes a Diels–Alder cycloaddition, giving a spiro dimer due to the in situ formation of *ortho*-xylylene. Encouraged by this unexpected outcome, we introduced dienophiles into the reaction mixture, leading to the successful production of tetralins as the final product. Given the significance of isoindoline^19−24^ and tetralin skeletons^25−32^ in synthesizing biologically active molecules, this method introduces a novel synthetic route to tetralin synthesis and holds potential for application in late-stage skeletal editing strategies in synthetic planning.

## Results and Discussion

### Nitrogen Deletion of Isoindoline

The nitrogen deletion reagent, anomeric amide (*N*-(benzyloxy)-*N*-(pivaloyloxy)-4-(trifluoromethyl)-benzamide), was synthesized following the published protocol.^7,33^ With the anomeric amide in hand, isoindoline (**1**) was selected as the starting material and reacted with anomeric amide in THF at 45 °C for 16 h under an atmosphere of nitrogen. The product after purification was characterized by NMR and GC–MS. In the presence of anomeric amides, the secondary amine within isoindoline undergoes activation through nucleophilic substitution and subsequent elimination, yielding an isodiazene intermediate (**2**).^7,17^

**Scheme 2:**
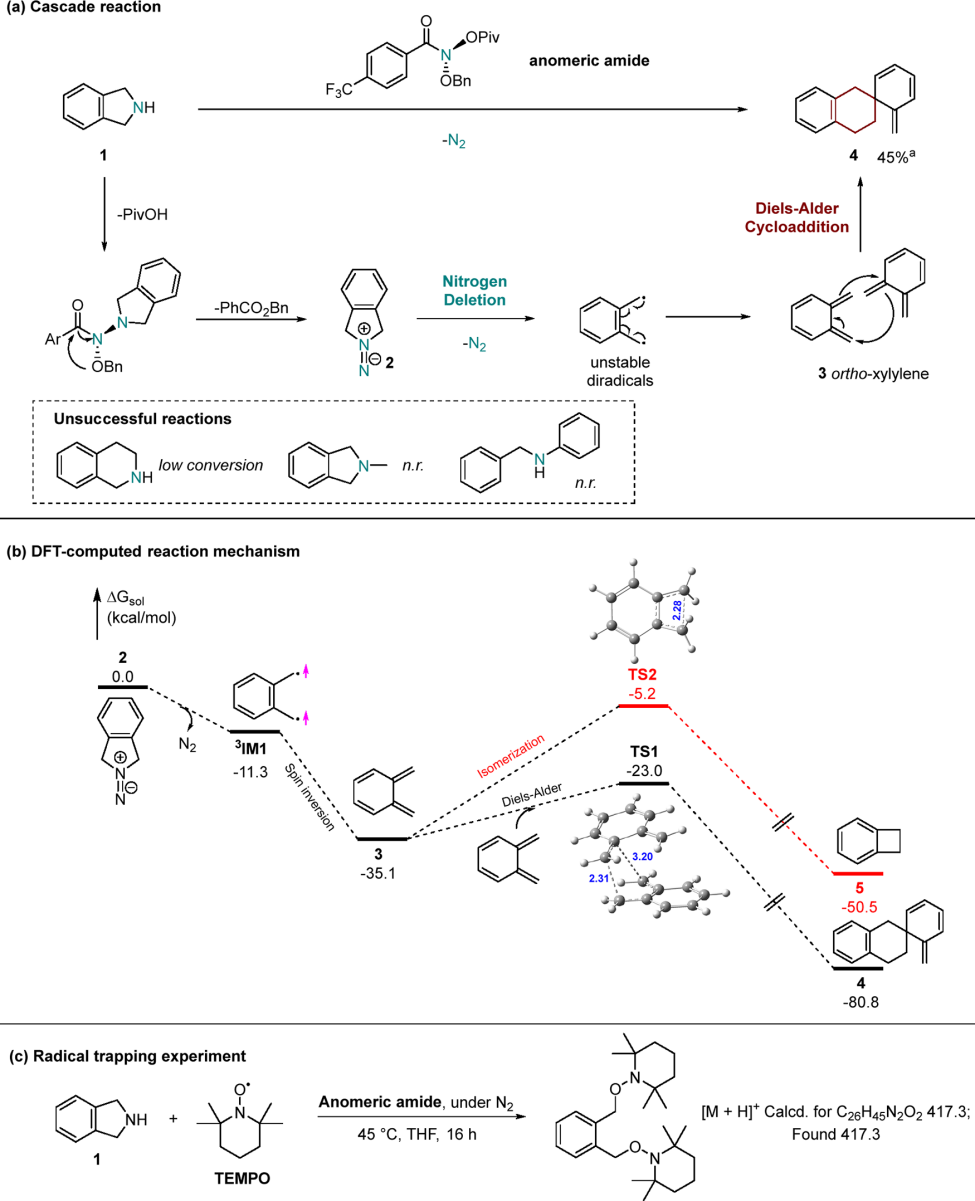
Proposed Mechanism for Nitrogen Deletion of Isoindoline Reaction conditions: (a) isoindoline (1 mmol) and anomeric amide (1.5 mmol) reacted in THF; (b) free energy profile computed with M06-2x^34^/6-311G(d,p)/SMD^35^ (THF) at 25 °C using the Gaussian 09^36^ program; (c) isoindoline (0.2 mmol), anomeric amide (0.3 mmol), and TEMPO (0.4 mmol) reacted in THF. These reactions were prepared in a glovebox and carried out in a sealed vial at 45 °C for 16 h.

The resulting diradical pair, stabilized by the benzene ring, is formed subsequent to nitrogen extrusion from the isodiazene.^7^ Due to the lack of additional phenyl groups, contrary to the anticipated diradical coupling reaction that typically leads to the formation of benzocyclobutene, a spiro dimer formed after deletion, strongly indicating the in situ generation of *ortho*-xylylene (**3**) during the reaction (Scheme 2). **3** then undergoes a dimerization, resulting in the production of the spiro compound **4** confirmed by NMR analysis.^37^ For unreactive amines, tetrahydroisoquinoline, which contains a single benzylic substitution, was mostly recovered after nitrogen deletion. A small amount of the rearrangement product hydrazine was detected,^7^ while indane was not observed, as confirmed by GC–MS analysis. As expected, no deletion product was found in *N*-methylated isoindolin or benzylaniline and the starting amines were recovered. This observation highlights the necessity for the substrate to possess a secondary amine and to exhibit less steric hindrance for successful nitrogen deletion to occur.

We have utilized density functional theory (DFT) calculations to establish a plausible mechanism that rationalizes the formation of the spiro dimer **4** (Scheme 2b). Extrusion of N_2_ from **2** is energetically downhill by 11.3 kcal/mol, forming the triplet diradical ^**3**^**IM1**, which then undergoes exergonic spin inversion to give the singlet *ortho*-xylylene (**3**). The chemoselectivity of **3** overwhelmingly favors Diels–Alder cycloaddition/dimerization via **TS1** to produce **4** over isomerization via **TS2** to generate benzocyclobutene (**5**), as the energy barrier **TS1** is lower than **TS2** by 17.8 kcal/mol and **4** is lower than **5** by 30.3 kcal/mol. Note that the calculated barrier Δ*G*^‡^ (**TS1**–**3** = 12.1 kcal/mol) and free energy Δ*G* (**4**–**3** = −45.7 kcal/mol) of the **3** → **4** reaction show a good level of consistency with the computational results of a recently reported study.^38^ The high stability of **4** can be attributed to the regaining of aromaticity in one of the six-membered rings.^39^ The radical trapping experiment using TEMPO was also conducted (Scheme 2c), and the radical capture product containing two TEMPO units was detected by LCMS. In summary, the computational results are in agreement with and support the experimental findings. This finding also aligns with the mechanism proposed for tetrahydroisoquinolines, where the diradicals underwent the dearomatizing spirocyclization.^18^ The in situ formation of compound **3** has also been reported in previous studies,^40^ such as using freshly prepared metallic nickel to induce the 1,4-debromination of α,α′-dibromo-*o*-xylene^37^ or extrusion of sulfur dioxide^41^ or nitrogen.^42^ This method presents a strategy for in situ *ortho*-xylylene preparation originating from a secondary amine and avoids using metals or thermally less stable starting materials. This method also yielded only the *trans* product, in contrast to the previous methods which typically produced a *trans*/*cis* mixture.^37^ Two steps deconstruction of 2-phenyl pyrrolidines to give conjugated and nonconjugated dienes have been observed via the thermal rearrangement (heated at 120 °C) of sulfamoyl azide intermediates.^43^

### Nitrogen Deletion/Diels–Alder Cascade Reaction

**Scheme 3:**
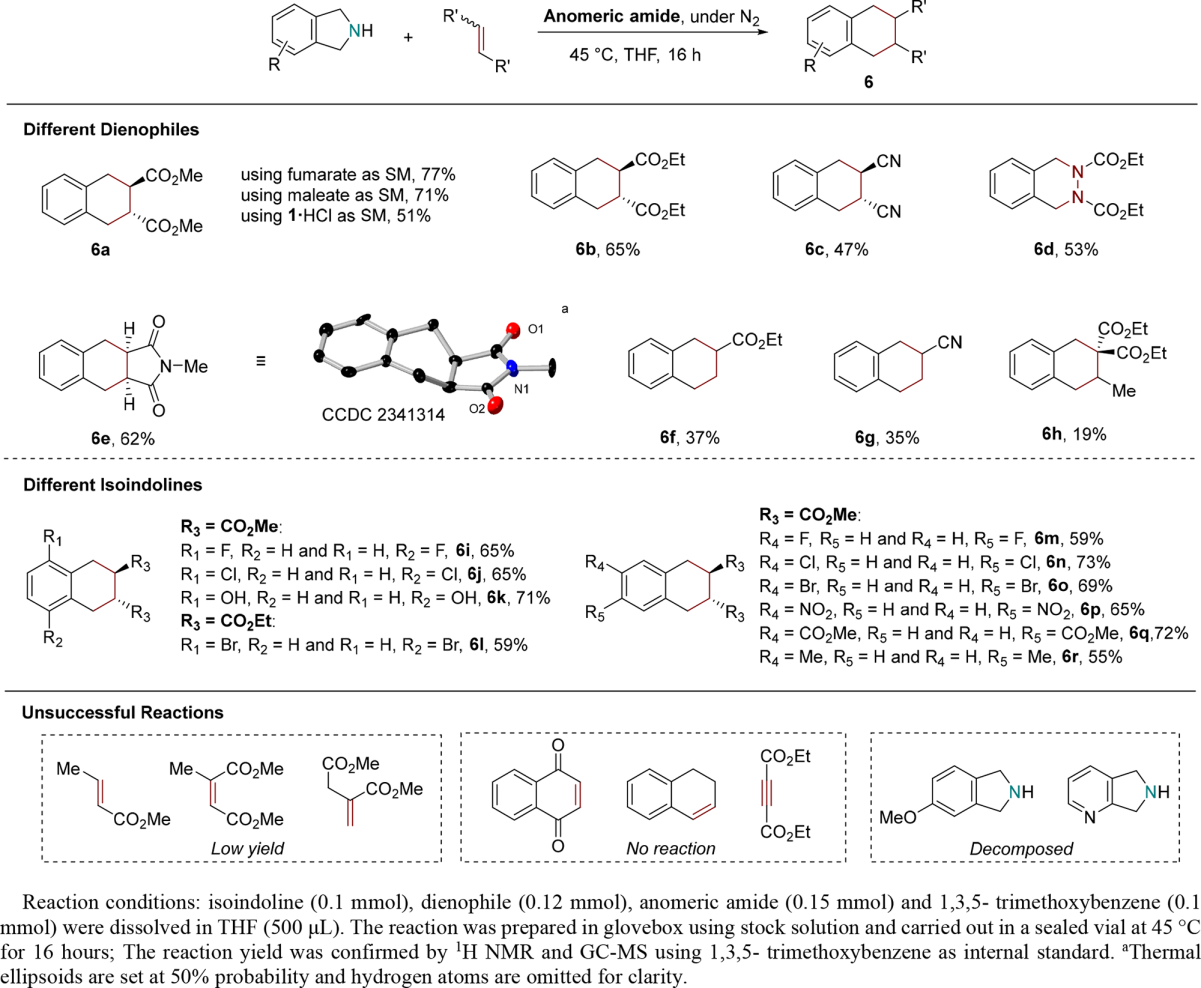
Nitrogen Deletion/Diels–Alder Cascade Reaction

The dimerization prompted our exploration into the potential of reacting in situ-formed *ortho*-xylylene with dienophiles via Diels–Alder reactions to yield tetralins (**6**). Various commonly used dienophiles were selected and screened in this study. The reactions were conducted under standard deletion conditions,^7^ employing air-free techniques to optimize yields. The yield was calculated using NMR and GC–MS with 1,3,5- trimethoxybenzene as an internal standard. Isolated yields were recorded by repeating the reactions in larger scale and are detailed in Experimental Section. The results of nitrogen deletion/Diels–Alder cascade reaction have been summarized in Scheme 3. Adding dimethyl fumarate to the reaction mixture successfully trapped diene **3** to yield only *trans* product **6a** (77%). A small quantity of **4** (<3%) was found, as confirmed by GC–MS. The stereochemistry in the dimethyl maleate was not reproduced in the product. Both *trans* and *cis* esters exclusively formed the *trans* product **6a** at 77% and 71% yield, respectively, as confirmed by ^1^H and ^13^C NMR analysis. The stereochemistry could be rationalized by the amine-catalyzed isomerization of dimethyl maleate.^44,45^ The isomerization of dimethyl maleate was confirmed by mixing it with isoindoline in THF, and *trans* alkene was formed, as product confirmed by ^1^H NMR (see Supporting Information). Given the commercial availability, cost, and storage conditions of amine and its hydrochloride salts, we also used isoindoline hydrochloride in the study. The reaction proceeded in the presence of triethylamine (2 equiv), while all other conditions remained unchanged. The yield of **6a** (51%) shows a significant decrease, may be due to poor solubility in THF, it is advisable to pretreat the amine salt with sodium hydroxide to acquire the free amine before the reaction. Halogen and nitro substituted isoindolines were brought as hydrochloride salts and used after pretreatment with sodium hydroxide. Diethyl fumarate also reacted and gave *trans* product **6b** despite the slight decrease in yield (65%). Fumaronitrile introduced nitrile groups into the tetralin structure, resulting in product **6c** in an acceptable yield (47%). When using the aza-dienophile, diethyl azodicarboxylate (DEAD), *N*-substituted tetrahydrophthalazine was formed as the product (**6d**, 53%) by reacting with *ortho*-xylylene. This opens the possibilities for benzofused heterocycle synthesis starting from isoindoline. The addition of *N*-methylmaleimides successfully introduced succinimide into the tetralin skeleton and gave a moderate yield (**6e**, 62%). The structure of **6e** was also confirmed by single-crystal X-ray diffraction (CCDC no. 2341314).

Monosubstituted alkenes ethyl acrylate and acrylonitrile, bearing one electron-withdrawing group, reacted but resulted in lower yields (**6f**, 37%; **6g**, 35%), as confirmed using NMR analysis and chiral supercritical fluid chromatography mass spectrometry (SFC-MS) column. Other unsymmetric dienophiles such as diethyl ethylidenemalonate, methyl crotonate, dimethyl itaconate, and dimethyl citraconate gave poor yields (**6h**, 19%, others <10%) and produced **4** as the major product. Less electron-deficient dienophiles such as 1,4-naphthoquinone, 1,2-dihydronaphthalene, and diethyl acetylenedicarboxylate did not produce the desired products and also gave **4** as a major product. The competition with dimerization requires the dienophiles to be more reactive to form targeted tetralins as the major product.

As dimethyl fumarate has been proven to be the most reactive dienophile, it was selected as the starting dienophile in screening substituted amines. Halide-containing isoindolines all gave moderate yields (**6i**, **6j**, **6l**–**6o**, yield ≥59%). Two *trans* Diels–Alder products were obtained, as confirmed by NMR analysis and SFC-MS. Similar yields were obtained despite the different substitution position (C4 or C5) and halide groups (F, Cl, or Br) on isoindolines. The successful synthesis of **6l** and **6o** provides cross coupling sites for further functionalization on the aromatic rings. The 5-nitro and carboxylic acid methyl ester substitution also gave a moderate yield (**6o**, 65%; **6q**, 72%), demonstrating the tolerance of electron withdrawing groups on the benzene ring. The weak activating methyl group on C5 shows a slight decrease in yield, giving **6r** as the product (55%). The hydroxy group on C4 gave a better yield (**6k**, 55%). Electron rich 5-methoxyisoindoline, which contains an activating methoxy group was decomposed after reaction, and no starting amine or product was detected. We suspect that 5-methoxyisoindoline underwent oxidation caused by the anomeric amide and decomposed after reaction. The observation is in agreement with literature.^7^ The pyridine ring also underwent unspecific decomposition after the reaction, as no desired products or dimerization were observed.

## Conclusions

In conclusion, this study explores the efficacy of nitrogen deletion on isoindolines using an anomeric amide. The nitrogen deletion of isoindoline triggers a Diels–Alder reaction, yielding a tetralin-motif-containing compound instead of cyclobutanes. The observation suggests the in situ formation of *ortho*-xylylene during the reaction. Mechanistic investigations involving the DFT calculation and TEMPO experiments provide additional insight into the formation of *ortho*-xylylene and the absence of radical coupling products. The addition of dienophiles in the reaction demonstrates a nitrogen deletion/Diels–Alder cascade reaction with good functional group tolerance on both dienophiles and isoindolines. The results show a preference for electron-deficient dienophiles and isoindolines. Overall, we reported a nitrogen deletion/Diels–Alder methodology that opens a new synthetic protocol for substituted tetralin synthesis, which could be utilized in the synthesis and modification of biologically active compounds.

## Experimental Section

### General Information

All of the reactions were performed in oven-dried glassware under nitrogen. Anhydrous acetonitrile (MeCN), dichloromethane (DCM), and tetrahydrofuran (THF) were obtained from a solvent tower using the PureSolv solvent purification system, degassed under an atmosphere of nitrogen. Unless noted otherwise, all chemicals were purchased from Sigma-Aldrich or Adamas Reagent and used as received without further purification. Anomeric amide was stored in a freezer at −20 °C. **Caution!** The reaction proceeded rapidly, generating visible quantities of gas. Ames II testing showed mutagenic activity in bacteria, though at levels comparable to other routinely handled reagents like benzyl chloride.^17^ Further investigation into this reactivity can be found in literature.^17^ Analytical thin-layer chromatography (TLC) was carried out using silica gel plates (Merck silica gel 60, F254) and visualized by UV light or stain. Automatic chromatography was conducted using a Biotage Isolera Four Flash Chromatography System, and the columns used were Biotage Rening prepacked cartridges (40–63 μm, 10 or 30 g). Nuclear magnetic resonance spectra for ^1^H NMR, ^19^F{^1^H} NMR, ^13^C{^1^H} NMR, and DEPT NMR spectra were recorded with a Bruker AVANCE III 400 spectrometer operating at 400 MHz for ^1^H NMR. The chemical shifts will be recorded in parts per million (ppm, δ) and are referenced to solvent CDCl_3_ (7.26 ppm, ^1^H and 77.160 ppm, ^13^C). ^1^H NMR will be reported as follows: ^1^H NMR (400 MHz, solvent) δ chemical shift (shape of the peak, coupling constant (Hz), and number of H atoms). Mass spectra were recorded on an Agilent 8860/5977C Series GC/MSD System and 1260 Infinity II/InfinityLab LC/MSD series. Supercritical fluid chromatography data were collected using Shimadzu Nexera UC/SFC-30A with an SPD-M20A detector. High-resolution mass spectrometry (HRMS) data were recorded on Thermo Fisher Scientific Orbitrap Exploris 120.

### General Procedure A for the Cascade Reaction

The experiment was prepared in a nitrogen-filled glovebox, and the sealed vial was heated on a hot plate in a fume hood. In a 10 mL screw cap vial, anomeric amide (593 mg, 1.5 mmol, 1.5 equiv) and dienophile (1.2 mmol, 1.2 equiv) were dissolved in degassed anhydrous THF (3 mL), then isoindoline solution (119 mg, 1 mmol, 1 equiv) dissolved in 2 mL THF was added dropwise. After stirring for 20 h at 45 °C in an oil bath, the reaction mixture cooled to room temperature and was diluted with 8 mL of diethyl ether. The solution was washed with a saturated sodium carbonate solution and brine. The organic extracts were dried over sodium sulfate. Solvent was removed under vacuum, and the product was purified by flash chromatography.

### General Procedure B for the Cascade Reaction

The experiment was prepared in a nitrogen-filled glovebox, and the sealed vial was heated on a hot plate in a fume hood. In a 4 mL screw cap vial, anomeric amide (297 mg, 0.75 mmol, 1.5 equiv) and dimethyl fumarate (86 mg, 0.6 mmol, 1.2 equiv) were dissolved in degassed anhydrous THF (1.5 mL), and then isoindoline solution (0.5 mmol, 1 equiv) dissolved in 1 mL of THF was added dropwise. After stirring for 20 h at 45 °C in an oil bath, the reaction mixture cooled to room temperature and was diluted with 8 mL of diethyl ether. The solution was washed with saturated sodium carbonate solution and brine. The organic extracts were dried over sodium sulfate. Solvent was removed under vacuum, and the product was purified by flash chromatography.

#### 6-Methylene-3′,4′-dihydro-1′*H*-spiro[cyclohexane-1,2′-naphthalene]-2,4-diene (**4**)^46^

**4** was prepared according to general procedure A without the addition of dienophile. A colorless oil (80 mg, 40% yield) was collected after purification by auto column chromatography (0–20% ethyl acetate in hexane). ^1^H NMR (400 MHz, CDCl_3_) δ 7.18–7.11 (m, 3H), 7.09 (m, 1H), 6.19 (d, *J* = 9.3 Hz, 1H), 5.94 (m, 1H), 5.90–5.85 (m, 1H), 5.83 (d, *J* = 9.0 Hz, 1H), 5.09 (s, 1H), 5.01 (s, 1H), 3.01–2.80 (m, 4H), 1.96 (m, 1H), 1.75 (m, 1H). ^13^C{^1^H} NMR (101 MHz, CDCl_3_) δ 152.0, 136.4, 135.7, 135.6, 129.4, 128.84, 128.81, 125.92, 125.87, 122.2, 121.7, 114.0, 43.7, 40.1, 36.4, 25.4. GCMS (EI) *m*/*z*: [M]^+^ calcd for C_16_H_16_ 208.1; found 208.1.

#### Dimethyl (2*R*,3*R*)-1,2,3,4-tetrahydronaphthalene-2,3-dicarboxylate (**6a**)^47^

**6a** was prepared according to general procedure A. A pale red oil (174 mg, 70% yield) was collected after purification by auto column chromatography (0–20% ethyl acetate in hexane). ^1^H NMR (400 MHz, CDCl_3_) δ: 7.19–7.05 (m, 4H), 3.74 (s, 6H), 3.21–3.02 (m, 4H), 3.00–2.89 (m, 2H). ^13^C{^1^H} NMR (101 MHz, CDCl_3_) δ: 175.1, 133.9, 128.8, 126.5, 52.2, 42.2, 31.9. GCMS (EI) *m*/*z*: [M]^+^ calcd for C_14_H_16_O_4_ 248.1; found 248.1.

#### Diethyl (2*R*,3*R*)-1,2,3,4-Tetrahydronaphthalene-2,3-dicarboxylate (**6b**)^37^

**6b** was prepared according to general procedure A. A yellow oil (155 mg, 56% yield) was collected after purification by auto column chromatography (0–20% ethyl acetate in hexane). ^1^H NMR (400 MHz, CDCl_3_) δ 7.17–7.07 (m, 4H), 4.19 (m, 4H), 3.15 (m, 2H), 3.04 (m, 2H), 2.99–2.88 (m, 2H), 1.28 (t, *J* = 7.1 Hz, 6H). ^13^C{^1^H} NMR (101 MHz, CDCl_3_) δ: 174.6, 134.1, 128.7, 126.5, 61.0, 42.3, 32.0, 14.3. GCMS (EI) *m*/*z*: [M]^+^ calcd for C_16_H_20_O_4_ 276.1; found 276.1.

#### (2*R*,3*R*)-1,2,3,4-Tetrahydronaphthalene-2,3-dicarbonitrile (**6c**)^48^

**6c** was prepared according to general procedure A. A pale yellow solid (80 mg, 44% yield) was collected after purification by auto column chromatography (0–10% ethyl acetate in hexane). ^1^H NMR (400 MHz, CDCl_3_) δ: 7.25–7.20 (m, 2H), 7.14 (m, 2H), 3.44–3.28 (m, 4H), 3.17 (m, 2H). ^13^C{^1^H} NMR (101 MHz, CDCl_3_) δ: 130.3, 129.1, 127.7, 118.7, 30.6, 28.8. GCMS (EI) *m*/*z*: [M]^+^ calcd for C_12_H_10_N_2_ 182.1, found 182.1.

#### Diethyl 1,4-Dihydrophthalazine-2,3-dicarboxylate (**6d**)^49^

**6d** was prepared according to general procedure A. A pale yellow solid (120 mg, 43% yield) was collected after purification by auto column chromatography (0–20% ethyl acetate in hexane). ^1^H NMR (400 MHz, CDCl_3_) δ 7.24 (s, 2H), 7.13 (s, 2H), 5.20–4.84 (m, 2H), 4.41 (s, 2H), 4.31–4.01 (m, 4H), 1.27 (t, *J* = 7.2 Hz, 6H). ^13^C{^1^H} NMR (101 MHz, CDCl_3_) δ: 155.3, 131.2, 127.1, 62.7, 45.6, 29.8, 14.7. HRMS (ESI) *m*/*z*: [M + H]^+^ calcd for C_14_H_19_N_2_O_4_ 279.1339; found 279.1346.

#### (3*aR*,9*aS*)-2-Methyl-2,3,3*a*,4,9,9*a*-hexahydro-1*H*-benzo [*F*]isoindole (**6e**)^50^

**6e** was prepared according to general procedure A. A pale yellow solid (116 mg, 54% yield) was collected after purification by auto column chromatography (0–20% ethyl acetate in hexane). The single crystal was obtained by diffusing hexane into **6e** solution in chloroform. ^1^H NMR (600 MHz, CDCl_3_) δ 7.19–7.15 (m, 2H), 7.15–7.11 (m, 2H), 3.30–3.24 (m, 2H), 3.11 (dd, *J* = 14.8, 2.6 Hz, 2H), 2.95–2.88 (m, 2H), 2.77 (s, 3H). ^13^C{^1^H} NMR (101 MHz, CDCl_3_) δ: 179.4, 135.1, 127.9, 127.3, 40.1, 29.5, 24.8. GCMS (EI) *m*/*z*: [M]^+^ calcd for C_13_H_13_NO_2_ 215.1; found 215.1.

#### Ethyl 1,2,3,4-Tetrahydronaphthalene-2-carboxylate (**6f**)^37^

**6f** was prepared according to general procedure A. A pale yellow oil (62 mg, 30% yield) was collected after purification by auto column chromatography (0–10% ethyl acetate in hexane). ^1^H NMR (400 MHz, CDCl_3_) δ 7.13 (m, 4H), 4.21 (q, *J* = 7.1 Hz, 2H), 3.04 (d, *J* = 8.2 Hz, 2H), 2.90 (m, 2H), 2.80–2.69 (m, 1H), 2.30–2.18 (m, 1H), 1.96–1.81 (m, 1H), 1.32 (t, *J* = 7.1 Hz, 3H). ^13^C{^1^H} NMR (101 MHz, CDCl_3_) δ: 175.6, 135.9, 135.1, 129.2, 129.0, 126.0, 125.9, 60.6, 40.2, 31.8, 28.7, 26.0, 14.4. GCMS (EI) *m*/*z*: [M]^+^ calcd for C_13_H_16_O_2_ 204.1; Found 204.1.

#### 1,2,3,4-Tetrahydronaphthalene-2-carbonitrile (**6g**)^37^

**6g** was prepared according to general procedure A. A light yellow solid (50 mg, 32% yield) was collected after purification by auto column chromatography (0–10% ethyl acetate in hexane). ^1^H NMR (400 MHz, CDCl_3_) δ: 7.22–7.04 (m, 4H), 3.20–2.95 (m, 4H), 2.86 (m, 1H), 2.21 (m, 1H), 2.08 (m, 1H). ^13^C{^1^H} NMR (101 MHz, CDCl_3_) δ 134.6, 132.4, 129.2, 129.1, 126.81, 126.4, 122.2, 32.4, 27.1, 26.4, 25.6. GCMS (EI) *m*/*z*: [M]^+^ calcd for C_11_H_11_N 157.1; found 157.1.

#### Diethyl 3-Methyl-3,4-dihydronaphthalene-2,2(1*H*)-dicarboxylate (**6h**)

**6h** was prepared according to general procedure A. A pale yellow oil (38 mg, 13% yield) was collected after purification by auto column chromatography (0–10% ethyl acetate in hexane). ^1^H NMR (400 MHz, CDCl_3_) δ 7.10 (m, 3H), 7.04 (m, 1H), 4.24–4.08 (m, 4H), 3.31 (s, 2H), 3.10 (dd, *J* = 17.0, 5.7 Hz, 1H), 2.75 (m, 1H), 2.64 (dd, *J* = 17.0, 6.2 Hz, 1H), 1.21 (m, 6H), 1.10 (d, *J* = 6.9 Hz, 3H). ^13^C{^1^H} NMR (101 MHz, CDCl_3_) δ: 171.4, 170.6, 134.4, 133.4, 129.1, 128.8, 126.2, 125.9, 61.5, 61.3, 57.6, 34.4, 33.3, 32.7, 17.0, 14.2, 14.1. HRMS (ESI) *m*/*z*: [M + H]^+^ calcd for C_17_H_23_O_4_ 291.1591; found 291.1601.

#### Dimethyl 5-Fluoro-1,2,3,4-tetrahydronaphthalene-2,3-dicarboxylate (**6i**)

**6i** was prepared according to general procedure B. A pale yellow solid (77 mg, 58% yield) was collected after purification by auto column chromatography (0–20% ethyl acetate in hexane). ^1^H NMR (400 MHz, CDCl_3_) δ 7.11 (q, *J* = 7.2 Hz, 1H), 6.87 (m, 2H), 3.74 (dd, *J* = 3.6, 1.7 Hz, 6H), 3.31–3.21 (m, 1H), 3.19–3.10 (m, 1H), 3.04 (m, 2H), 2.92 (dm, 1H), 2.77 (m, 1H). ^13^C{^1^H} NMR (101 MHz, CDCl_3_) δ: 174.8, 161.9, 159.5, 136.4, 127.4, 127.3, 124.13, 124.10, 121.7, 121.6, 112.9, 112.7, 52.34, 52.32, 41.7, 41.4, 31.60, 31.57, 24.92, 24.88. ^19^F{^1^H} NMR (376 MHz, CDCl_3_) δ: −117.91, −117.93, −117.94, −117.95. HRMS (ESI) *m*/*z*: [M + H]^+^ calcd for C_14_H_16_FO_4_ 267.1027; found 267.1037.

#### Dimethyl 5-Chloro-1,2,3,4-tetrahydronaphthalene-2,3-dicarboxylate (**6j**)

**6j** was prepared according to general procedure B. A white solid (82 mg, 58% yield) was collected after purification by auto column chromatography (0–20% ethyl acetate in hexane). ^1^H NMR (400 MHz, CDCl_3_) δ 7.21 (d, *J* = 7.8 Hz, 1H), 7.08 (t, *J* = 7.7 Hz, 1H), 7.00 (d, *J* = 7.6 Hz, 1H), 3.74 (d, *J* = 7.6 Hz, 6H), 3.30 (dd, *J* = 17.1, 5.1 Hz, 1H), 3.13 (m, 1H), 3.07–2.92 (m, 3H), 2.79 (m, 1H). ^13^C{^1^H} NMR (101 MHz, CDCl_3_) δ: 174.70, 174.68, 136.2, 134.3, 131.9, 127.4, 127.2, 127.19, 52.32, 52.27, 42.0, 41.6, 32.2, 29.7. HRMS (ESI) *m*/*z*: [M + H]^+^ calcd for C_14_H_16_ClO_4_ 283.0732; found 283.0743.

#### Diethyl 5-Hydroxy-1,2,3,4-tetrahydronaphthalene-2,3-dicarboxylate (**6k**)

**6k** was prepared according to general procedure B. A white solid (90 mg, 68% yield) was collected after purification by auto column chromatography (0–20% ethyl acetate in hexane). ^1^H NMR (600 MHz, CDCl_3_) δ 7.01 (t, *J* = 7.8 Hz, 1H), 6.69 (d, *J* = 7.6 Hz, 1H), 6.61 (d, *J* = 7.9 Hz, 1H), 5.31 (br, 1H), 3.74 (s, 6H), 3.24 (dd, *J* = 16.8, 4.8 Hz, 1H), 3.12 (dd, *J* = 16.0, 4.0 Hz, 1H), 3.02 (m, 2H), 2.92 (m, 1H), 2.72–2.62 (m, 1H). ^13^C{^1^H} NMR (101 MHz, CDCl_3_) δ: 175.4, 175.3, 153.4, 135.7, 127.0, 121.0, 120.9, 112.6, 52.3, 41.94, 41.89, 32.1, 26.0. HRMS (ESI) *m*/*z*: [M + H]^+^ calcd for C_14_H_17_O_5_ 265.1071; found 265.1081.

#### Diethyl 5-Bromo-1,2,3,4-tetrahydronaphthalene-2,3-dicarboxylate (**6l**)

**6l** was prepared according to the general procedure B, except that diethyl fumarate (103 mg, 0.6 mmol, 1.2 equiv) was used. A pale yellow oil (87 mg, 49% yield) was collected after purification by auto column chromatography (0–20% ethyl acetate in hexane). ^1^H NMR (400 MHz, CDCl_3_) δ 7.40 (d, *J* = 7.7 Hz, 1H), 7.02 (m, 2H), 4.27–4.05 (m, 4H), 3.26 (m, 1H), 3.12 (m, 1H), 3.05–2.87 (m, 3H), 2.78 (m, 1H), 1.28 (q, *J* = 7.4 Hz, 6H). ^13^C{^1^H} NMR (101 MHz, CDCl_3_) δ: 174.2, 174.1, 136.6, 133.7, 130.7, 127.9, 127.6, 125.1, 61.1, 61.0, 42.5, 41.8, 32.6, 32.5, 14.3. HRMS (ESI) *m*/*z*: [M + H]^+^ calcd for C_16_H_20_BrO_4_ 355.0539; found 355.0556.

#### Dimethyl 6-Fluoro-1,2,3,4-tetrahydronaphthalene-2,3-dicarboxylate (**6m**)^51^

**6m** was prepared according to general procedure B. A pale yellow oil (65 mg, 49% yield) was collected after purification by auto column chromatography (0–10% ethyl acetate in hexane). ^1^H NMR (400 MHz, CDCl_3_) δ 7.03 (m, 1H), 6.88–6.72 (m, 2H), 3.72 (d, *J* = 1.1 Hz, 6H), 3.15–2.98 (m, 4H), 2.90 (m, 2H). ^13^C{^1^H} NMR (101 MHz, CDCl_3_) δ: 174.73, 174.69, 162.6, 160.1, 135.9, 135.8, 130.1, 130.0, 129.5, 129.4, 115.0, 114.8, 113.8, 113.5, 52.22, 52.19, 42.1, 41.8, 31.7, 31.7, 31.0. ^19^F{^1^H} NMR (376 MHz, CDCl_3_) δ −116.73, −116.75. GCMS (EI) *m*/*z*: [M]^+^ calcd for C_14_H_15_FO_4_ 266.1; found 266.1.

#### Dimethyl 6-Chloro-1,2,3,4-tetrahydronaphthalene-2,3-dicarboxylate (**6n**)^51^

**6n** was prepared according to general procedure B. A white solid (97 mg, 69% yield) was collected after purification by auto column chromatography (0–10% ethyl acetate in hexane). ^1^H NMR (400 MHz, CDCl_3_) δ 7.16–6.97 (m, 3H), 3.73 (d, *J* = 1.7 Hz, 6H), 3.15–3.02 (m, 4H), 2.99–2.83 (m, 2H). ^13^C{^1^H} NMR (101 MHz, CDCl_3_) δ: 174.69, 174.66, 135.7, 132.4, 132.0, 130.0, 128.5, 126.7, 52.4, 52.3, 41.9, 41.8, 31. 5, 31.1. GCMS (EI) *m*/*z*: [M]^+^ calcd for C_14_H_15_ClO_4_ 282.1; found 282.1.

#### Dimethyl 6-Bromo-1,2,3,4-tetrahydronaphthalene-2,3-dicarboxylate (**6o**)

**6o** was prepared according to general procedure B. A pale orange solid (108 mg, 66% yield) was collected after purification by auto column chromatography (0–10% ethyl acetate in hexane). ^1^H NMR (400 MHz, CDCl_3_) δ 7.24 (d, *J* = 7.9 Hz, 2H), 6.96 (d, *J* = 8.0 Hz, 1H), 3.73 (d, *J* = 1.8 Hz, 6H), 3.13–2.82 (m, 6H). ^13^C{^1^H} NMR (101 MHz, CDCl_3_) δ 174.63, 174.59, 136.2, 132.9, 131.4, 130.3, 129.6, 120.0, 52.30, 52.28, 41.8, 41.7, 31.3, 31.1. HRMS (ESI) *m*/*z*: [M + H]^+^ Calcd for C_14_H_16_BrO_4_ 327.0226; Found 327.0238.

#### Dimethyl 6-Nitro-1,2,3,4-tetrahydronaphthalene-2,3-dicarboxylate (**6p**)

**6p** was prepared according to general procedure B. A pale yellow oil (88 mg, 60% yield) was collected after purification by auto column chromatography (0–20% ethyl acetate in hexane). ^1^H NMR (400 MHz, CDCl_3_) δ 7.99 (d, *J* = 4.1 Hz, 2H), 7.26–7.22 (m, 1H), 3.74 (d, *J* = 2.3 Hz, 6H), 3.26–3.11 (m, 4H), 3.10–3.00 (m, 2H). ^13^C{^1^H} NMR (101 MHz, CDCl_3_) δ: 174.2, 174.1, 146.7, 141.8, 135.6, 129.7, 123.8, 121.6, 52.50, 52.49, 41.39, 41.36, 31.4, 31.2. HRMS (ESI) *m*/*z*: [M + H]^+^ calcd for C_14_H_16_NO_6_ 294.0972; found 294.0980.

#### Trimethyl 1,2,3,4-Tetrahydronaphthalene-2,3,6-tricarboxylate (**6q**)

**6q** was prepared according to general procedure B. A light yellow oil (101 mg, 66% yield) was collected after purification by auto column chromatography (0–20% ethyl acetate in hexane). ^1^H NMR (400 MHz, CDCl_3_) δ 7.74 (s, 2H), 7.11 (d, *J* = 9.0 Hz, 1H), 3.85 (s, 3H), 3.70 (s, 6H), 3.18–3.09 (m, 2H), 3.07–2.99 (m, 2H), 2.99–2.88 (m, 2H). ^13^C{^1^H} NMR (101 MHz, CDCl_3_) δ: 174.52, 174.51, 166.9, 139.3, 134.1, 129.9, 128.7, 128.4, 127.4, 52.2, 52.1, 41.8, 41. 7, 31.7, 31.4. HRMS (ESI) *m*/*z*: [M + H]^+^ calcd for C_16_H_19_O_6_ 307.1176; found 307.1188.

#### Dimethyl 6-Methyl-1,2,3,4-tetrahydronaphthalene-2,3-dicarboxylate (**6r**)^51^

**6r** was prepared according to general procedure B. A white solid (65 mg, 50% yield) was collected after purification by auto column chromatography (0–20% ethyl acetate in hexane). ^1^H NMR (400 MHz, CDCl_3_) δ 7.03–6.88 (m, 3H), 3.73 (d, *J* = 1.6 Hz, 6H), 3.17–2.99 (m, 4H), 2.90 (dt, *J* = 18.1, 5.6 Hz, 2H), 2.29 (s, 3H). ^13^C{^1^H} NMR (101 MHz, CDCl_3_) δ: 175.2, 136.1, 133.7, 130.8, 129.3, 128.6, 127.4, 52.2, 42.4, 42.3, 31.9, 31.6, 21.1. GCMS (EI) *m*/*z*: [M]^+^ calcd for C_15_H_18_O_4_ 262.1; found 262.1.

## Data Availability

The data underlying this study are available in the published article and its Supporting Information.

## Supporting Information Available

General information; synthesis of *N*-(benzyloxy)-*N*-(pivaloyloxy)-4-(trifluoromethyl)-benzamide (anomeric amide); general procedure for isoindoline hydrochloride neutralization; dimethyl maleate isomerization; LCMS of the TEMPO trapping experiment; computational methods; computational data: Cartesian coordinates, SCF energies, free energies, and transition state imaginary frequencies; NMR spectra; single crystal X-ray diffraction of **6e** (PDF)
