# Supplementary material for: Synthesis of Substituted Tetralins via Nitrogen Deletion/Diels–Alder Cascade Reaction
Source: J Org Chem. 2024 Nov 22;89(23):17338–45. doi: 10.1021/acs.joc.4c01959 (PMC11629391; doi:10.1021/acs.joc.4c01959)
Supplement: Supplementary file 1 — jo4c01959_si_001.pdf [file jo4c01959_si_001.pdf]

Supporting Information

Synthesis of Substituted Tetralins via Nitrogen Deletion/Diels-Alder Cascade Reaction

Zixuan Zang, Wen Ye, Kehang Cheng, Xiaotai Wang and Xiaodong Jin*

Department of Chemistry, Xi'an Jiaotong-Liverpool University, Suzhou, Jiangsu Province, China

Email: xiaodong.jin@xjtlu.edu.cn

Contents

1. **General Information** S2

2. **Synthesis of *N*-(Benzyloxy)-*N*-(pivaloyloxy)-4-(trifluoromethyl)-benzamide (Anomeric amide)** S3

   N-(benzyloxy)-4-(trifluoromethyl)benzamide) S3

   N-(benzyloxy)-N-(pivaloyloxy)-4-(trifluoromethyl)benzamide) S3

3. **General Procedure for Isoindoline Hydrochloride Neutralization** S4

4. **Dimethyl Maleate Isomerization** S4

5. **LCMS of the TEMPO Trapping Experiment** S5

6. **Computational Methods** S5

7. **Computational Data: Cartesian Coordinates, SCF Energies, Free Energies, and Transition State Imaginary Frequencies** S6

8. **References** S10

9. **NMR Spectra** S10

10. **Single Crystal X-ray Diffraction of 6e** S58

1. General Information

All the reactions were performed in oven-dried glassware under nitrogen. Anhydrous acetonitrile (MeCN), dichloromethane (DCM) and tetrahydrofuran (THF) were obtained from a solvent tower using the PureSolv solvent purification system, degassed under nitrogen. Unless noted otherwise, all chemicals were purchased from Sigma-Aldrich or Adamas reagent and used as received without further purification. Anomeric amide and isoindoline were stored in a freezer at -20 °C. Analytical thin-layer chromatography (TLC) was carried out using silica gel plates (Merck silica gel 60, F254) and visualized by UV light or stain. Automatic chromatography was conducted using Biotage Isolera Four Flash Chromatography System and the column used were Biotage Rening pre-packed cartridges (40-63 μm, 10 g or 30 g). Nuclear magnetic resonance ^{1}H NMR, ^{19}F NMR, ^{13}C NMR and DEPT NMR spectra were recorded with Bruker AVANCE III 400 spectrometer operating at 400MHz for ^{1}H NMR. The chemical shifts will be recorded in parts per million (ppm, δ) and are referenced to solvent $CDCl_3$ (7.26 ppm, ^{1}H and 77.160 ppm, ^{13}C) and DMSO-d_6 (2.50 ppm, ^{1}H and 39.53 ppm, ^{13}C). ^{1}H NMR will be reported as follows: ^{1}H NMR (400 MHz, solvent) δ chemical shift (shape of the peak, coupling constant (Hz), number of H atoms). Mass spectra were recorded on Agilent 8860/5977C Series GC/MSD System and 1260 Infinity II/InfinityLab LC/MSD series. Supercritical fluid chromatography data were collected using Shimadzu Nexera UC/SFC-30A with an SPD-M20A detector.

Safety Note: No issues were found in our group with handling of the anomeric amide. Safety concerns have been raised by Levin's group, as many members of this class of compounds have been experimentally demonstrated to behave as direct-acting mutagens in bacteria.[1,2] We advise appropriate caution in the handling of anomeric amide following the suggestion from Levin's group: standard chemical safety protocols to minimize exposure and prevent accidental ingestion, inhalation, or dermal contact, including proper ventilation and personal protective equipment.

2. Synthesis of *N*-(Benzyloxy)-*N*-(pivaloyloxy)-4-(trifluoromethyl)-benzamide (Anomeric amide)

Anomeric amide was prepared according to previous publication with some modifications.[1,3]

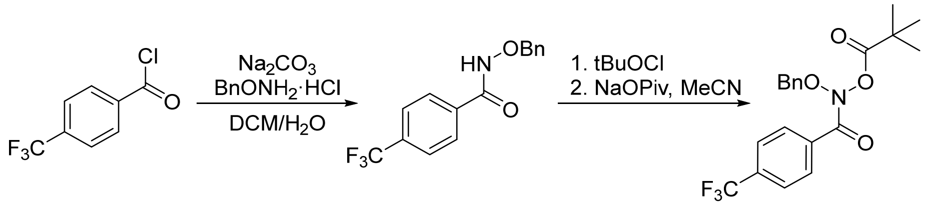

N-(benzyloxy)-4-(trifluoromethyl)benzamide)

O-benzyl hydroxylamine hydrochloride (27.95 g, 0.175 mol, 1 eq.) and Na_2CO_3 (37.1 g, 0.35 mol, 2 eq.) were weighed into a 2 L round bottom flask with a stir bar. DCM and DI water (0.7 L, 4:1, v: v) were added to the flask and well agitated with reagents. After cooling the mixture for 20 min using ice bath, trifluoromethyl benzoyl chloride (26 mL, 0.175 mol, 1 eq.) was added dropwise using a constant pressure funnel. The mixture was warmed to room temperature and stirred for 5 hours, and then DCM was removed by rotavapor. After diethyl ether (Et_2O, 300 mL) was added to the remaining suspension, the liquid part was separated by filtration. The white crystals obtained were washed with 100 mL of D.I. water and 100 mL of Et_2O, respectively, then dried under vacuum to afford hydroxamate O-Benzyl ester (47.77 g, 0.162 mol, 92.4%). ^{1}H NMR (400 MHz, $CDCl_3$) δ 7.79 (d, J = 8.0 Hz, 2H), 7.67 (d, J = 8.0 Hz, 2H), 7.50 – 7.33 (m, 5H), 5.07 (s, 2H). ^{19}F NMR (376 MHz, $CDCl_3$) δ -63.08. ^{13}C NMR (101 MHz, DMSO-d_6) δ 129.0, 128.4, 128.4, 128.1, 125.5, 77.1. Data are in agreement with those in the literature.[1]

N-(benzyloxy)-N-(pivaloyloxy)-4-(trifluoromethyl)benzamide)

To a well-mixed suspension of hydroxamate O-Benzyl ester (27.16 g, 92 mmol, 1 eq.) and degassed DCM (200 mL) in a 500 mL round bottom flask, tert-butyl hypochlorite (15.5 mL, 137.3 mmol, 1.5 eq.) was added using a syringe. The reaction was carried out under N_2 protection and shielded from ambient light at room temperature. The consumption of ester needed, monitored by TLC, took about 2 hours. The solution was concentrated under vacuum to afford N-chloroamide as a yellow oil. Sodium pivalate hydrate (18.0 g, 126.6 mmol, 1.4 eq.) and 3 Å molecular sieve (5 g) were stirred for 20 minutes in degassed dry MeCN (150 mL) followed by the addition of N-chloroamide solution (dissolved in dry 75 mL MeCN). The reaction was protected under N_2, shielded from light and stirred under ambient temperature for 2 hours. After the completion of the reaction monitored by TLC, Et_2O (300 mL) was added and the mixture was filtered over a silica gel plug. The obtained solution was concentrated in rotavapor to afford pale yellow oil and solidified

to a pale yellow solid in freezer at -20 °C. (26.71 g, 67.6 mmol, 73.4%). ^{1}H NMR (400 MHz, $CDCl_3$) δ 7.76 (d, J = 8.5 Hz, 2H), 7.64 (d, J = 8.4 Hz, 2H), 7.41 – 7.33 (m, 5H), 5.13 (s, 2H), 1.09 (s, 9H). ^{19}F NMR (376 MHz, $CDCl_3$) δ - 63.15. ^{13}C NMR (101 MHz, $CDCl_3$) δ 175.4, 173.4, 135.6, 134.9, 129.44, 129.36, 129.0, 128.7, 125.3, 125.2, 125.2, 125.2, 78.0, 38.5, 26.7. Data are in agreement with those in the literature.[1]

3. General Procedure for Isoindoline Hydrochloride Neutralization

The isoindoline hydrochloride (1 g, 6.4 mmol) was added to a 10 mL of 0.1 M NaOH solution. The reaction was left to stir for 5 minutes and then extracted three times with DCM. The organic layers were combined and removed under vacuum to yield isoindoline. The product was used without further purification and stored in dry THF as a stock solution in the glovebox.

4. Dimethyl Maleate Isomerization

The isoindoline (18 mg, 0.15 mmol, 1.0 eq.) and dimethyl maleate (22 mg, 0.15 mmol, 1.0 eq.) were dissolved in degassed anhydrous THF (0.5 mL). The mixture was stirred at 45 °C in THF for 20 hours. The solvent was removed under vacuum and the crude product was used for NMR analysis without further purification.

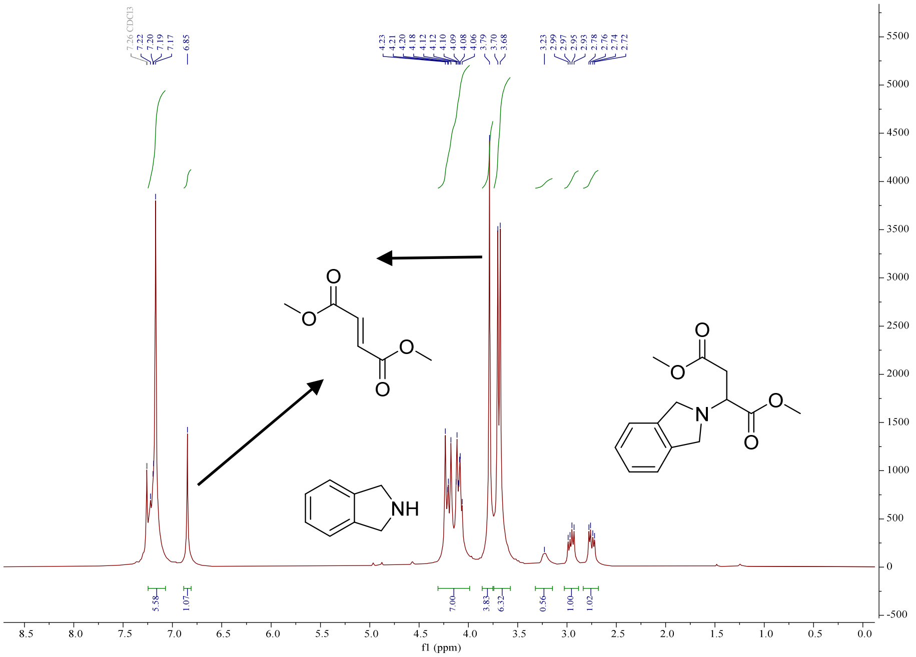

Figure S1. ^{1}H NMR spectrum of isoindoline promoted dimethyl maleate isomerization.

5. LCMS of the TEMPO Trapping Experiment

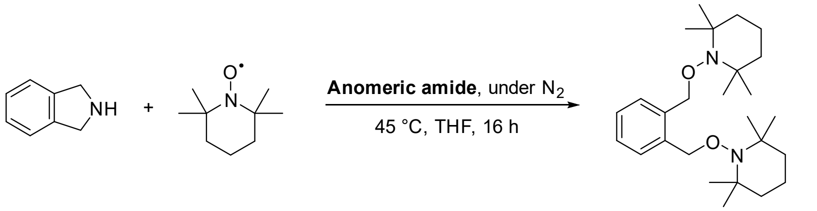

[M + H]$^+$ Calcd. for $C_{26}H_{45}N_2O_2$ 417.3; Found 417.3

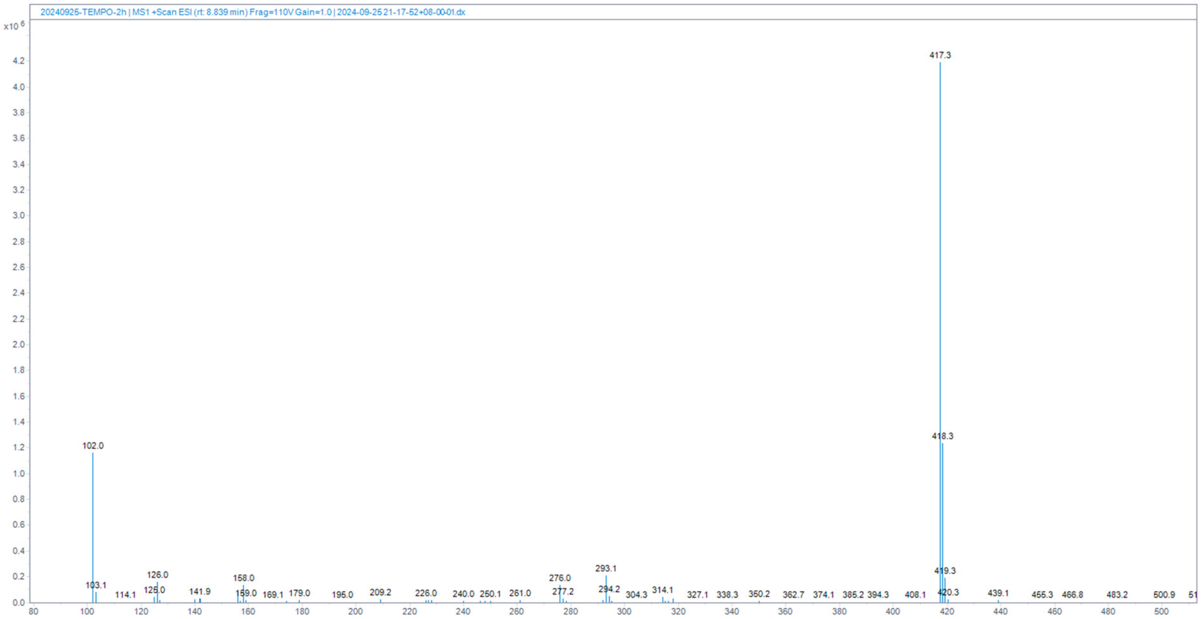

Figure S2. LCMS of the TEMPO dimer adduct.

6. Computational Methods

All density functional theory (DFT) calculations were carried out using Gaussian 09[4] with the default integration grid and convergence criteria. The global hybrid functional M06-2X was chosen because it is known to perform well for organic compounds.[5] Geometries were optimized and then characterized by frequency calculations to be energy minima (zero imaginary frequencies) or transition states (one imaginary frequency) at the M06-2X/6-311G(d,p) level in THF solution, using the 6-311G(d,p) basis set for all atoms and the SMD solvation model.[6] Zero-point vibrational energy and thermal corrections were applied to obtain Gibbs free energies at 25 °C, which were presented and referred to in all discussions.

7. Computational Data: Cartesian Coordinates, SCF Energies, Free Energies, and Transition State Imaginary Frequencies

2

SCF energy: -419.026958117 a.u.
Free energy: -418.913614 a.u.

C	2.55811800	0.69722000	-0.00263600
C	1.35847600	1.40315800	-0.00025700
C	0.16110300	0.69773300	0.00237200
C	0.16110400	-0.69773500	0.00237200
C	1.35847900	-1.40315700	-0.00025800
C	2.55811900	-0.69721700	-0.00263600
H	3.49862400	1.23576500	-0.00470800
H	1.35817900	2.48754100	-0.00049900
H	1.35818400	-2.48754000	-0.00050000
H	3.49862600	-1.23576000	-0.00470800
C	-1.23069800	-1.24285400	0.00480600
H	-1.48398800	-1.82340100	-0.88318000
C	-1.23070000	1.24285000	0.00480500
H	-1.48398700	1.82339900	-0.88318100
N	-2.11129100	0.00000100	-0.00088800
N	-3.30913300	0.00000100	-0.00938500
H	-1.48334200	-1.81479900	0.89864000
H	-1.48334200	1.81479800	0.89863800

N_2

SCF energy: -109.513826844 a.u.

Free energy: -109.526502 a.u.

N	0.00000000	0.00000000	0.54474300
N	0.00000000	0.00000000	-0.54474300

^{3}IM1

SCF energy: -309.502199208 a.u.
Free energy: -309.405159 a.u.

C	-1.87380500	-0.69128100	-0.00525800
C	-0.65593200	-1.38461800	-0.00769300
C	0.57365700	-0.72103900	-0.00095500
C	0.57367300	0.72102400	0.00097300
C	-0.65592300	1.38462300	0.00769500
C	-1.87379700	0.69129600	0.00525000
H	-2.80652600	-1.24282900	-0.00979600
H	-0.65836800	-2.46968100	-0.01141000
H	-0.65834700	2.46968400	0.01140800
H	-2.80651700	1.24284800	0.00977600
C	1.78429400	1.47699600	-0.00932500
H	2.76124600	1.02007300	-0.04973500
H	1.73427300	2.55824500	0.00123400
C	1.78429500	-1.47701100	0.00933100
H	1.73425100	-2.55826000	-0.00120400
H	2.76121400	-1.02001300	0.04962100

3

SCF energy: -309.544531451 a.u.

Free energy: -309.443042 a.u.

C	-1.82616600	0.71999600	0.11571900
C	-0.67372500	1.40670900	0.17955600
C	0.62121500	0.74597600	-0.00558700
C	0.62121600	-0.74597500	0.00559300
C	-0.67372200	-1.40671000	-0.17955700
C	-1.82616400	-0.72000000	-0.11572100
H	-2.77656800	1.23138700	0.21762300
H	-0.67128100	2.48308900	0.31665500
H	-0.67127600	-2.48309000	-0.31665700
H	-2.77656500	-1.23139300	-0.21762400
C	1.72584800	-1.47560300	0.23158200
H	2.68033400	-1.01830000	0.46520900
H	1.68447500	-2.55913100	0.20341500
C	1.72584600	1.47560600	-0.23157800
H	1.68446500	2.55913400	-0.20343700
H	2.68032600	1.01830500	-0.46523000

TS1

Imaginary frequency: –288.75

SCF energy: -619.095088948 a.u.

Free energy: -618.866695 a.u.

C	-3.45478000	-0.20234500	0.54278400
C	-2.75488300	0.92538000	0.28706900
C	-1.56073800	0.91944400	-0.53367700
C	-1.11097000	-0.36551900	-1.09964400
C	-1.87537400	-1.54899200	-0.72071000
C	-2.98680100	-1.47311400	0.04210600
C	-0.88075700	2.09226600	-0.74703000
C	-0.00558000	-0.49914800	-1.86811200
C	0.88168500	2.09294600	0.74620000
H	-0.14304900	2.18971900	-1.53193100
H	-4.35896200	-0.16740500	1.13933000
H	-3.08991700	1.88283800	0.67460100
H	-1.52479200	-2.50715000	-1.09177100
H	-3.53832300	-2.37271500	0.29202500
H	0.60091900	0.34142100	-2.17915900
C	1.56120700	0.91977200	0.53331900
C	1.11091700	-0.36470100	1.09991800
C	2.75515900	0.92478800	-0.28767700
C	1.87487600	-1.54867300	0.72167400
C	3.45456600	-0.20337400	-0.54287700
H	3.09048600	1.88189100	-0.67583700
C	2.98621200	-1.47366600	-0.04136400
H	1.52398500	-2.50647800	1.09335600
H	4.35863300	-0.16911900	-1.13963900
H	3.53737800	-2.37361200	-0.29081800
C	0.00528500	-0.49751900	1.86818300
H	-0.60088600	0.34347300	2.17870200
H	-0.29837800	-1.47858700	2.21606500

H	0.29759800	-1.48051500	-2.21556900
H	1.29377400	3.02012400	0.36169000
H	0.14410300	2.19106900	1.53114200
H	-1.29270700	3.01977400	-0.36316100

TS2

Imaginary frequency: –661.48

SCF energy: -309.497117066 a.u.

Free energy: -309.395448 a.u.

C	-1.85630100	0.70266000	0.11213300
C	-0.68630100	1.42400500	0.17608200
C	0.51646200	0.70724300	-0.02020800
C	0.51644900	-0.70724600	0.02012400
C	-0.68633100	-1.42399500	-0.17610900
C	-1.85631700	-0.70263400	-0.11211100
H	-2.80836500	1.21462300	0.19569300
H	-0.69340500	2.50338200	0.28336200
H	-0.69345600	-2.50337600	-0.28335500
H	-2.80839300	-1.21458600	-0.19559700
C	1.84418900	-1.09463100	0.32188200
C	1.84423300	1.09460800	-0.32183500
H	2.28059700	-2.02873800	-0.03067300
H	2.31288600	-0.65978400	1.19302000
H	2.28061000	2.02874300	0.03068300
H	2.31302600	0.65967600	-1.19287900

4

SCF energy: -619.194795258 a.u.

Free energy: -618.958873 a.u.

C	-3.51634400	0.46858800	0.71578400
C	-2.35704800	1.12105900	0.59923100
C	-1.11553000	0.52162500	-0.01695300
C	-1.41729400	-0.75503700	-0.80694800
C	-2.68017100	-1.44683900	-0.53517100
C	-3.66590300	-0.88197200	0.17903700
C	-0.39135300	1.60430400	-0.87892500
C	-0.53976000	-1.30348600	-1.65987500
C	0.94635600	2.07534900	-0.28000600
H	-0.20254000	1.20537000	-1.87654400
H	-4.36438700	0.93393100	1.20581700
H	-2.24259600	2.12214000	1.00806700
H	-2.80770000	-2.43306400	-0.97120400
H	-4.59831600	-1.41031000	0.34371400
H	0.40739000	-0.83999700	-1.90970600
C	1.80998700	0.88126700	0.01759600
C	1.20769700	-0.14481300	0.75901600
C	3.10924800	0.71937000	-0.45293700
C	1.90819800	-1.31886200	1.01539200
C	3.81344400	-0.45274400	-0.18149400
H	3.57143200	1.50935200	-1.03678800
C	3.21381800	-1.47131100	0.55209200
H	1.42902900	-2.11694400	1.57373800

H	4.82633700	-0.57172300	-0.54939300
H	3.75727300	-2.38691700	0.75620800
C	-0.20807100	0.09515800	1.19448900
H	-0.22862600	0.91038800	1.92709800
H	-0.63767800	-0.78733500	1.67358700
H	-0.75910100	-2.25684400	-2.13019900
H	1.44716500	2.75616400	-0.97120200
H	0.75322700	2.63302100	0.64417300
H	-1.05454700	2.46282700	-1.00932600

5

SCF energy: -309.572555206 a.u.

Free energy: -309.467646 a.u.

C	1.90828100	-0.69847100	0.00030200
C	0.71718800	-1.43548000	0.00039800
C	-0.45259400	-0.69546600	0.00004200
C	-0.45259400	0.69546600	-0.00004700
C	0.71718800	1.43548000	-0.00039900
C	1.90828100	0.69847100	-0.00030000
H	2.85733900	-1.22314600	0.00068300
H	0.73448600	-2.51959900	0.00090700
H	0.73448500	2.51959900	-0.00090600
H	2.85733900	1.22314600	-0.00067900
C	-1.96896800	0.78596800	0.00093300
C	-1.96896800	-0.78596800	-0.00093000
H	-2.40951800	1.24163200	-0.88726900
H	-2.40574900	1.23731000	0.89328200
H	-2.40951200	-1.24163200	0.88727400
H	-2.40575400	-1.23731000	-0.89327800

8. References

(1) Kennedy, S. H.; Dherange, B. D.; Berger, K. J.; Levin, M. D. Skeletal Editing through Direct Nitrogen Deletion of Secondary Amines. *Nature* **2021**, *593* (7858), 223–227.

(2) Dherange, B. D.; Yuan, M.; Kelly, C. B.; Reiher, C. A.; Grosanu, C.; Berger, K. J.; Gutierrez, O.; Levin, M. D. Direct Deaminative Functionalization. *J. Am. Chem. Soc.* **2023**, *145* (1), 17–24.

(3) Berger, K. J.; Dherange, B. D.; Morales, M; Driscoll, J. L.; Tarhan, A. K.; Levin, M. D. *N*-(Benzyloxy)-*N*-(Pivaloyloxy)-4-(Trifluoromethyl)-Benzamide. *Org. Synth.* **2023**, *100*, 113–135.

(4) Frisch, M. J.; Trucks, G. W.; Schlegel, H. B.; Scuseria, G. E.; Robb, M. A.; Cheeseman, J. R.; Scalmani, G.; Barone, V.; Men-nucci, B.; Petersson, G. A.; Nakatsuji, H.; Caricato, M.; Li, X.; Hratchian, H. P.; Izmaylov, A. F.; Bloino, J.; Zheng, G.; Sonnen-berg, J. L.; Hada, M.; Ehara, M.; Toyota, K.; Fukuda, R.; Hasegawa, J.; Ishida, M.; Nakajima, T.; Honda, Y.; Kitao, O.; Nakai, H.; Vreven, T.; Montgomery, J. A., Jr.; Peralta, J. E.; Ogliaro, F.; Bearpark, M.; Heyd, J. J.; Brothers, E.; Kudin, K. N.; Staroverov, V. N.; Kobayashi, R.; Normand, J.; Raghavachari, K.; Rendell, A.; Burant, J. C.; Iyengar, S. S.; Tomasi, J.; Cossi, M.; Rega, N.; Millam, J. M.; Klene, M.; Knox, J. E.; Cross, J. B.; Bakken, V.; Adamo, C.; Jaramillo, J.; Gomperts, R.; Stratmann, R. E.; Yazyev, O.; Austin, A. J.; Cammi, R.; Pomelli, C.; Ochterski, J. W.; Martin, R. L.; Morokuma, K.; Zakrzewski, V. G.; Voth, G. A.; Salvador, P.; Dannenberg, J. J.; Dapprich, S.; Daniels, A. D.; Farkas, O.; Foresman, J. B.; Ortiz, J. V.; Cioslowski, J.; Fox, D. J. Gaussian 09, revision D.01; Gaussian Inc.: Wallingford, CT, 2013.

(5) Zhao, Y.; Truhlar, D. G. The M06 suite of density functionals for main group thermochemistry, thermochemical kinetics, noncovalent interactions, excited states, and transition elements: Two new functionals and systematic testing of four M06-class functionals and 12 other functionals. *Theor. Chem. Acc.* **2008**, 120, 215–241.

(6) Marenich, A. V.; Cramer, C. J.; Truhlar, D. G. Universal Solvation Model Based on Solute Electron Density and on a Continuum Model of the Solvent Defined by the Bulk Dielectric Constant and Atomic Surface Tensions. *J. Phys. Chem. B* **2009**, *113*, 6378−6396.

9. NMR Spectra

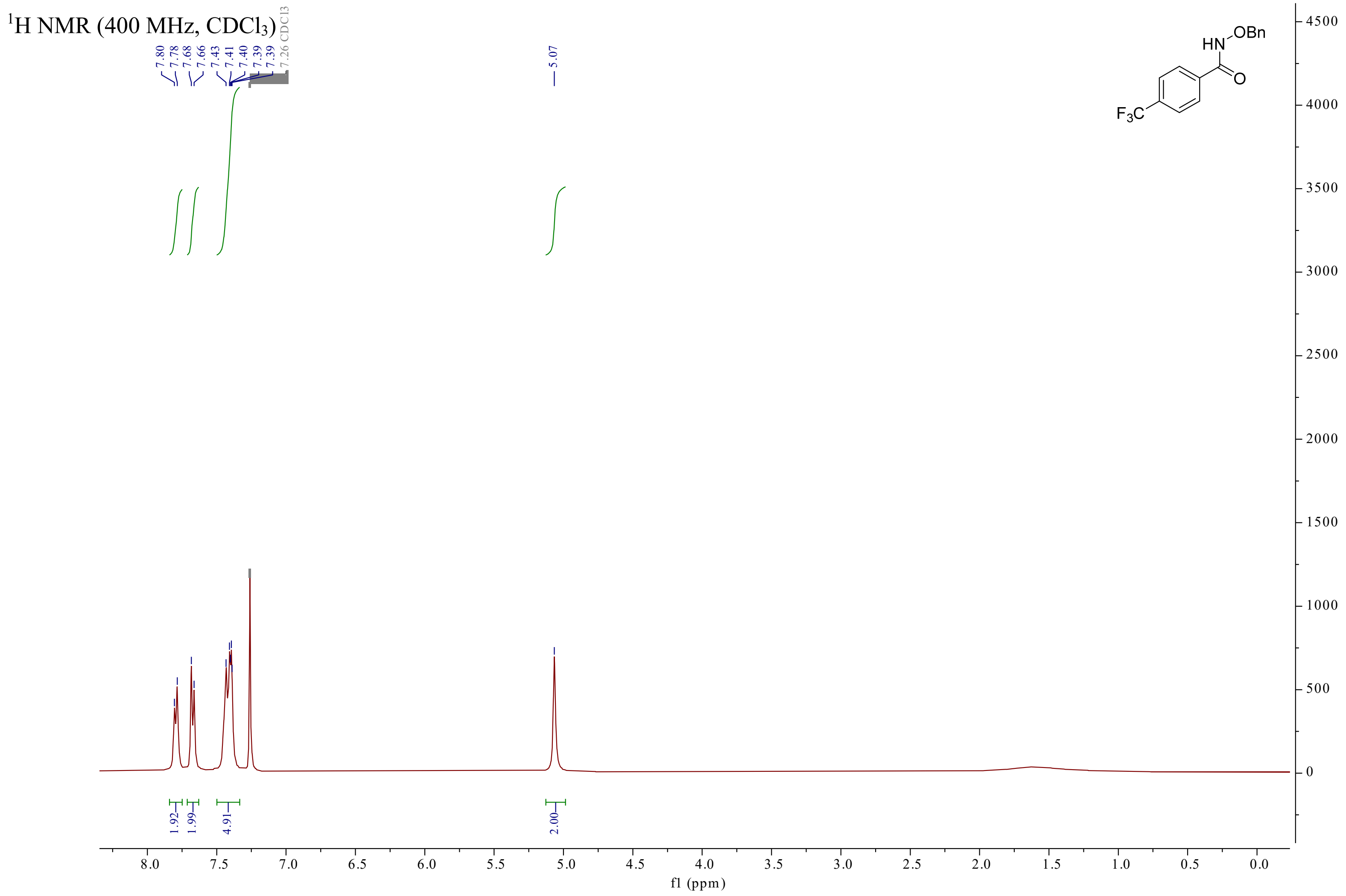

1H NMR (400 MHz, CDCl3)
7.80
7.78
7.68
7.66
7.43
7.41
7.40
7.39
7.39
7.26 CDCl3
5.07
HN
OBn
O
F3C
1.92
1.99
4.91
2.00
f1 (ppm)

^{19}F NMR (376 MHz, $CDCl_3$)

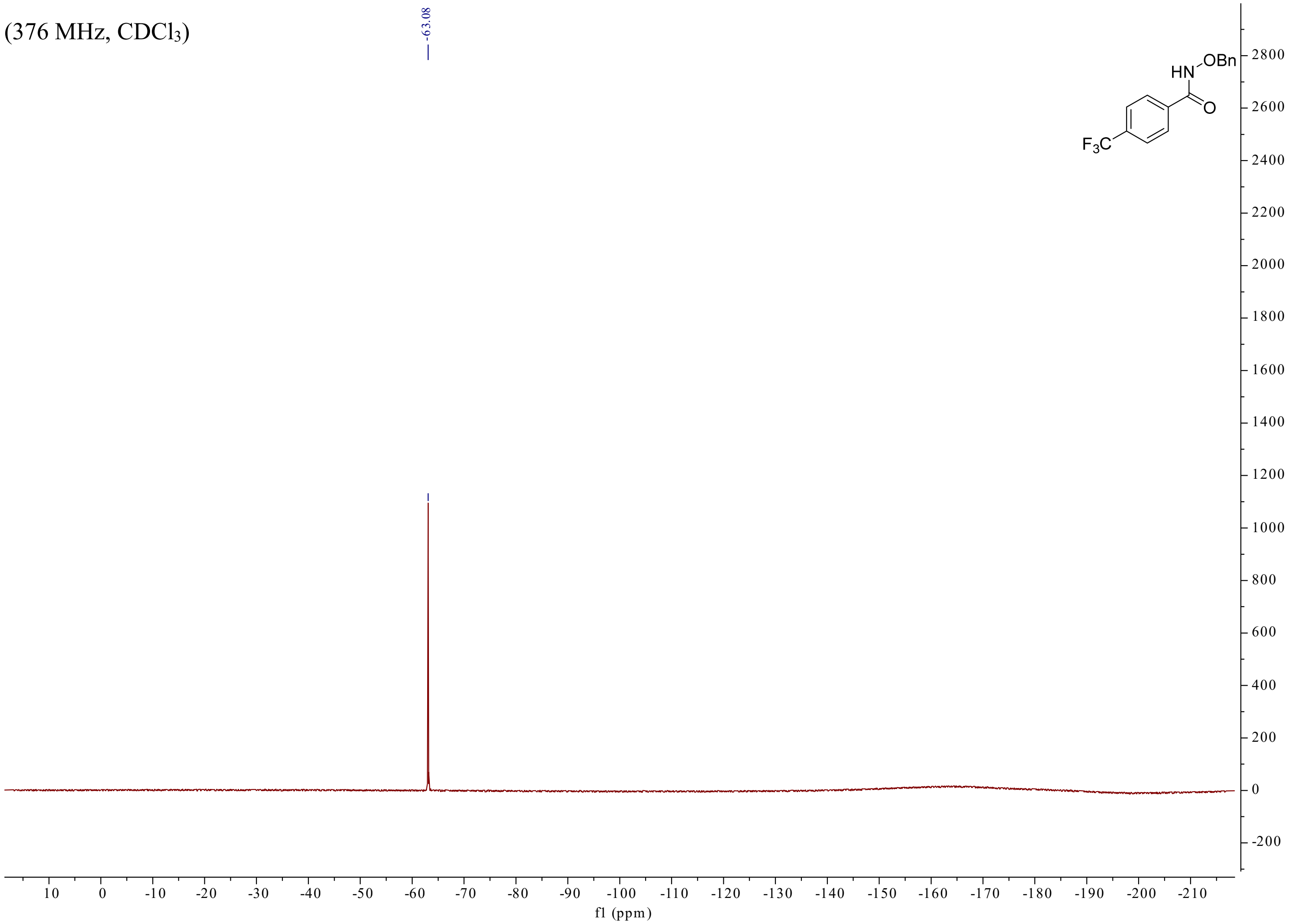

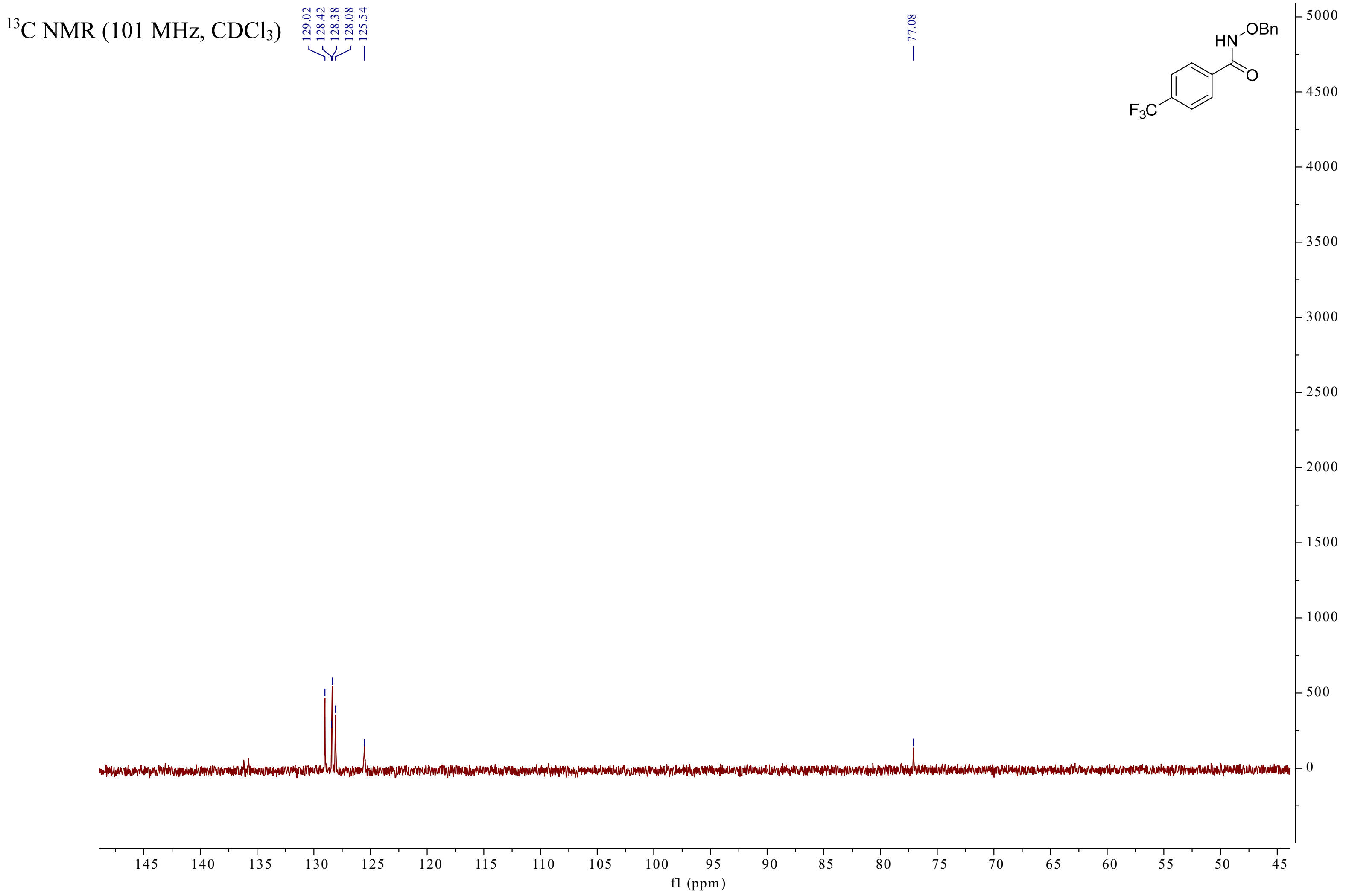

^{13}C NMR (101 MHz, CDCl$_3$)
129.02
128.42
128.38
128.08
125.54
77.08
HN
OBn
O
F$_3$C
f1 (ppm)

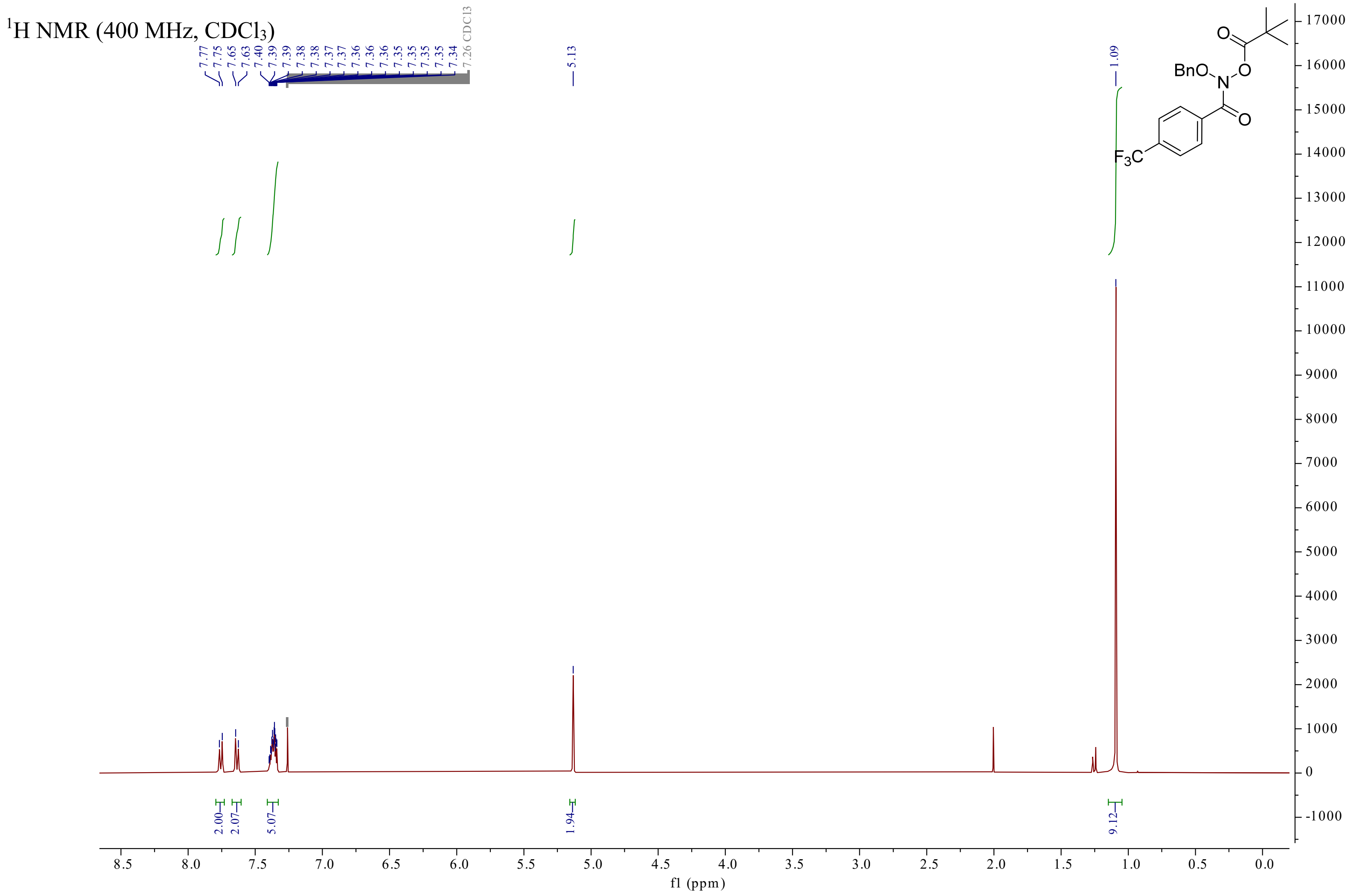

^{1}H NMR (400 MHz, $CDCl_3$)
7.77
7.75
7.65
7.63
7.40
7.39
7.39
7.38
7.38
7.37
7.37
7.36
7.36
7.35
7.35
7.35
7.35
7.34
7.26 CDCl3
5.13
1.09
BnO
N
O
O
O
F3C
2.00
2.07
5.07
1.94
9.12
fl (ppm)

19F NMR (376 MHz, $CDCl_3$)

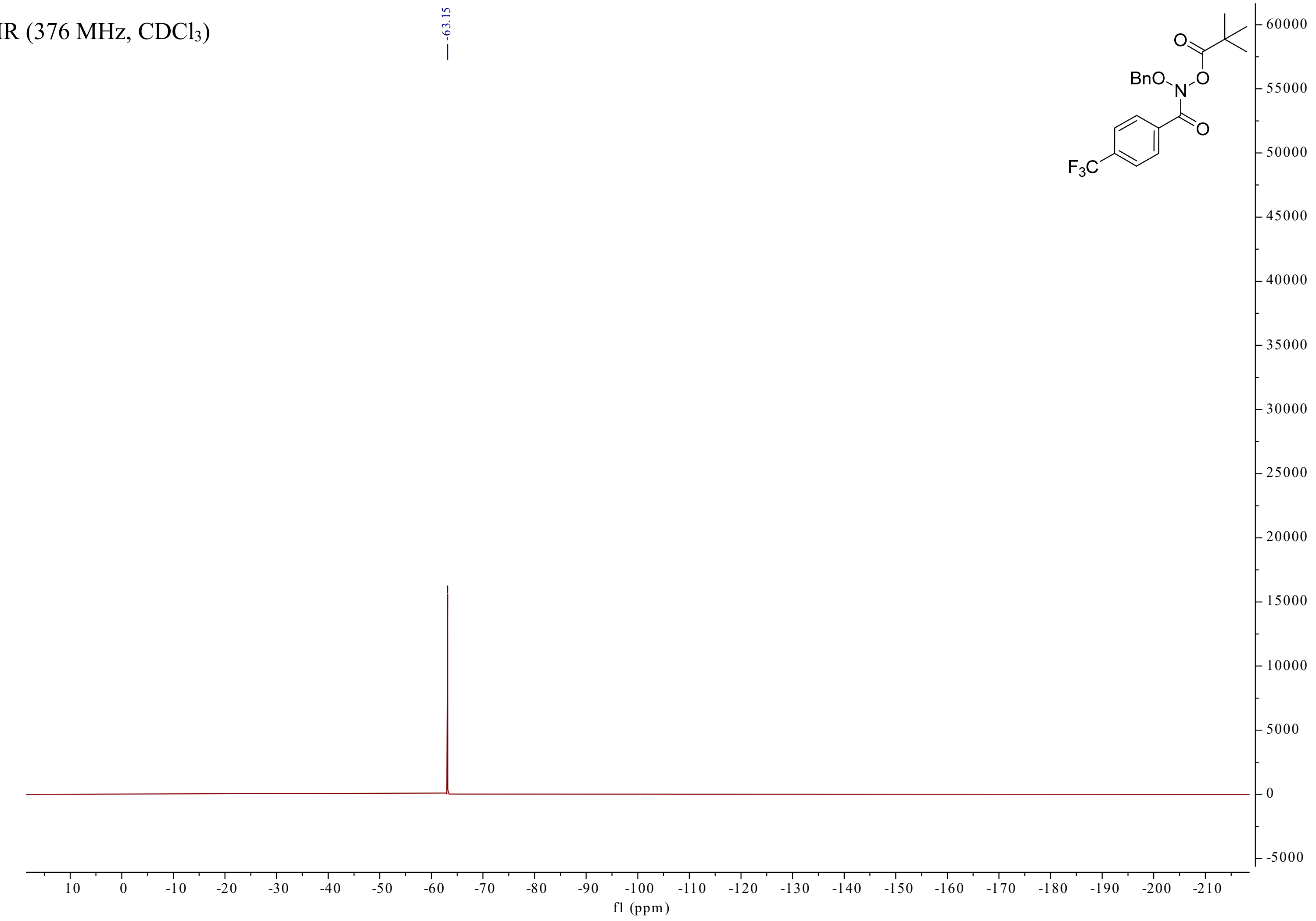

^{13}C NMR (101 MHz, $CDCl_3$)

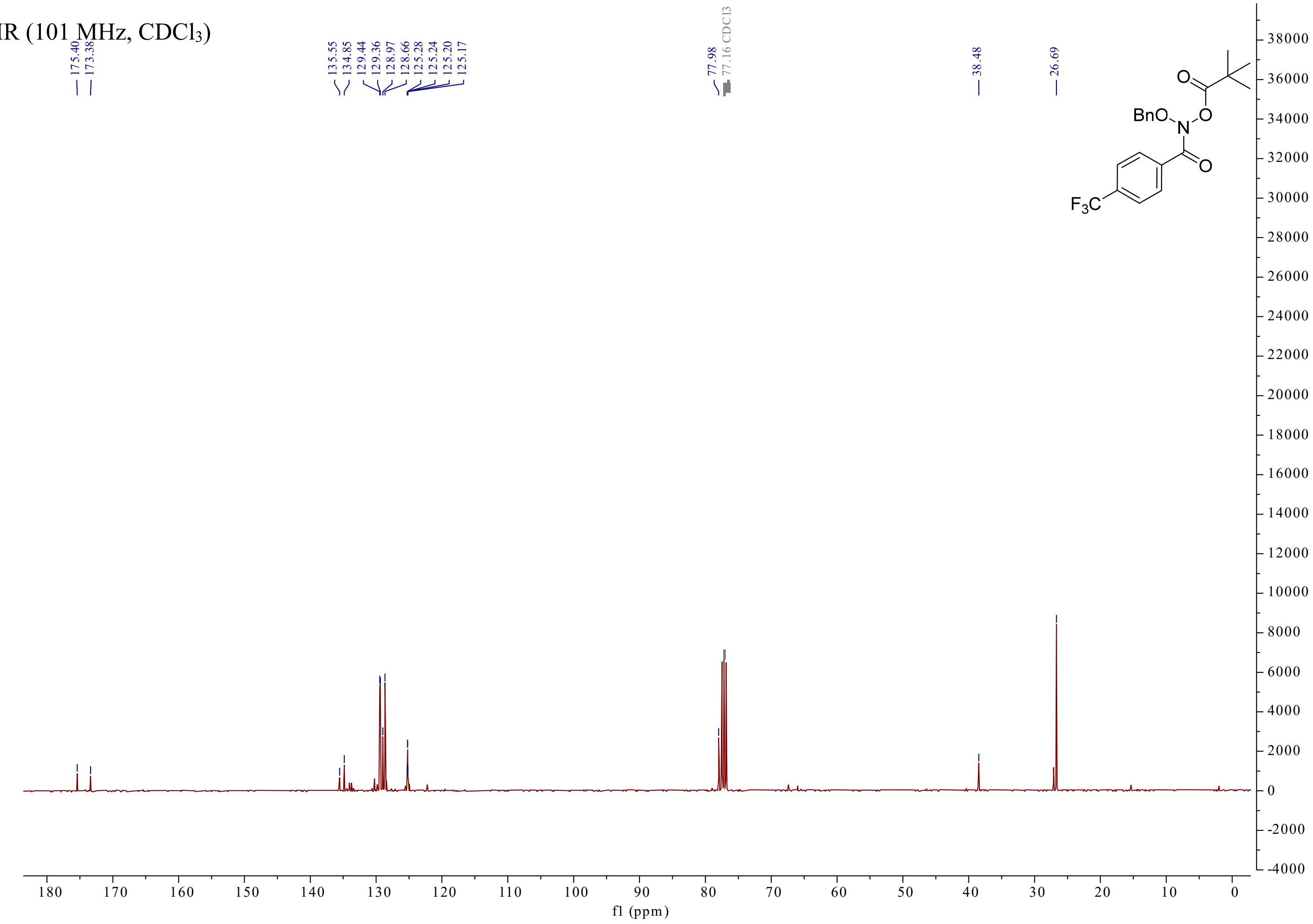

^{1}H NMR (400 MHz, $CDCl_3$)

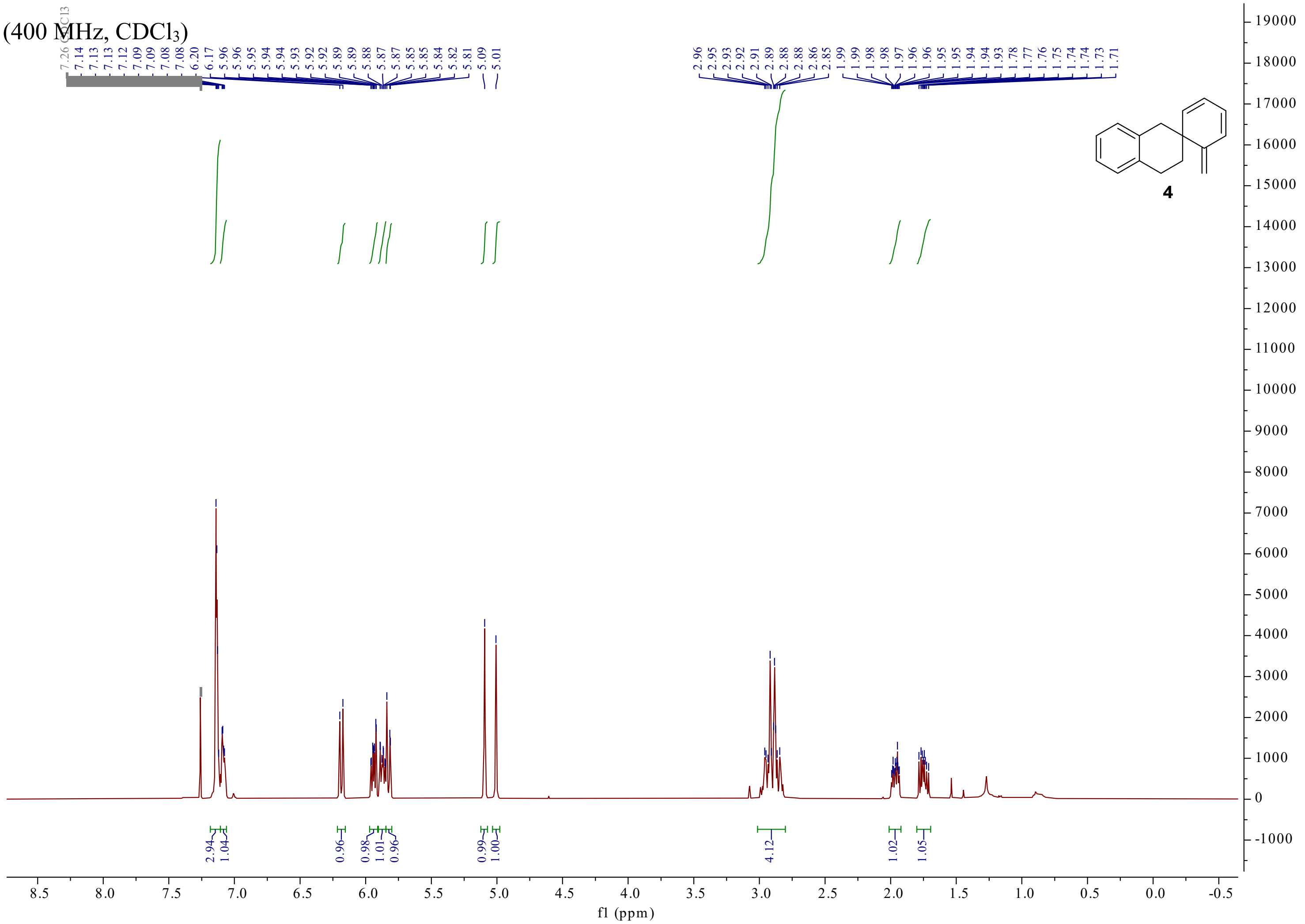

^{13}C NMR (101 MHz, $CDCl_3$)

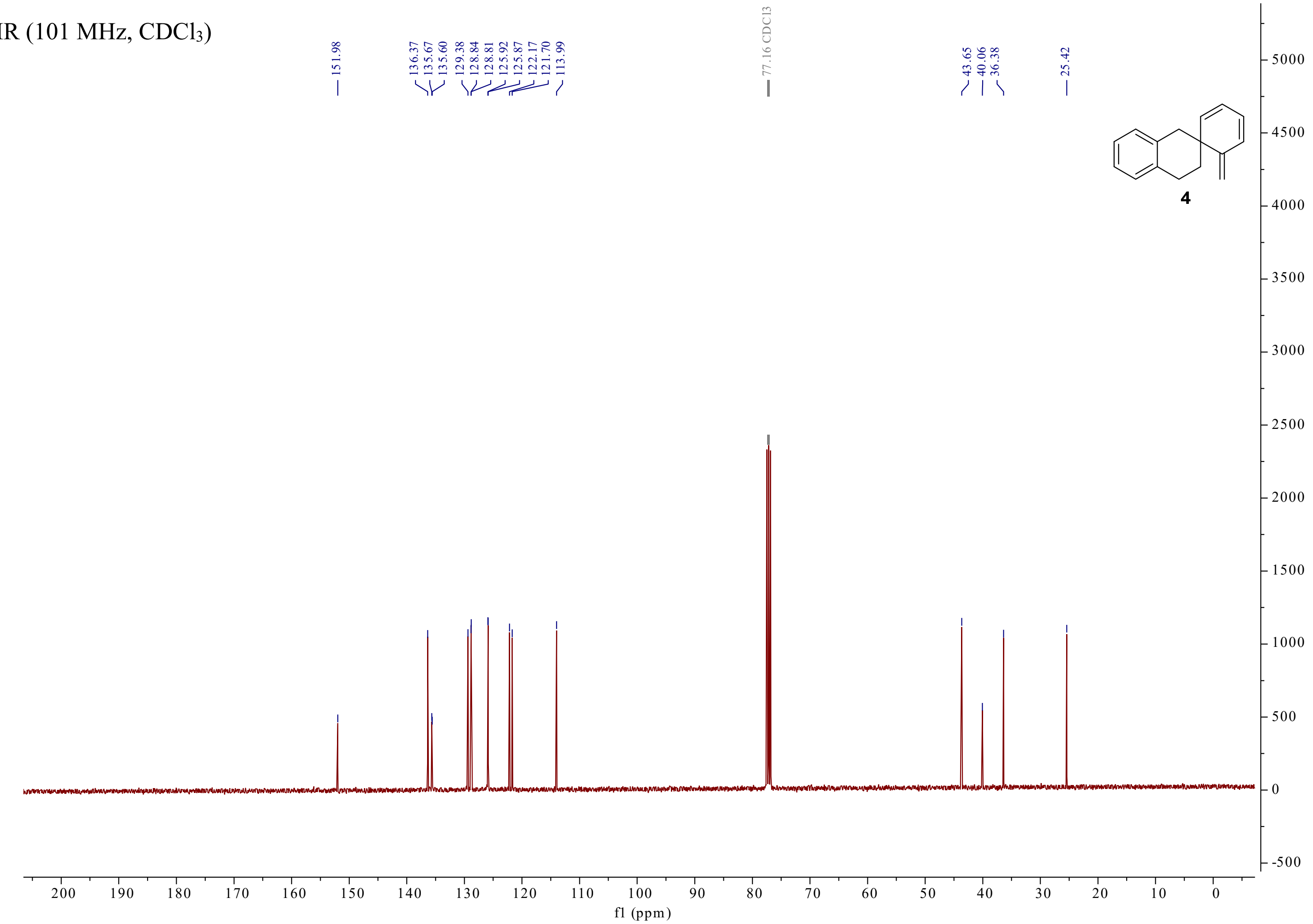

^{13}C NMR (101 MHz, CDCl$_3$)

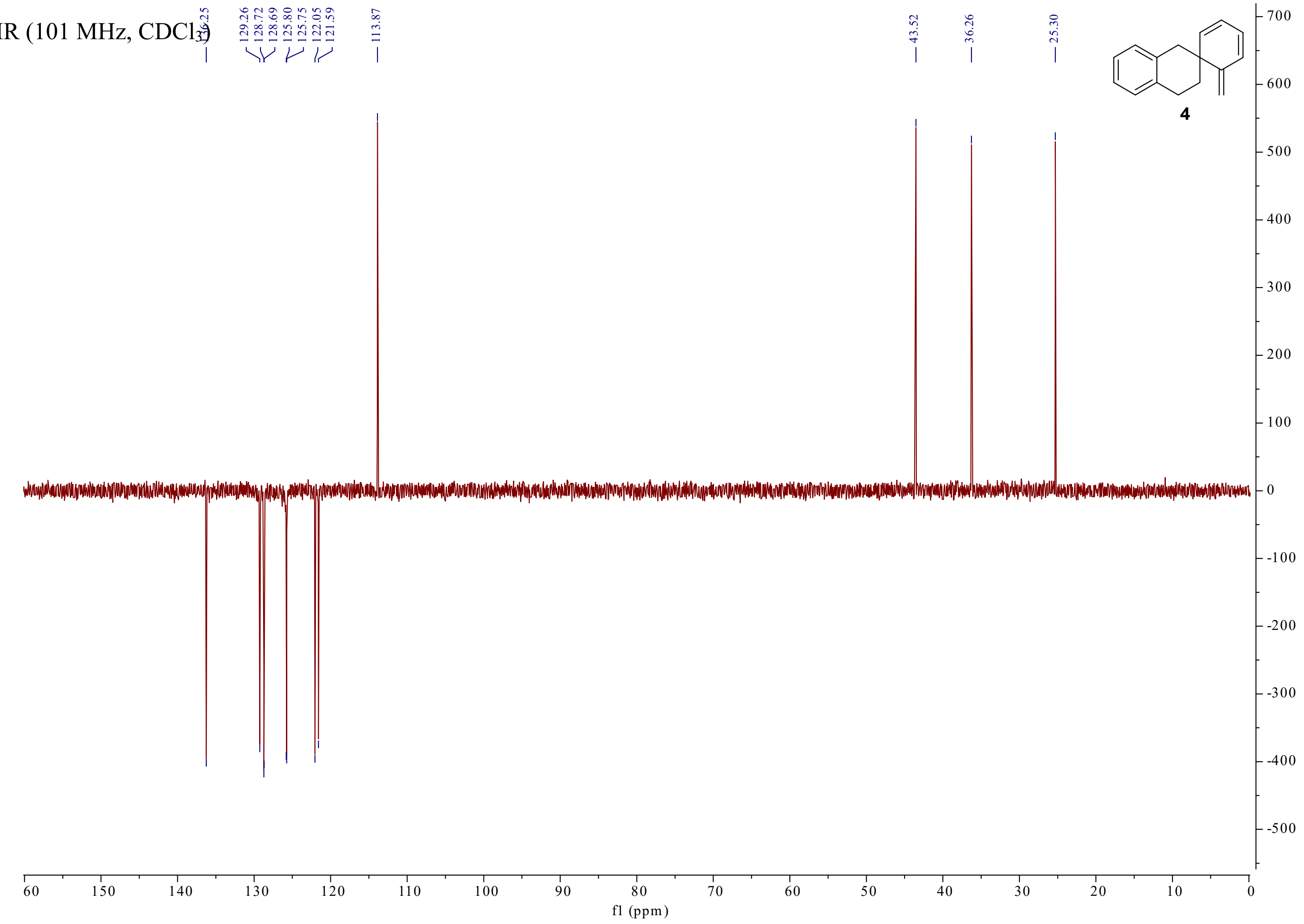

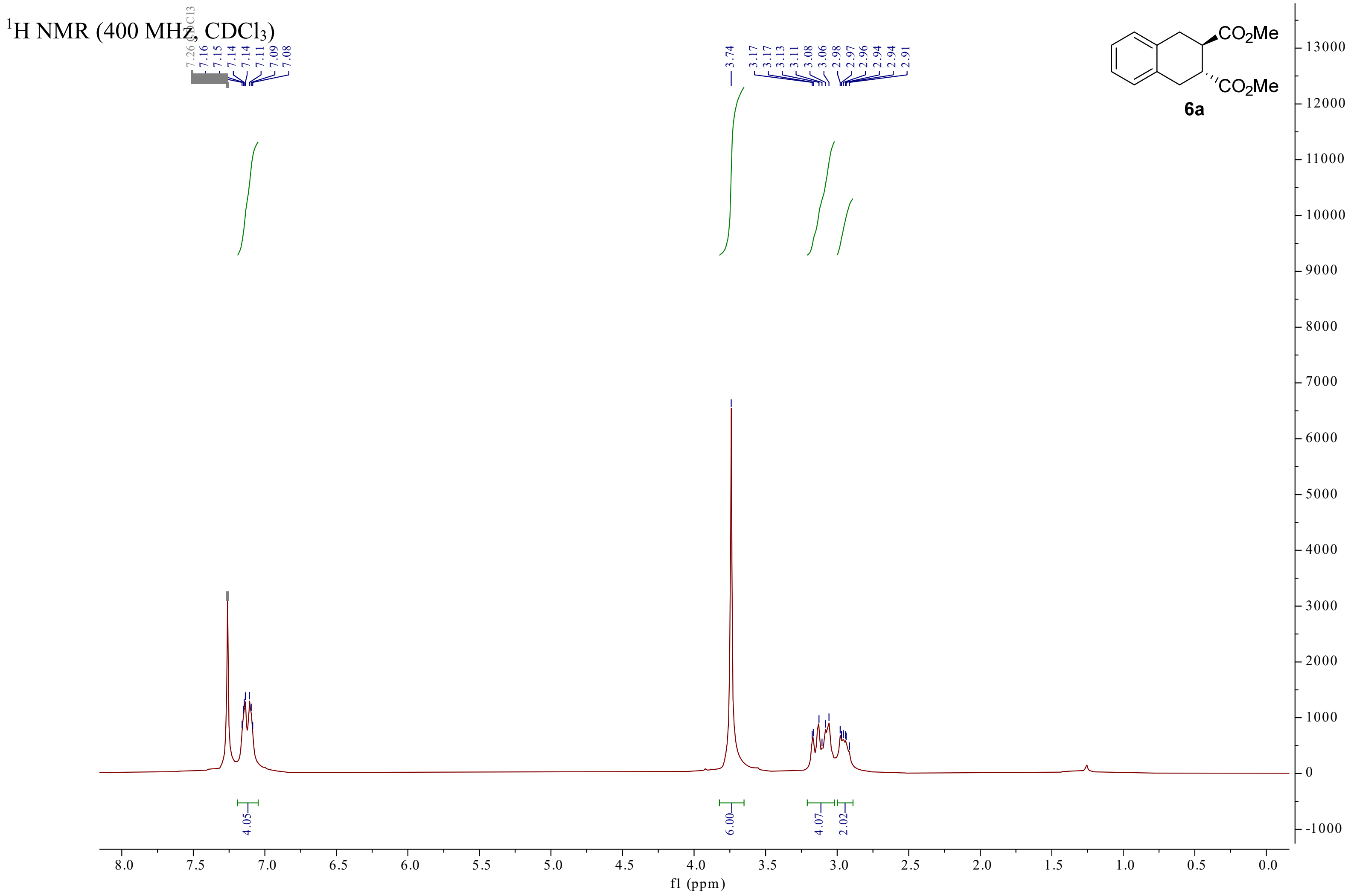

^{1}H NMR (400 MHz, CDCl$_3$)
7.26 CDCl3
7.16
7.15
7.14
7.14
7.11
7.09
7.08
3.74
3.17
3.17
3.13
3.11
3.08
3.06
2.98
2.97
2.96
2.94
2.94
2.91
CO$_2$Me
CO$_2$Me
6a
4.05
6.00
4.07
2.02
8.0 7.5 7.0 6.5 6.0 5.5 5.0 4.5 4.0 3.5 3.0 2.5 2.0 1.5 1.0 0.5 0.0
f1 (ppm)
13000 12000 11000 10000 9000 8000 7000 6000 5000 4000 3000 2000 1000 0 -1000

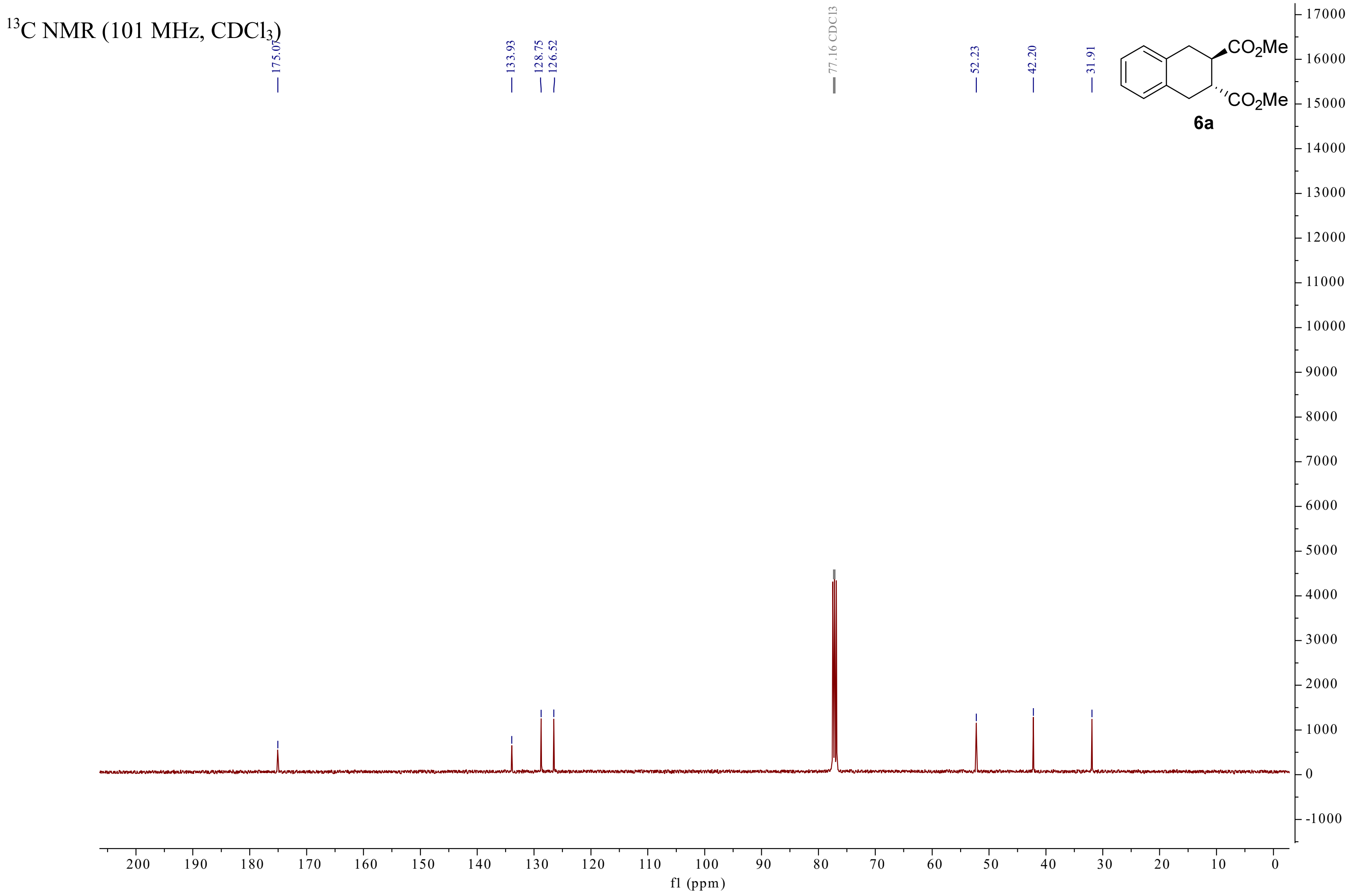

^{13}C NMR (101 MHz, $CDCl_3$)
175.07
133.93
128.75
126.52
77.16 CDCl3
52.23
42.20
31.91
CO2Me
CO2Me
6a
f1 (ppm)

^{1}H NMR (400 MHz, CDCl$_3$)

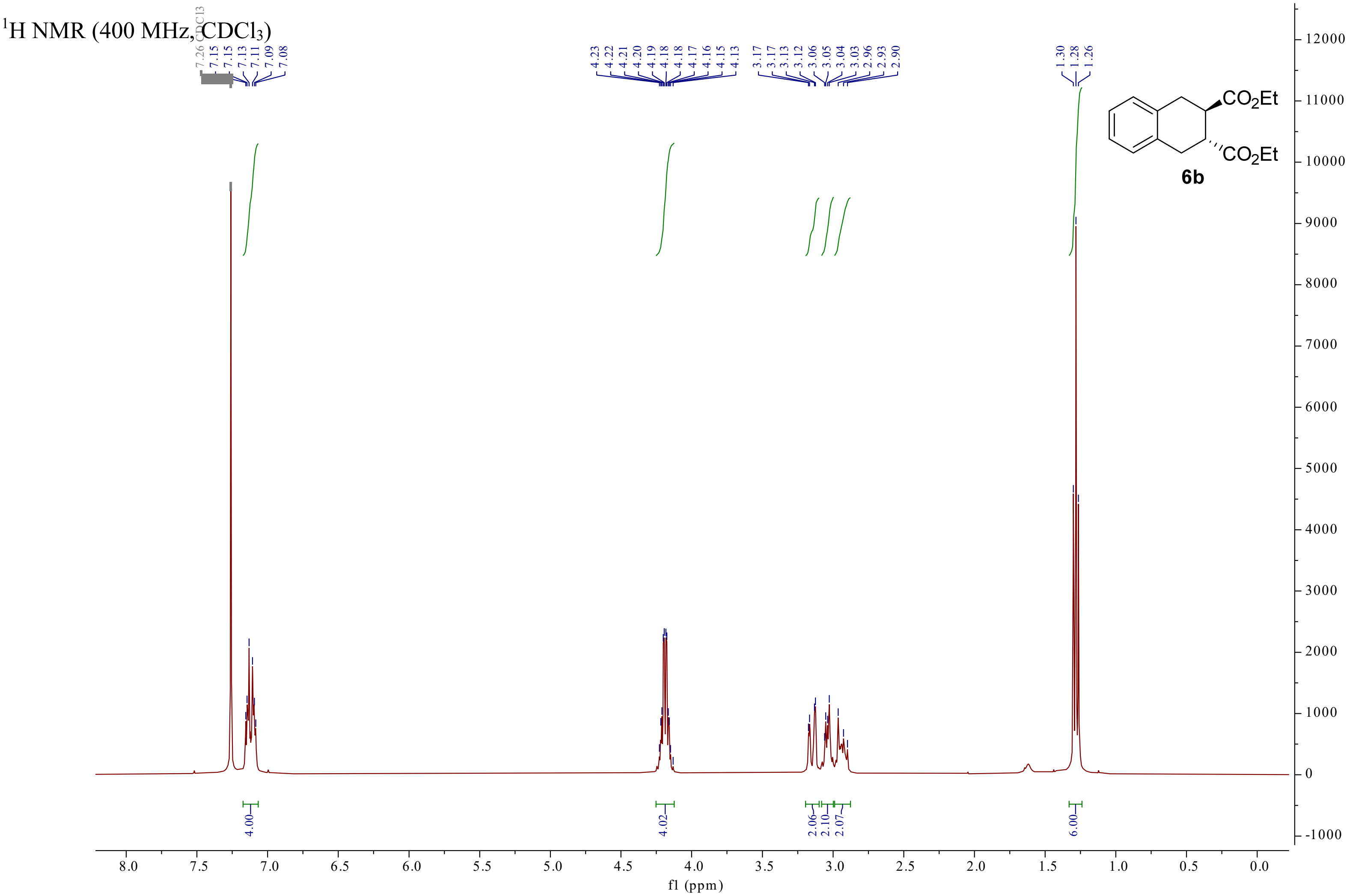

^{13}C NMR (101 MHz, $CDCl_3$)

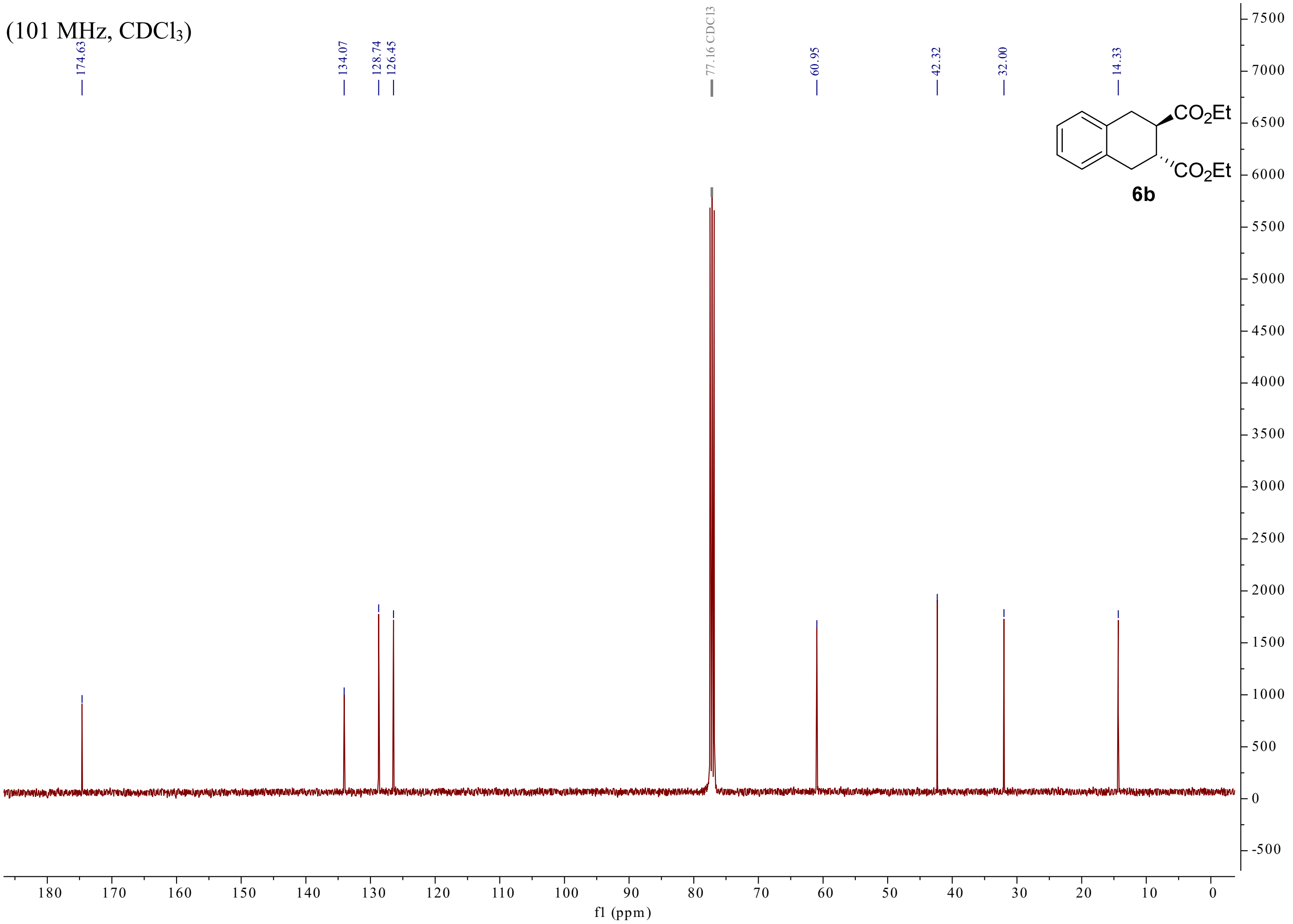

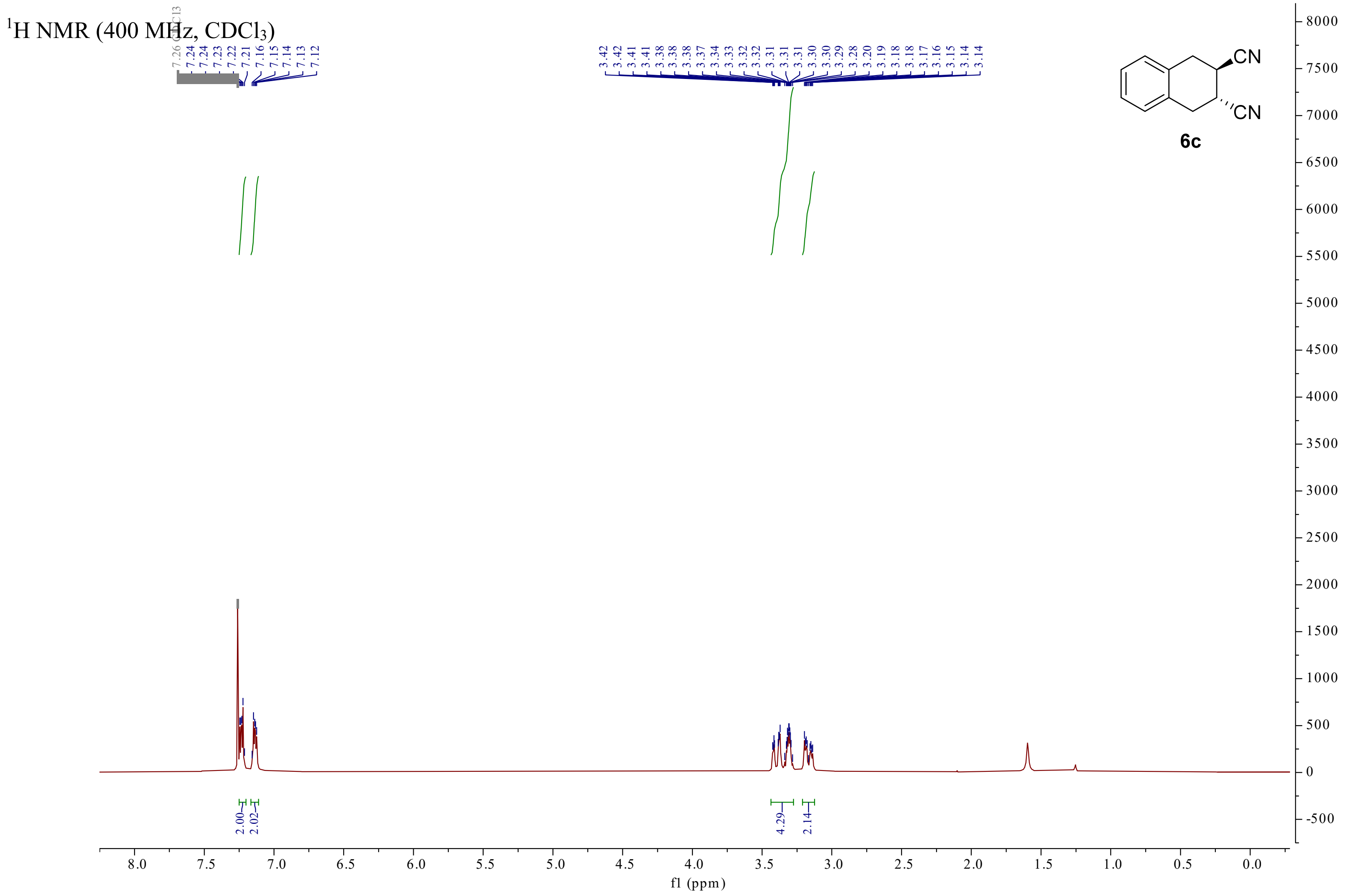

^{1}H NMR (400 MHz, CDCl3)
6c
CN
CN
f1 (ppm)

^{13}C NMR (101 MHz, $CDCl_3$)

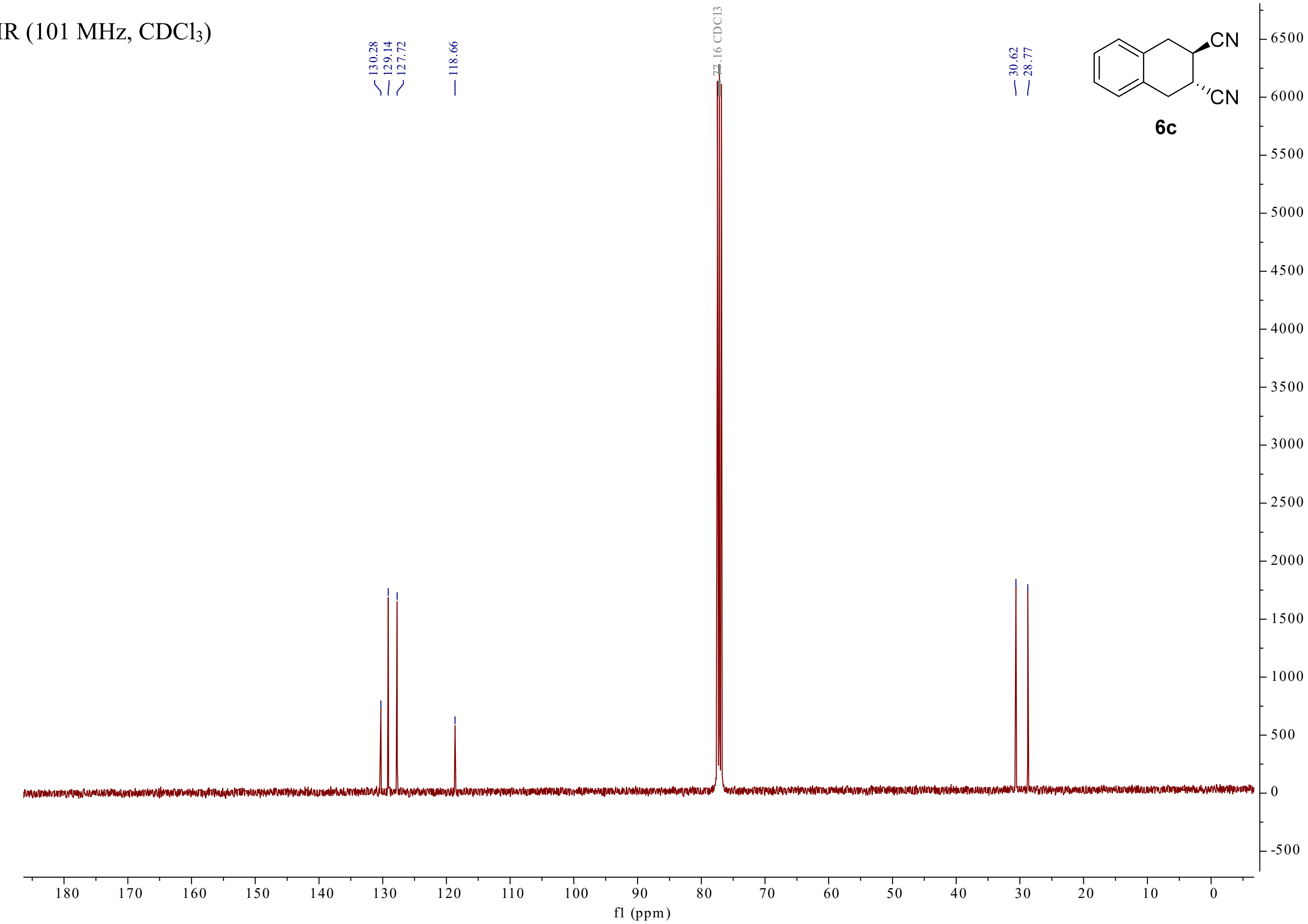

^1H NMR (400 MHz, $CDCl_3$)

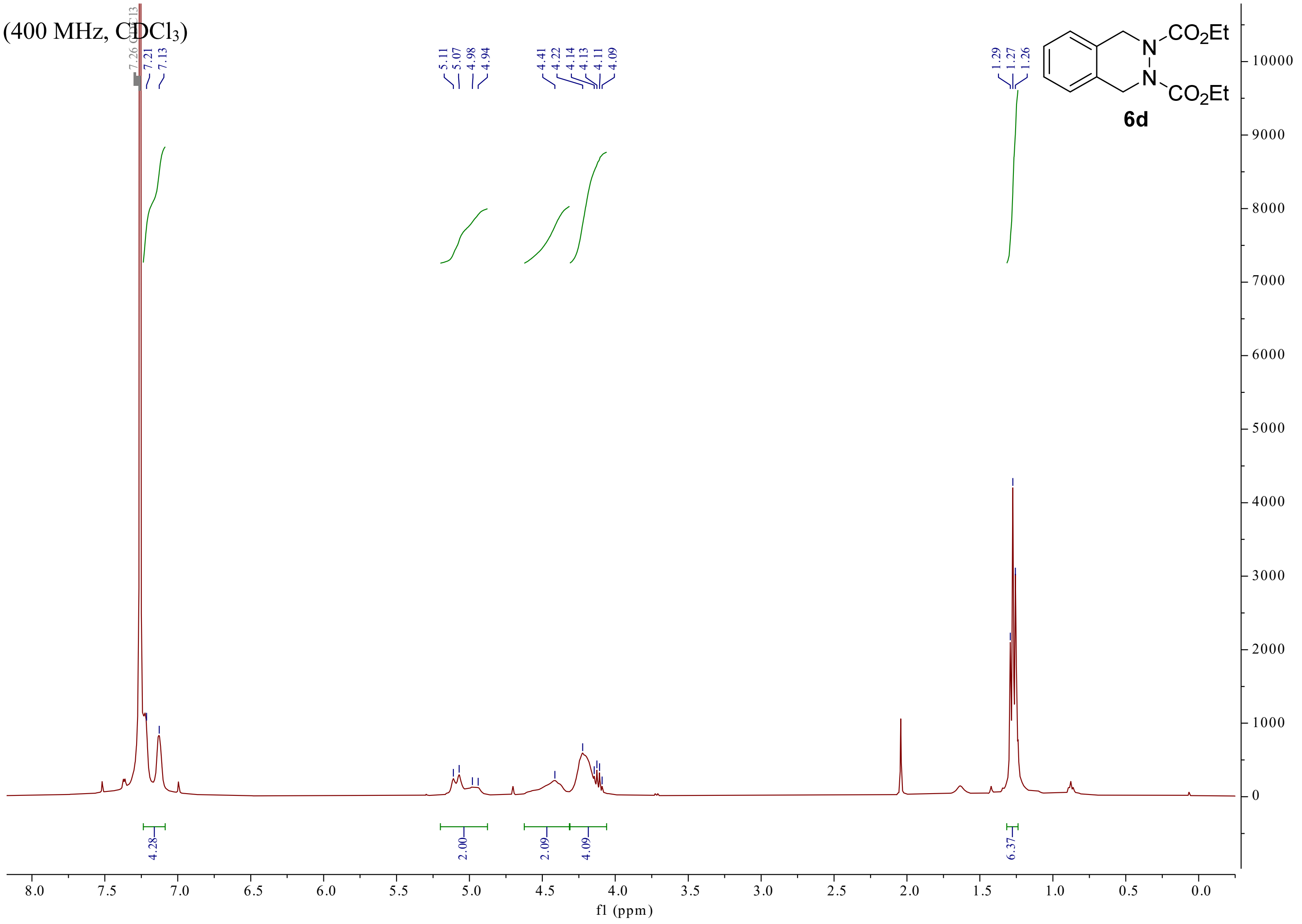

6d

^{13}C NMR (101 MHz, $CDCl_3$)

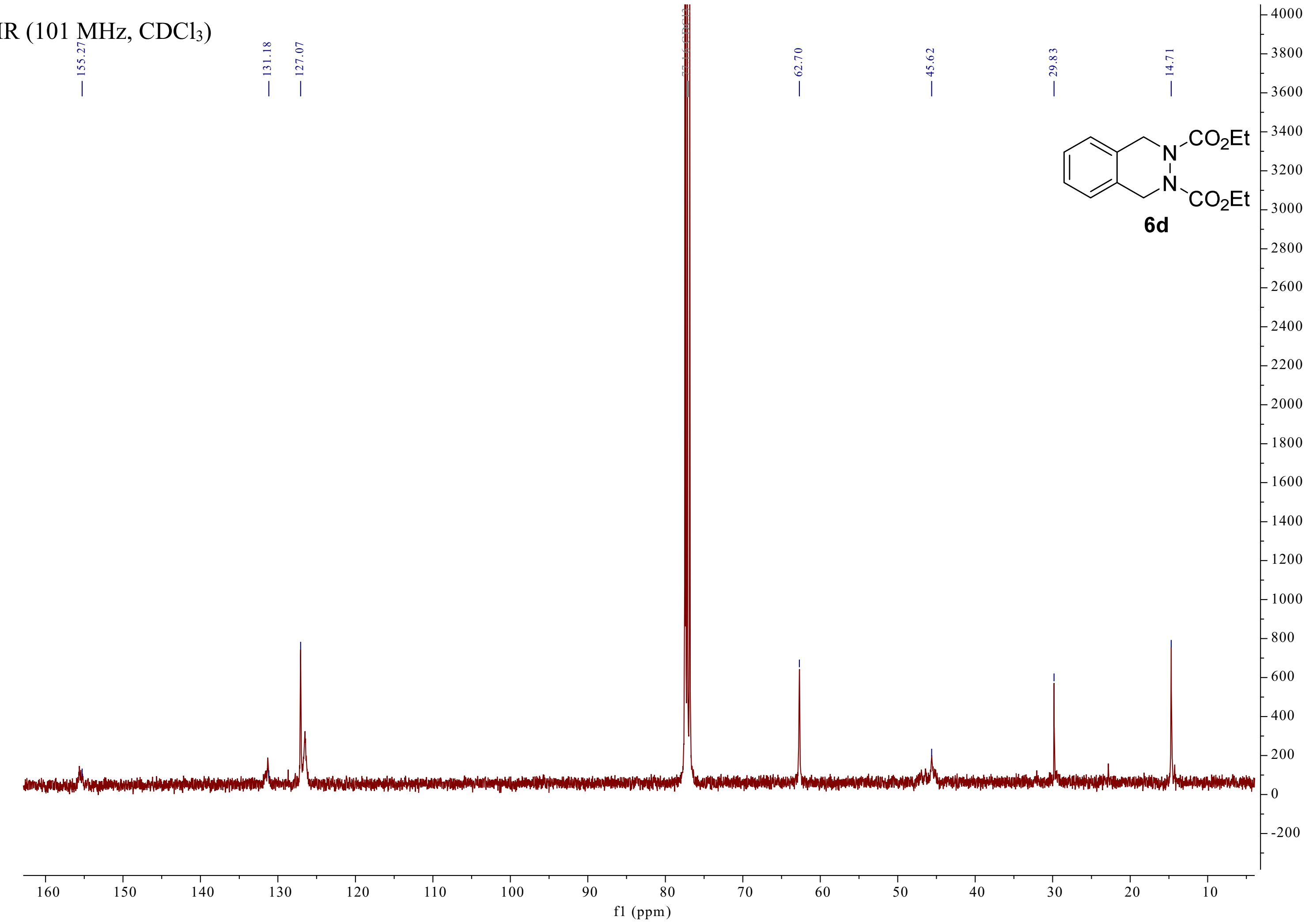

^{1}H NMR (600 MHz, $CDCl_3$)

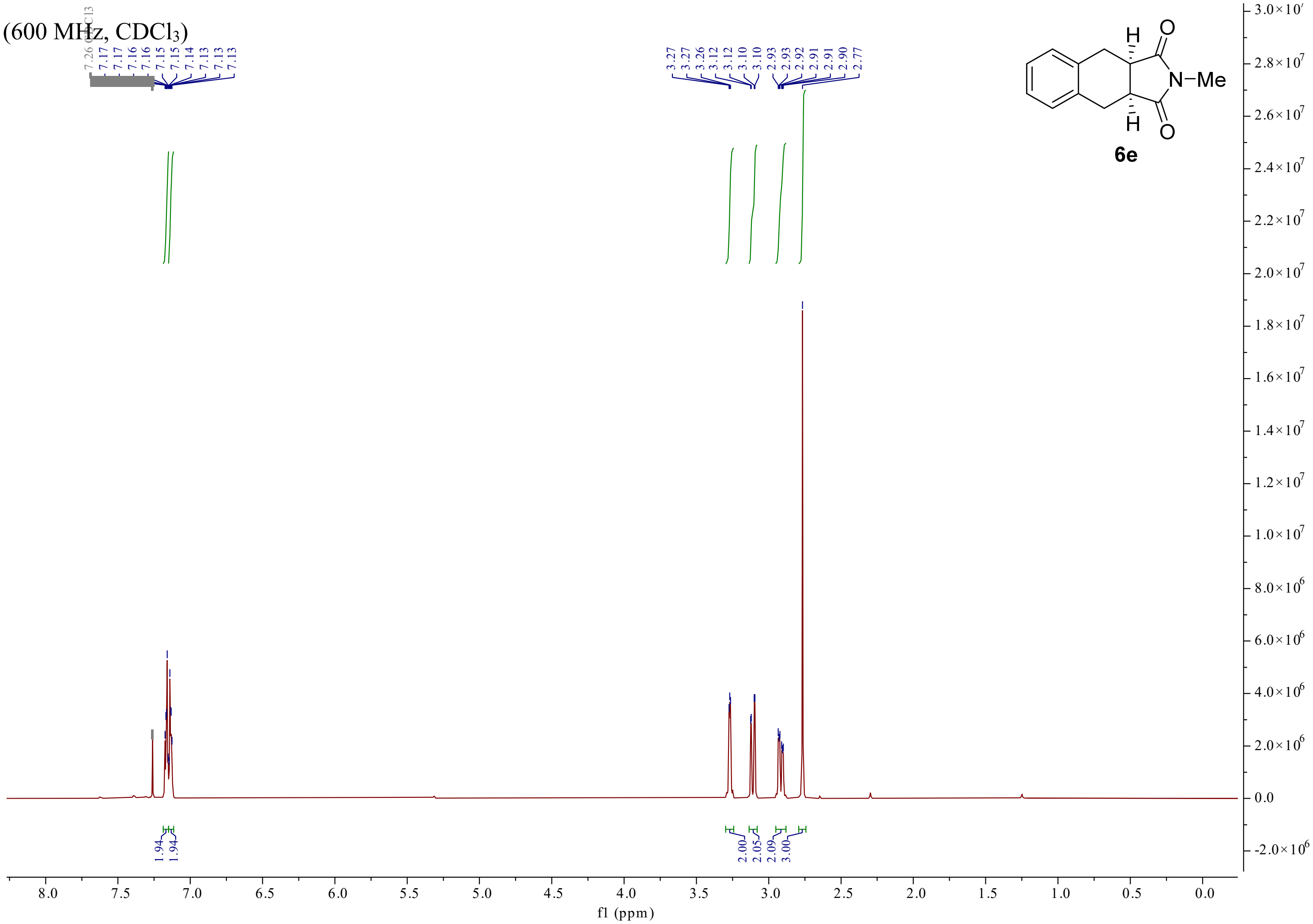

^{13}C NMR (101 MHz, $CDCl_3$)

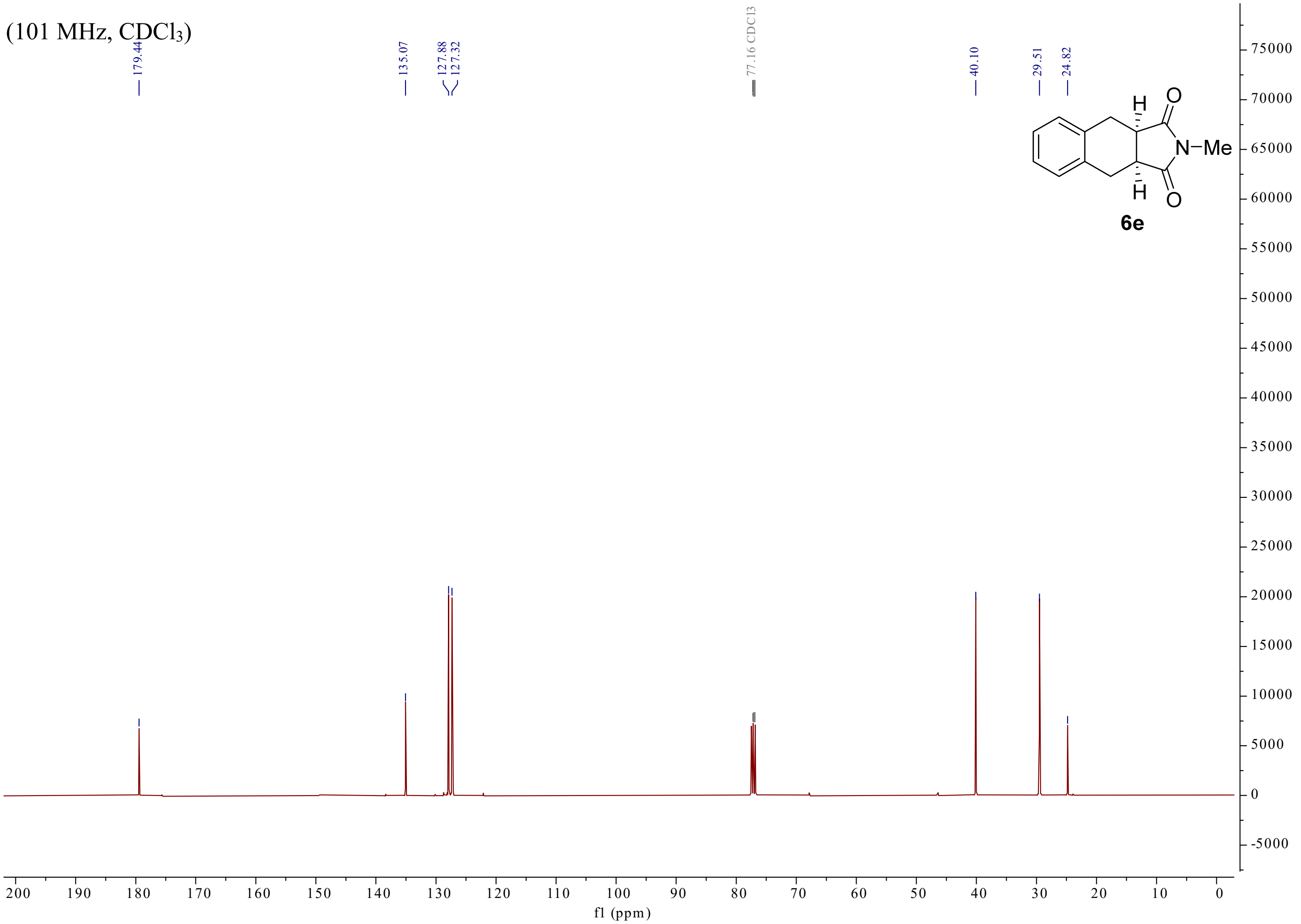

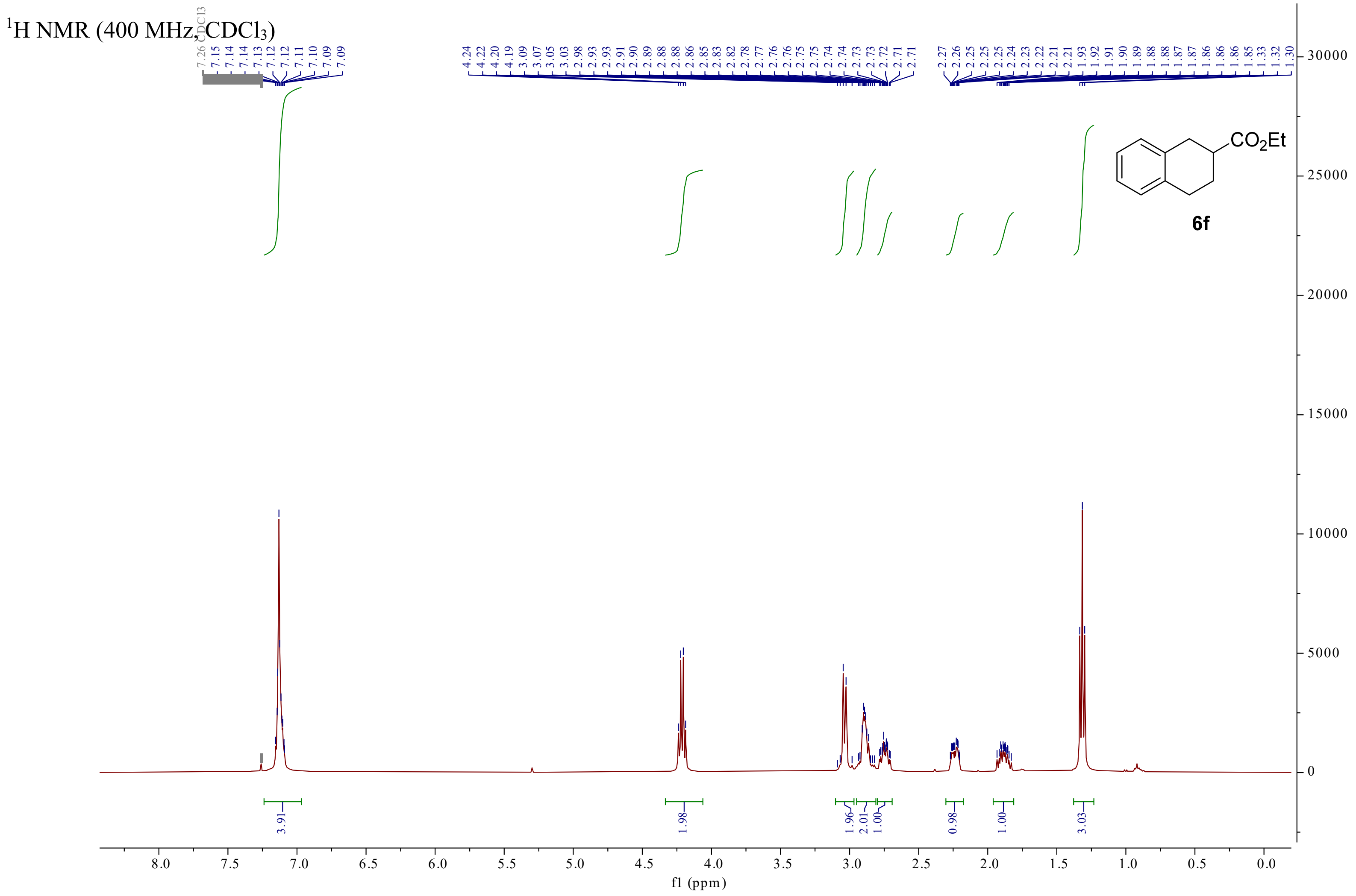

1H NMR (400 MHz, CDCl3)
CO2Et
6f
f1 (ppm)

^{13}C NMR (101 MHz, $CDCl_3$)

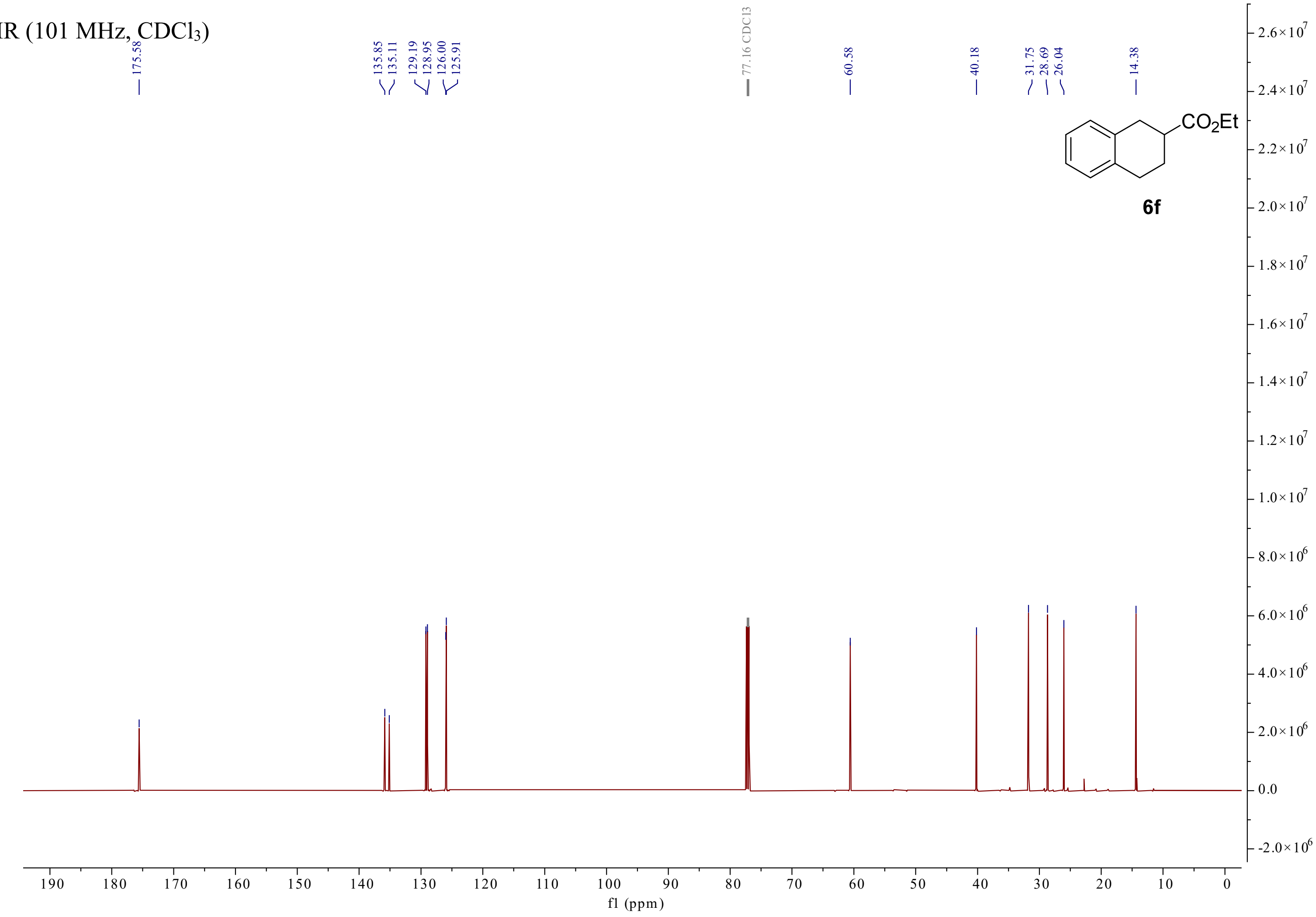

^{1}H NMR (400 MHz, CDCl$_3$)

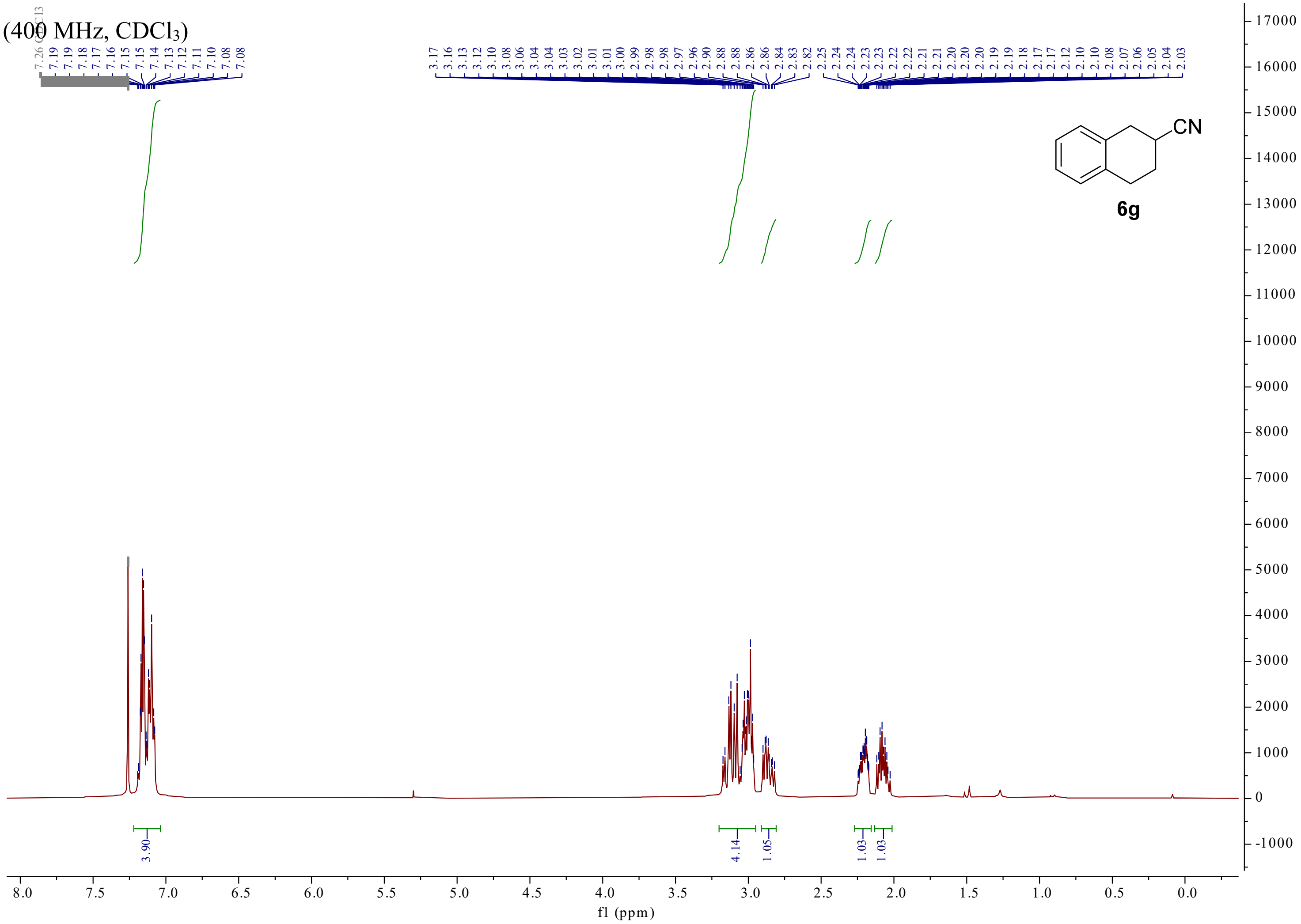

^{13}C NMR (101 MHz, $CDCl_3$)

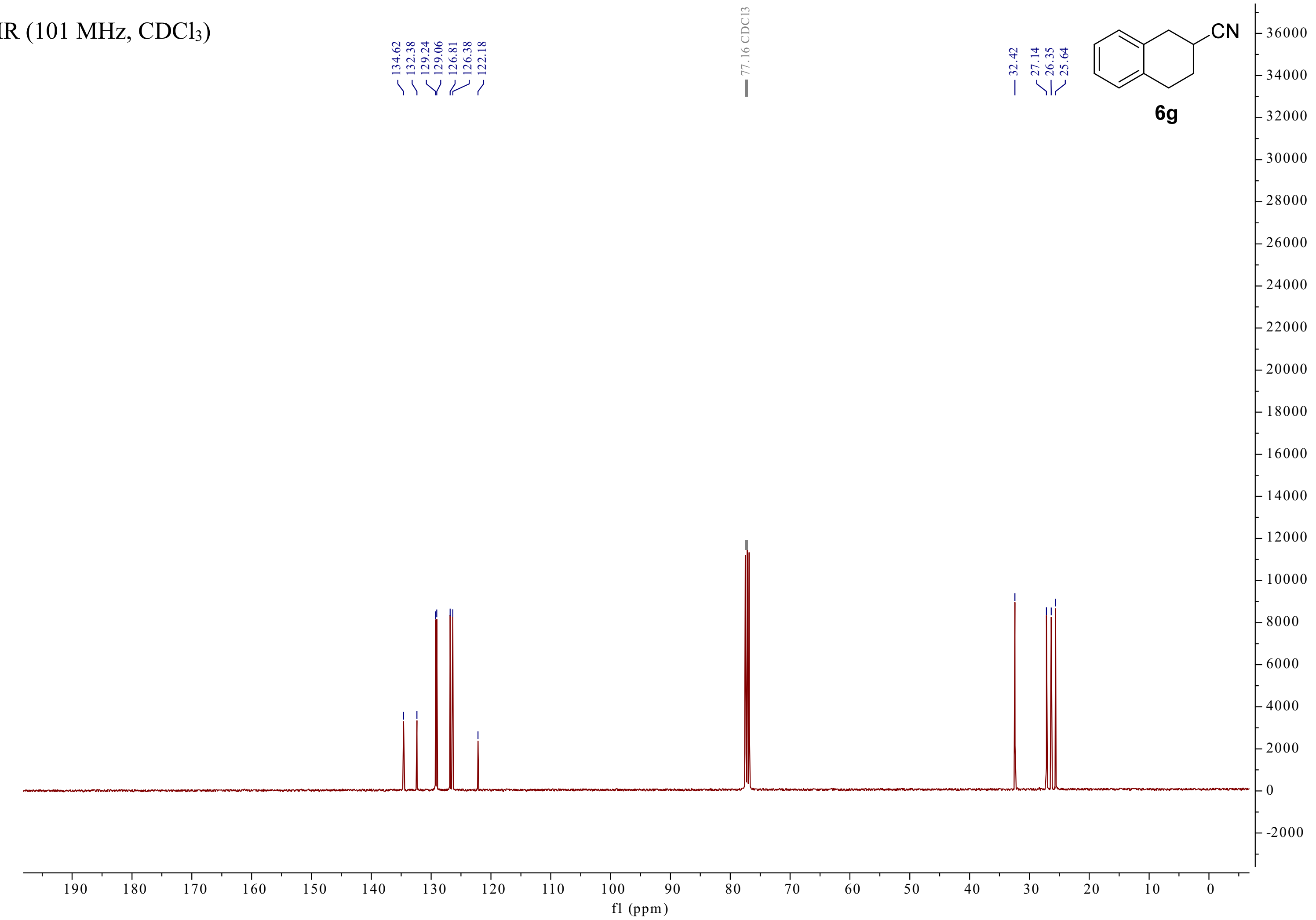

^{1}H NMR (400 MHz, $CDCl_3$)

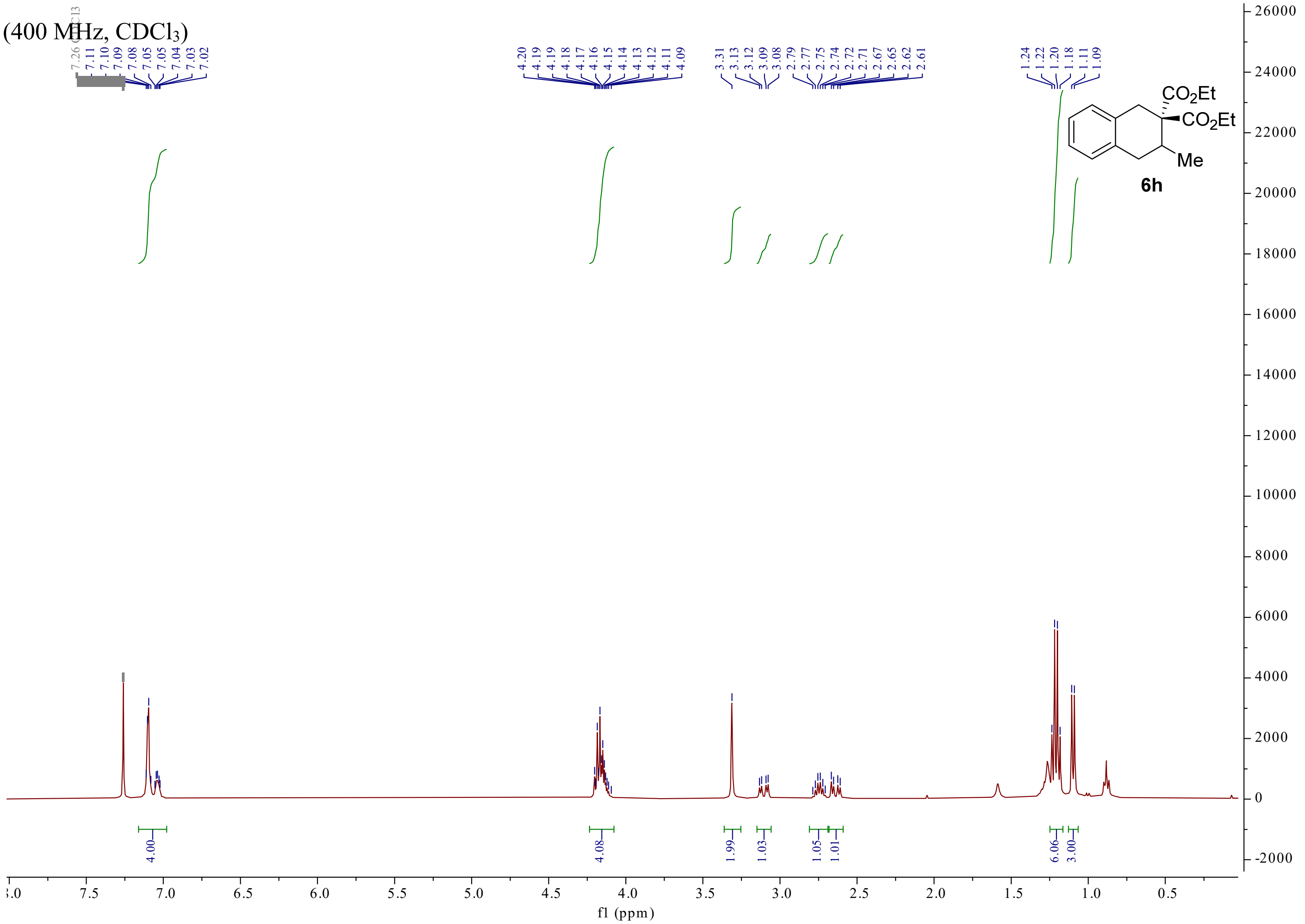

^{13}C NMR (101 MHz, $CDCl_3$)

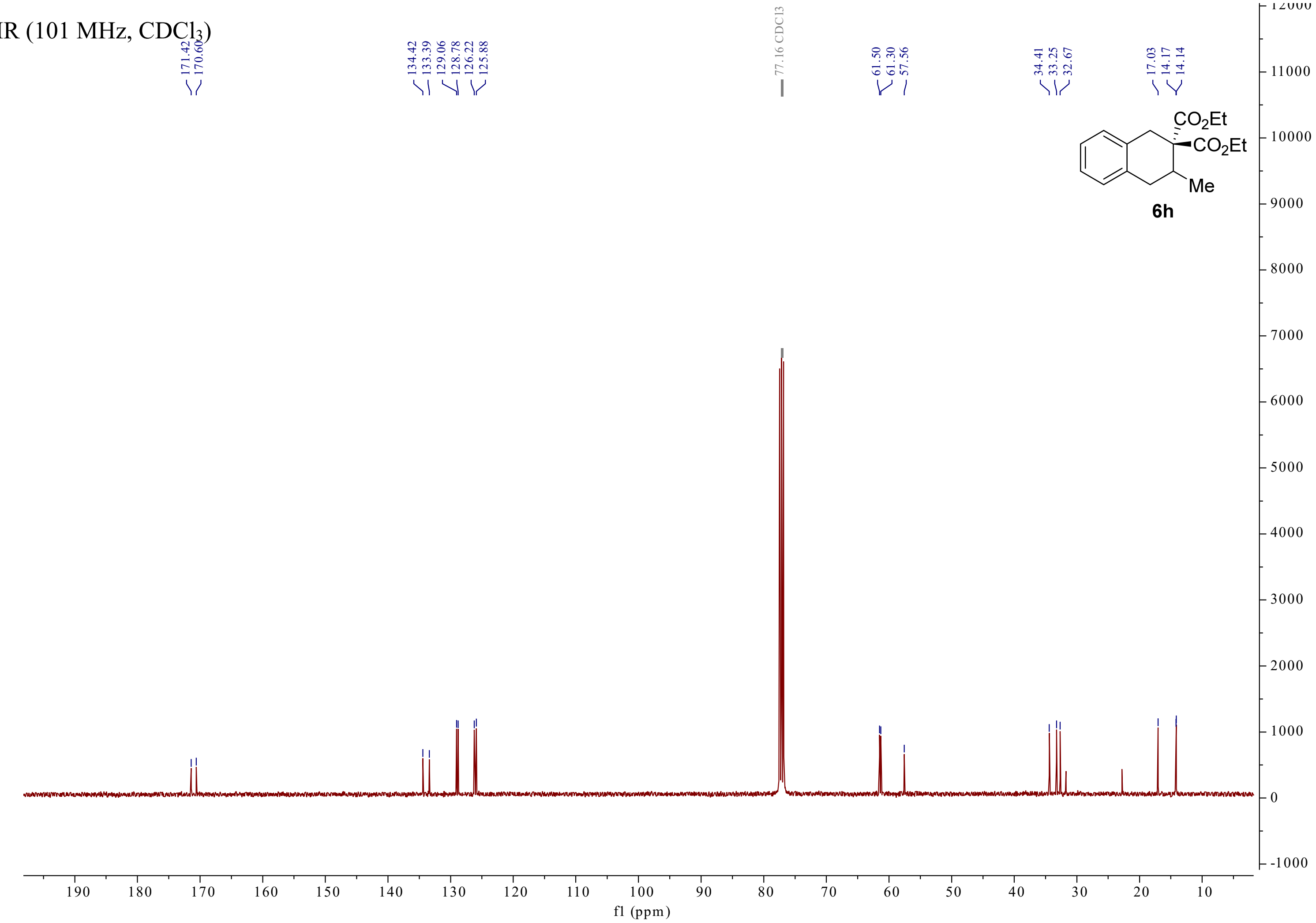

6h

^{1}H NMR (400 MHz, CDCl$_3$)

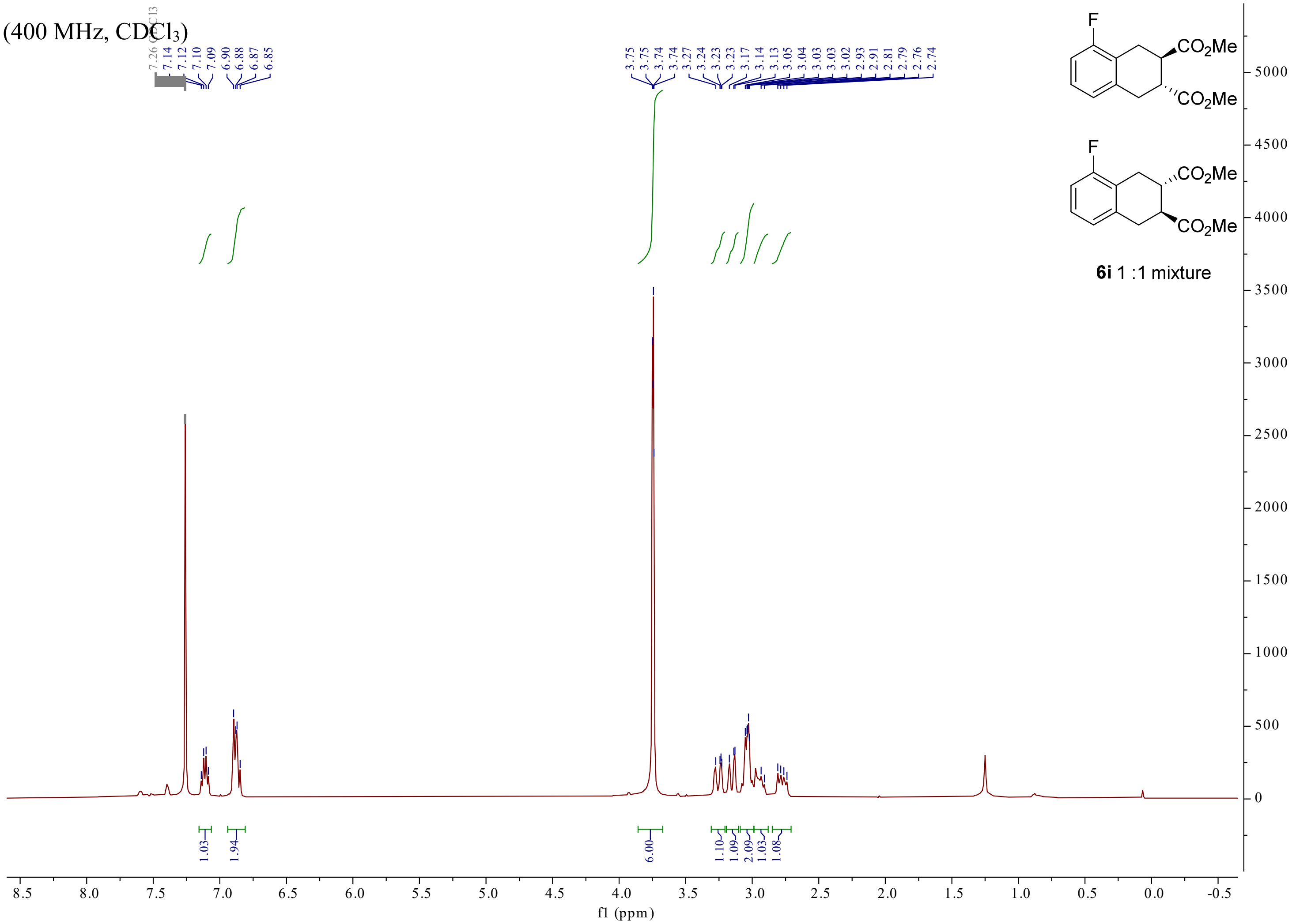

6i 1 :1 mixture

^{13}C NMR (101 MHz, $CDCl_3$)

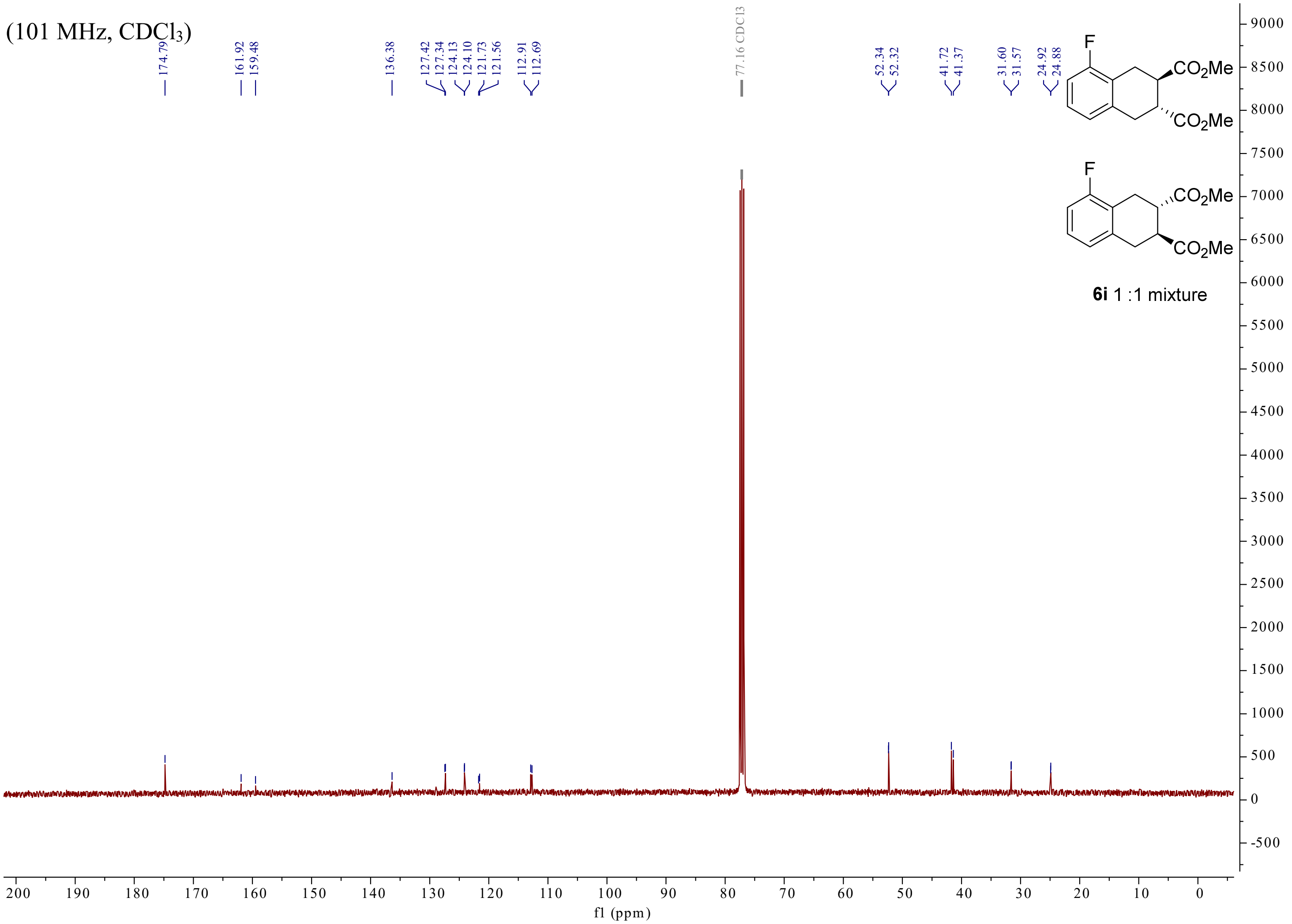

^{19}F NMR (376 MHz, $CDCl_3$)

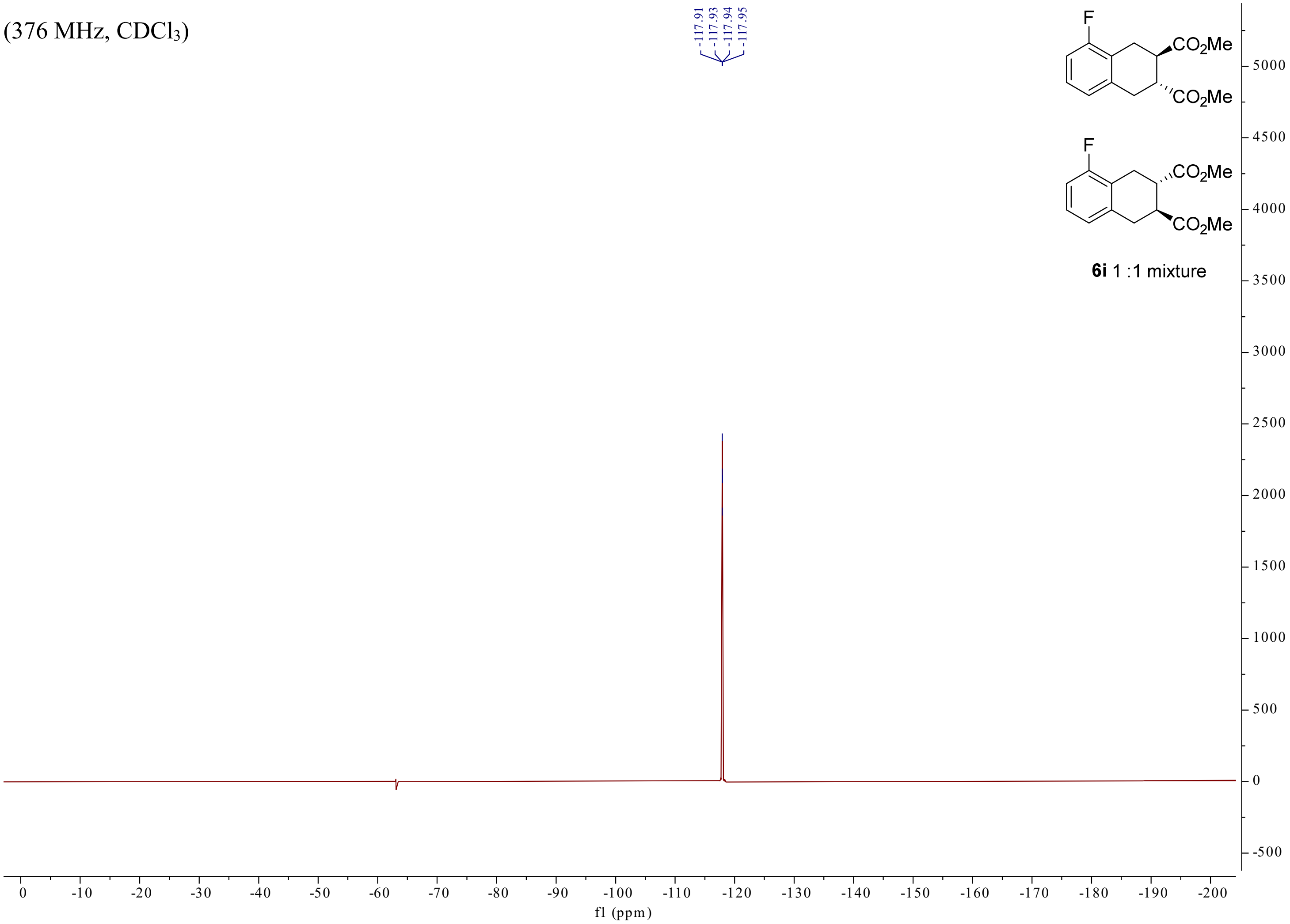

6i 1 :1 mixture

^{1}H NMR (400 MHz, $CDCl_3$)

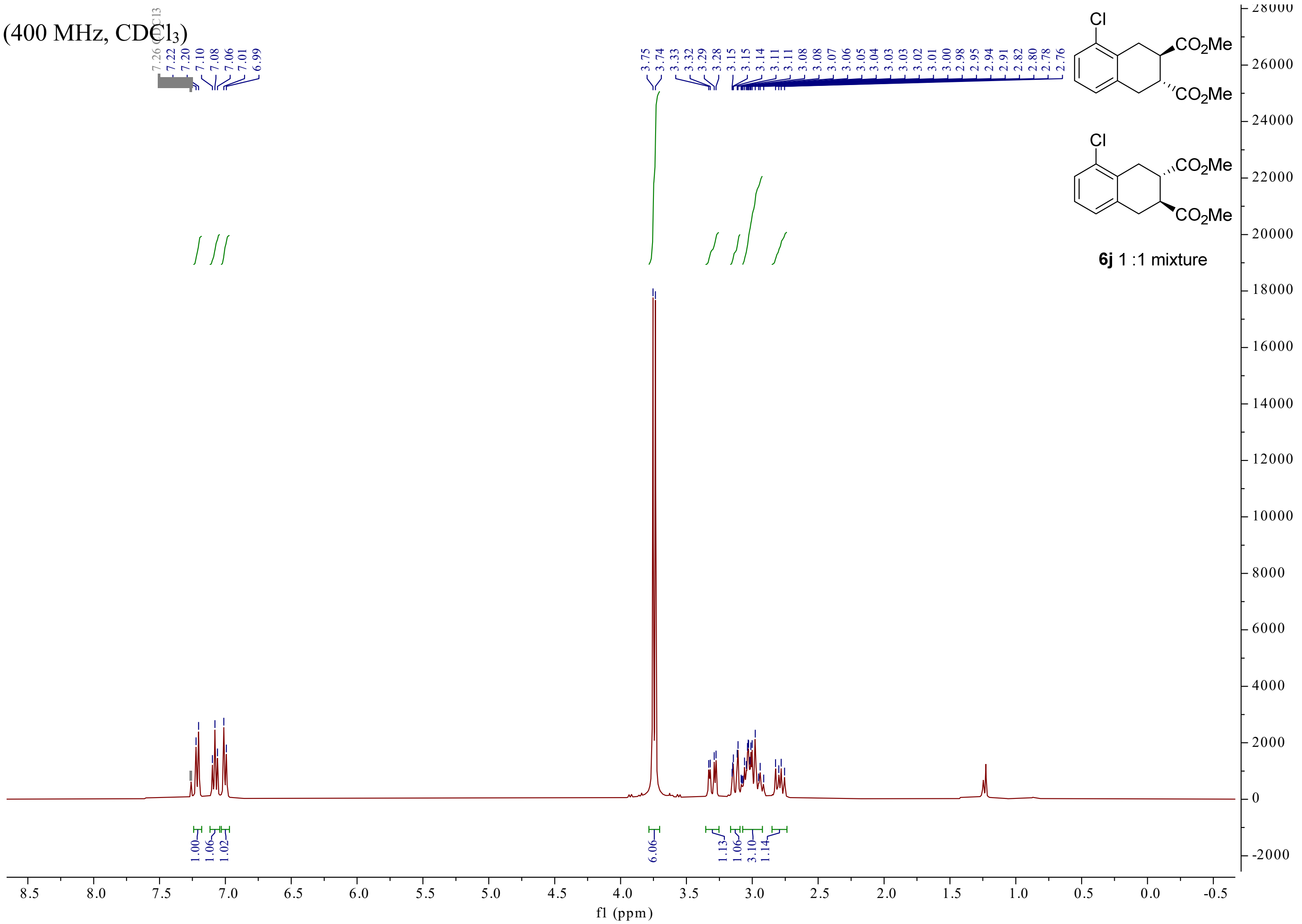

^{13}C NMR (101 MHz, $CDCl_3$)

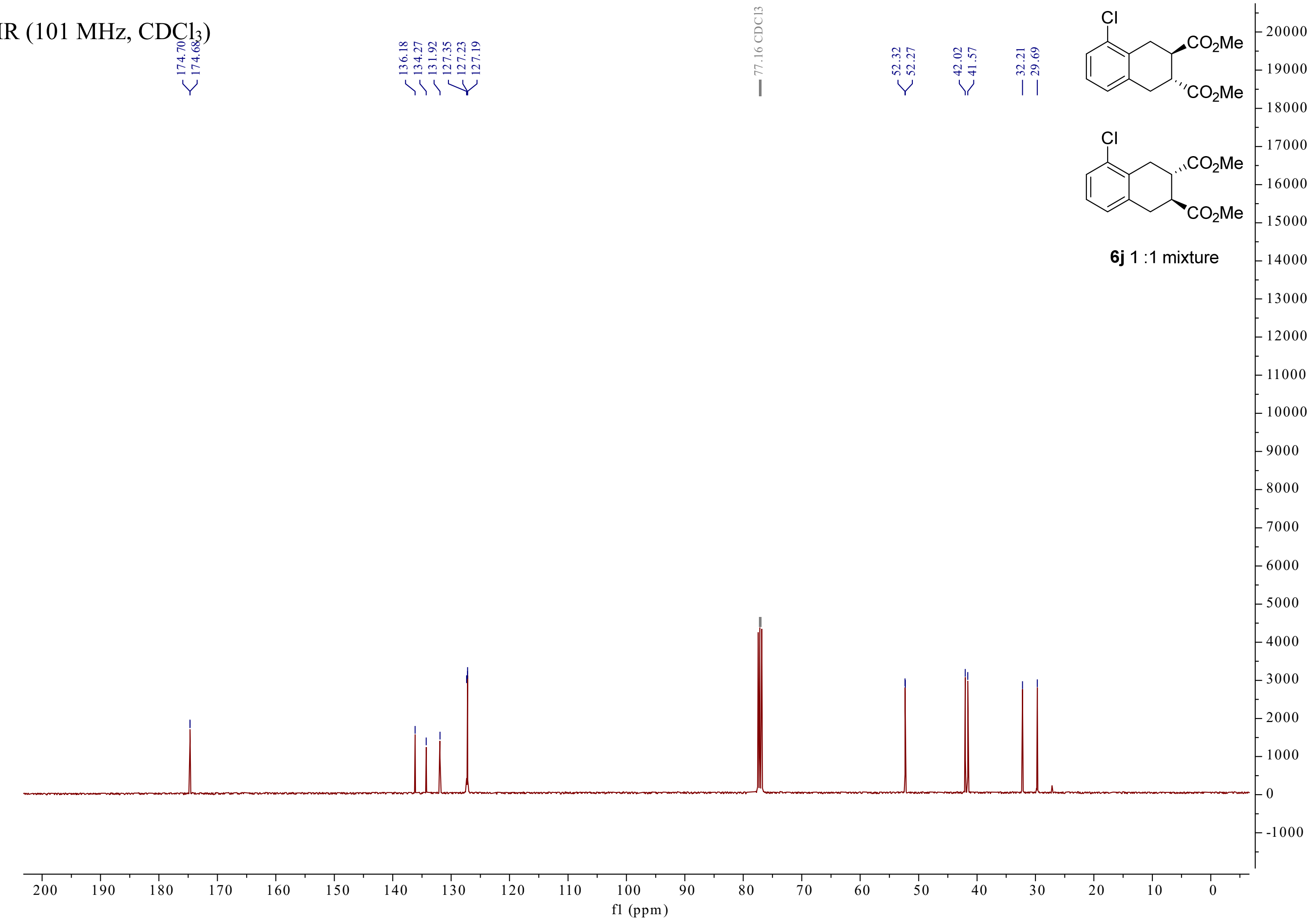

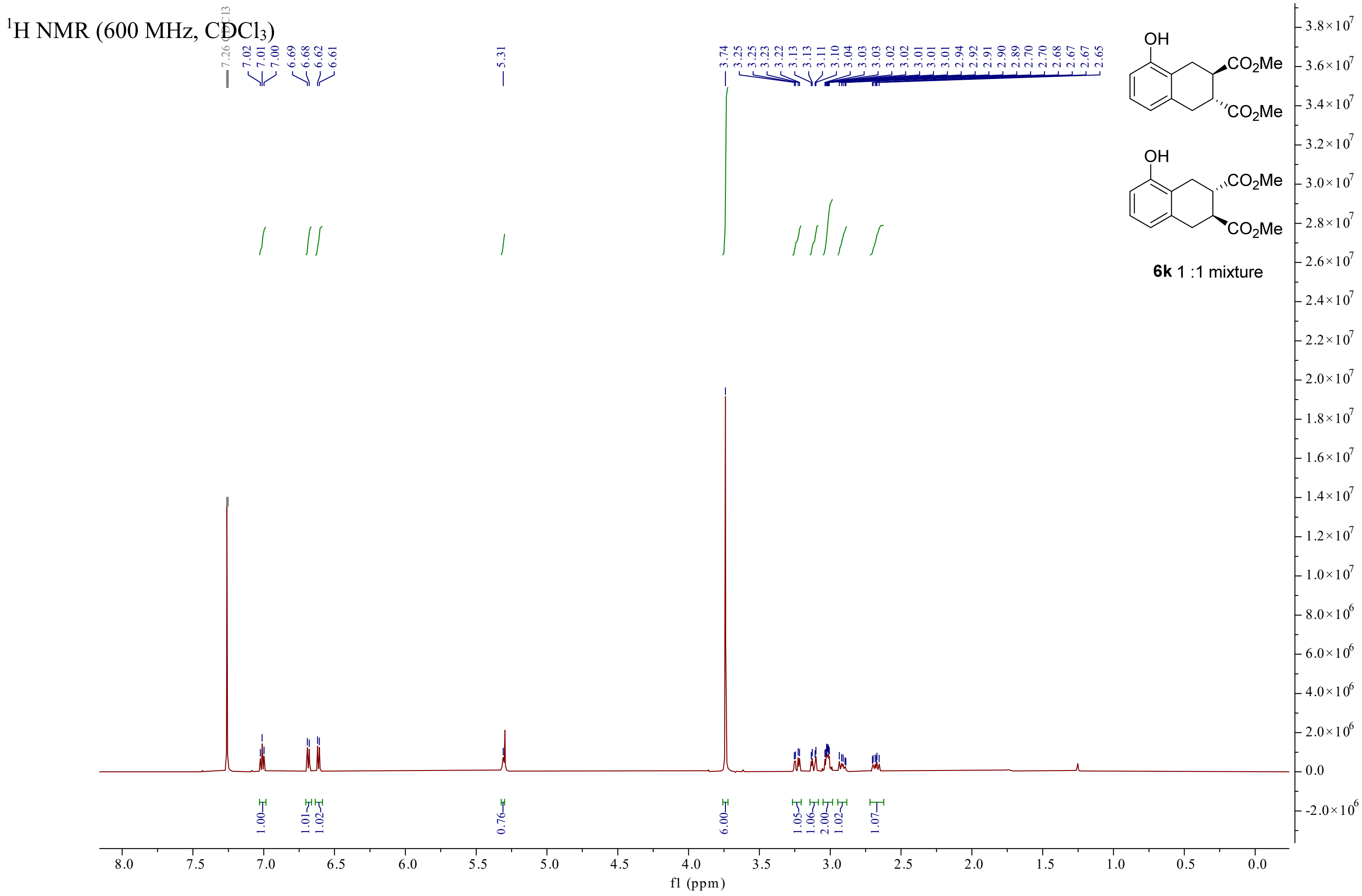

^{1}H NMR (600 MHz, CDCl$_3$)
7.26 CDCl3
7.02
7.01
7.00
6.69
6.68
6.62
6.61
5.31
3.74
3.25
3.25
3.23
3.22
3.13
3.13
3.11
3.10
3.04
3.03
3.03
3.02
3.01
3.01
3.01
2.94
2.92
2.91
2.90
2.89
2.70
2.68
2.67
2.67
2.65
OH
CO_2Me
CO_2Me
OH
CO_2Me
CO_2Me
6k 1 :1 mixture
1.00
1.01
1.02
0.76
6.00
1.05
1.06
2.00
1.02
1.07
8.0
7.5
7.0
6.5
6.0
5.5
5.0
4.5
4.0
3.5
3.0
2.5
2.0
1.5
1.0
0.5
0.0
f1 (ppm)

^{13}C NMR (101 MHz, $CDCl_3$)

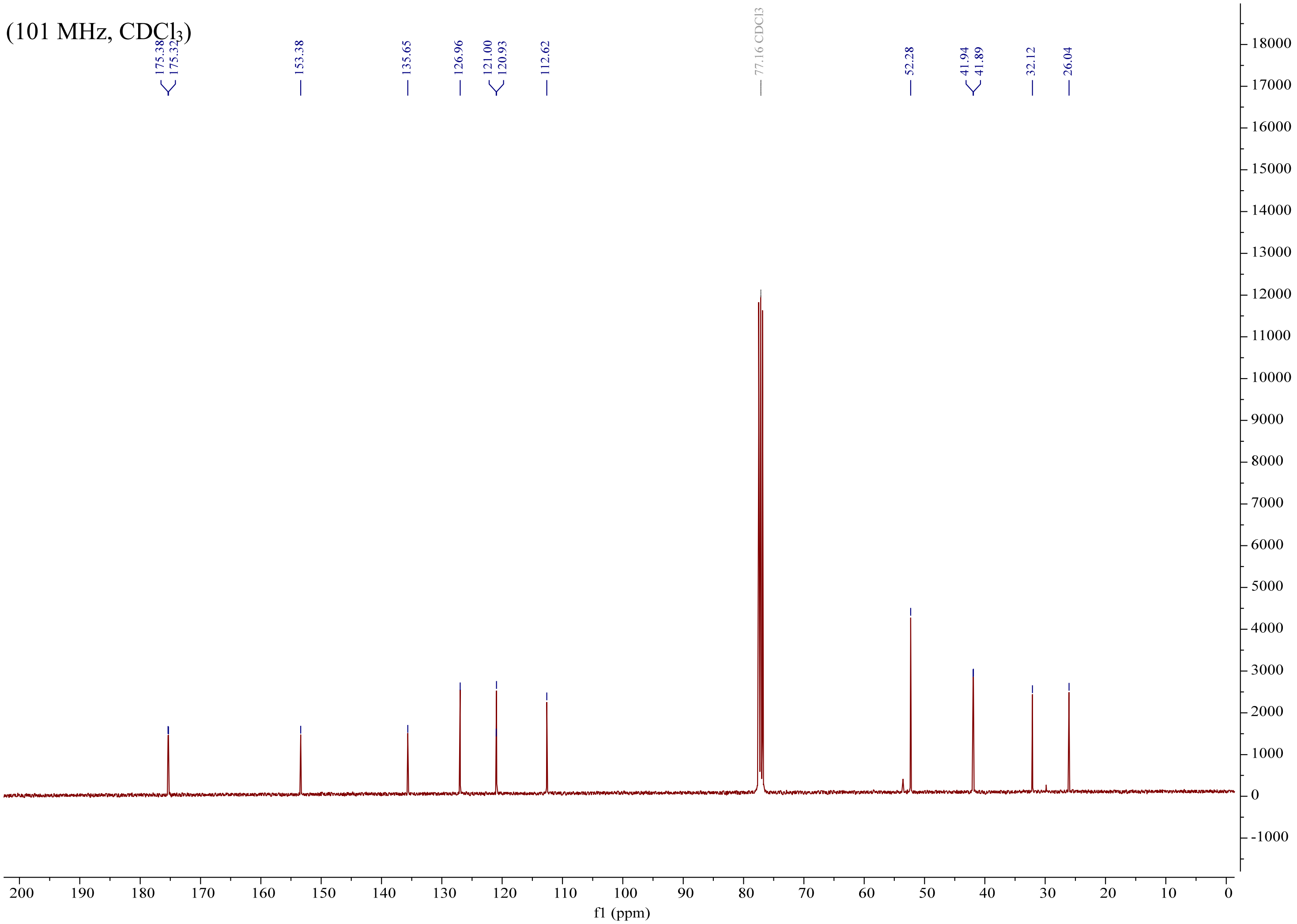

^{1}H NMR (400 MHz, $CDCl_3$)

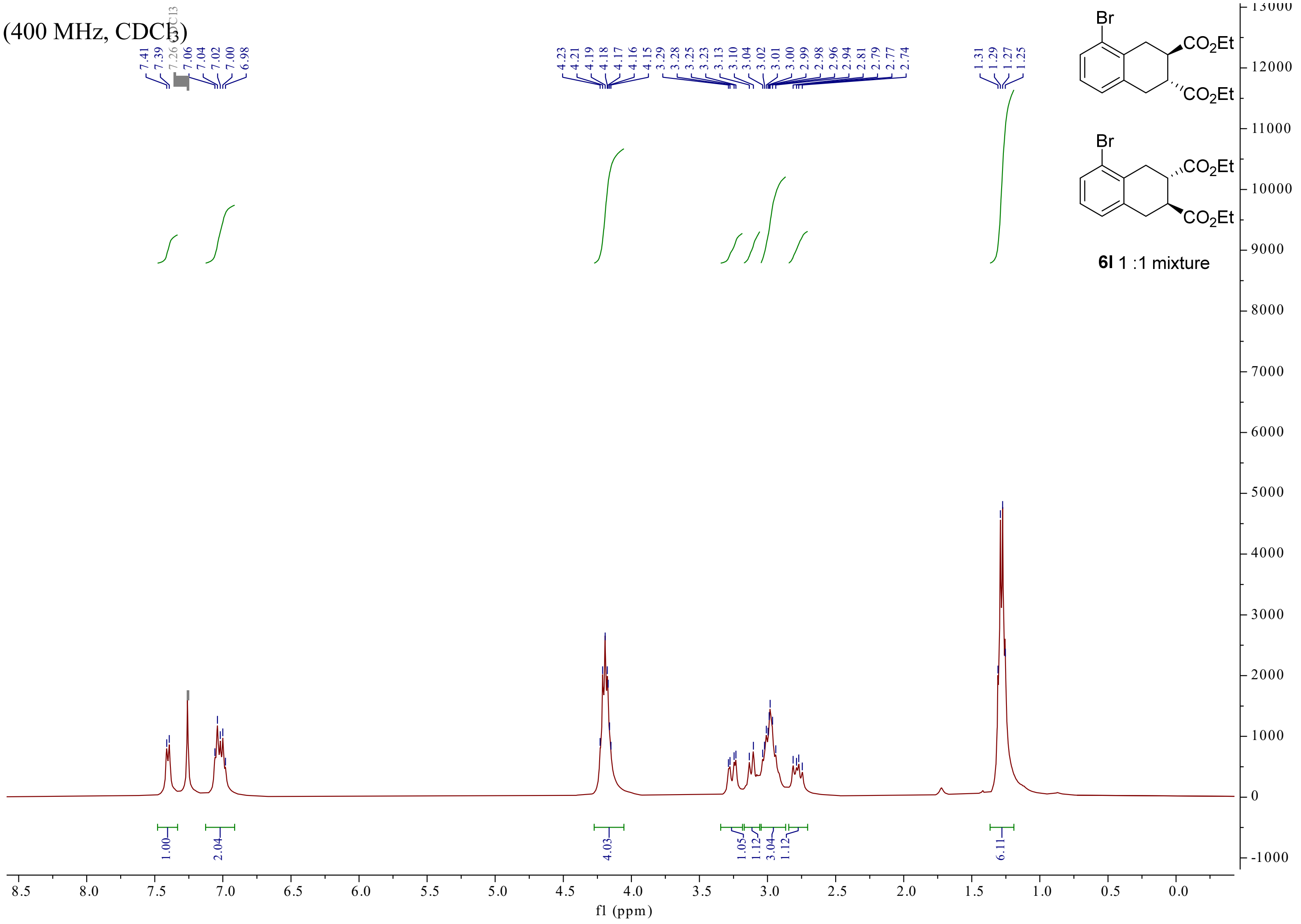

^{13}C NMR (101 MHz, CDCl3)

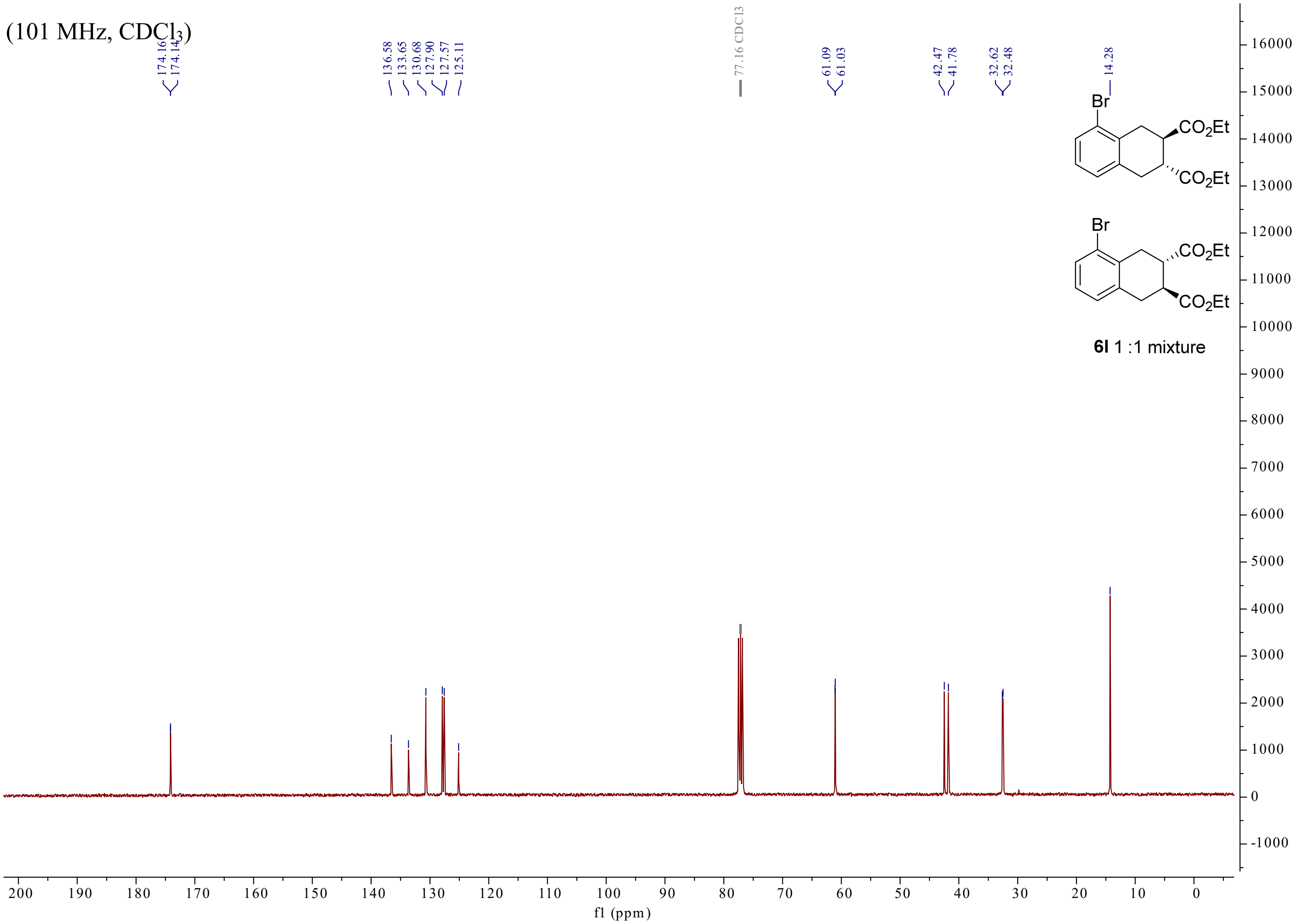

^{1}H NMR (400 MHz, $CDCl_3$)

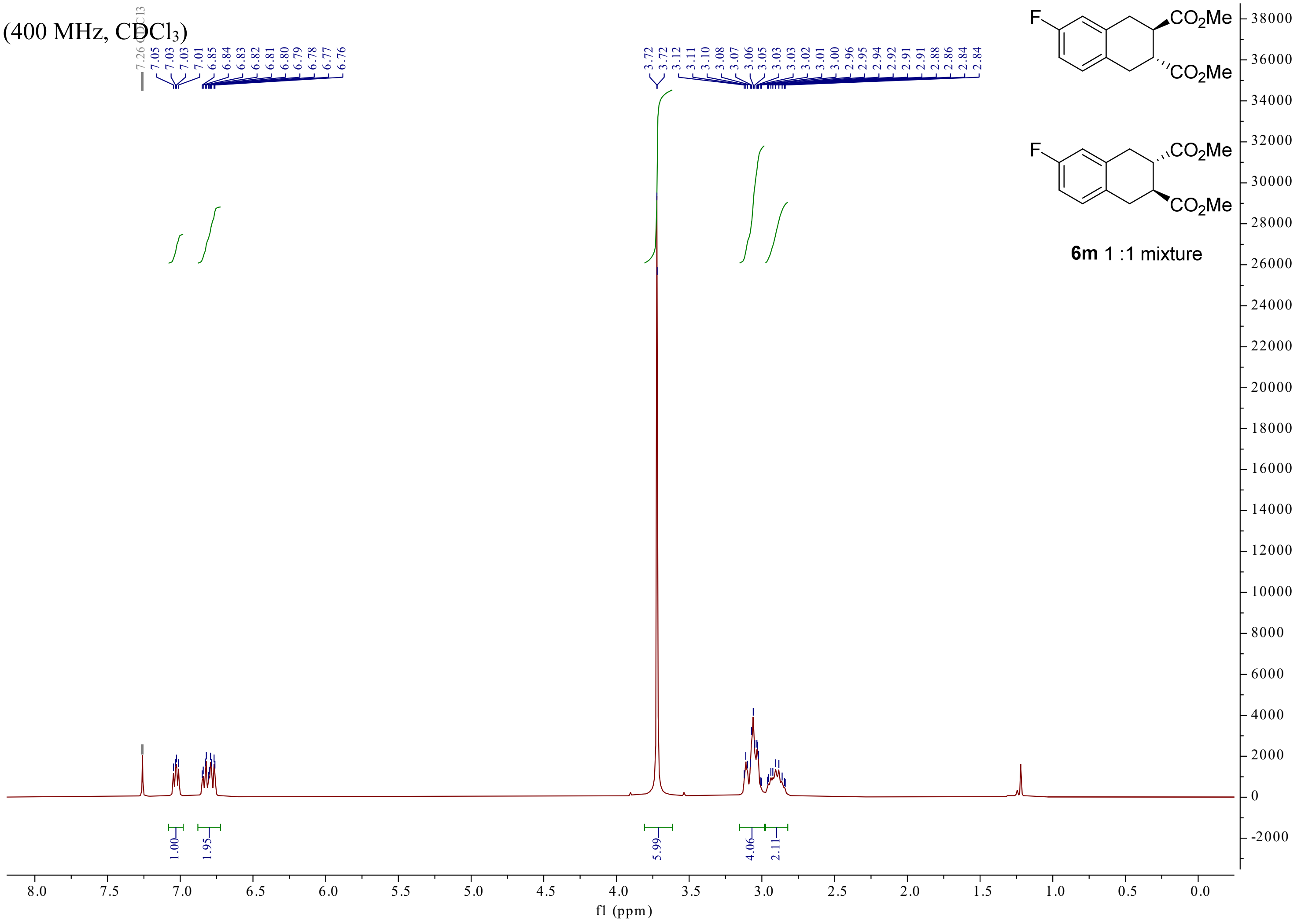

^{13}C NMR (101 MHz, $CDCl_3$)

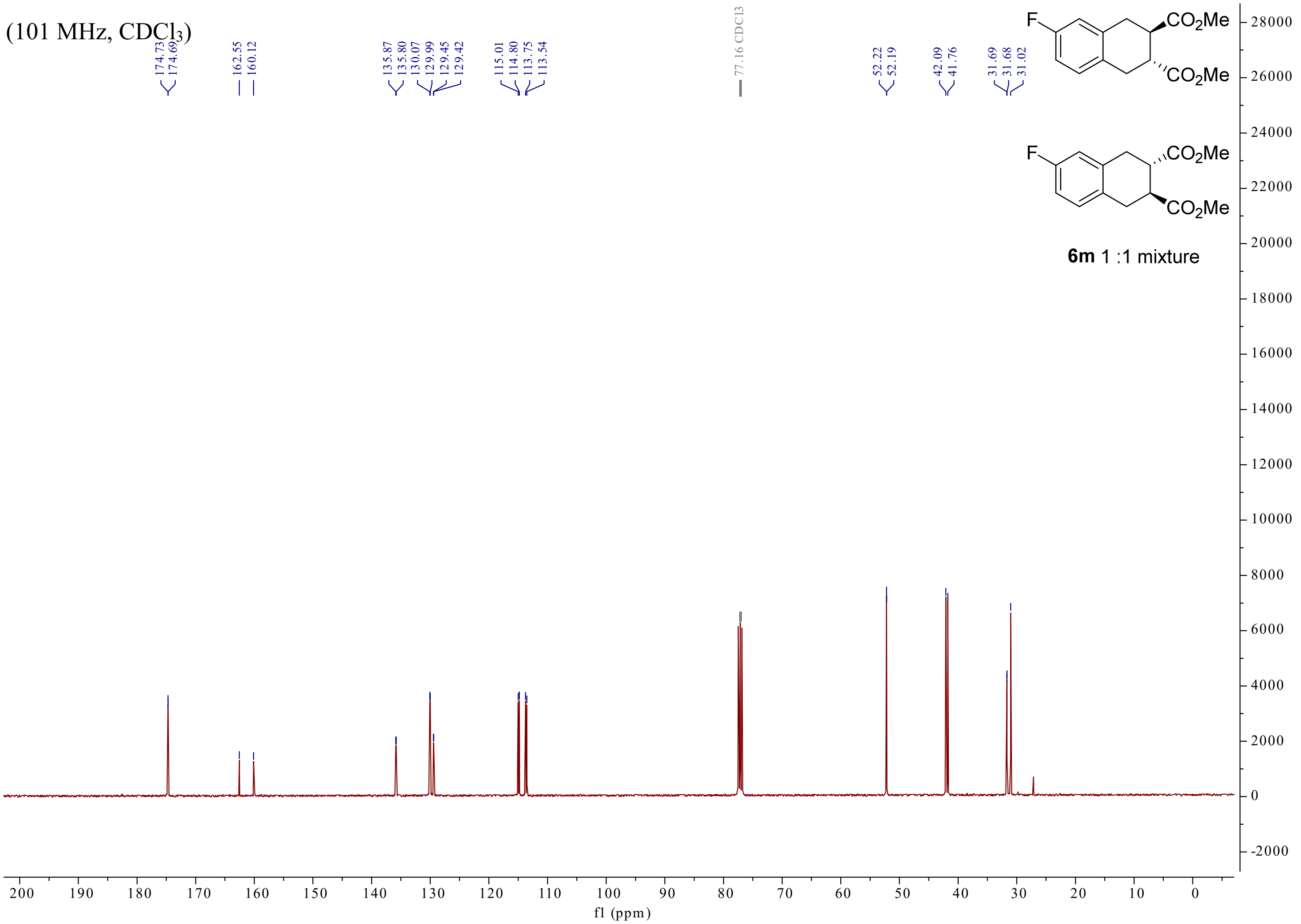

^{19}F NMR (376 MHz, $CDCl_3$)

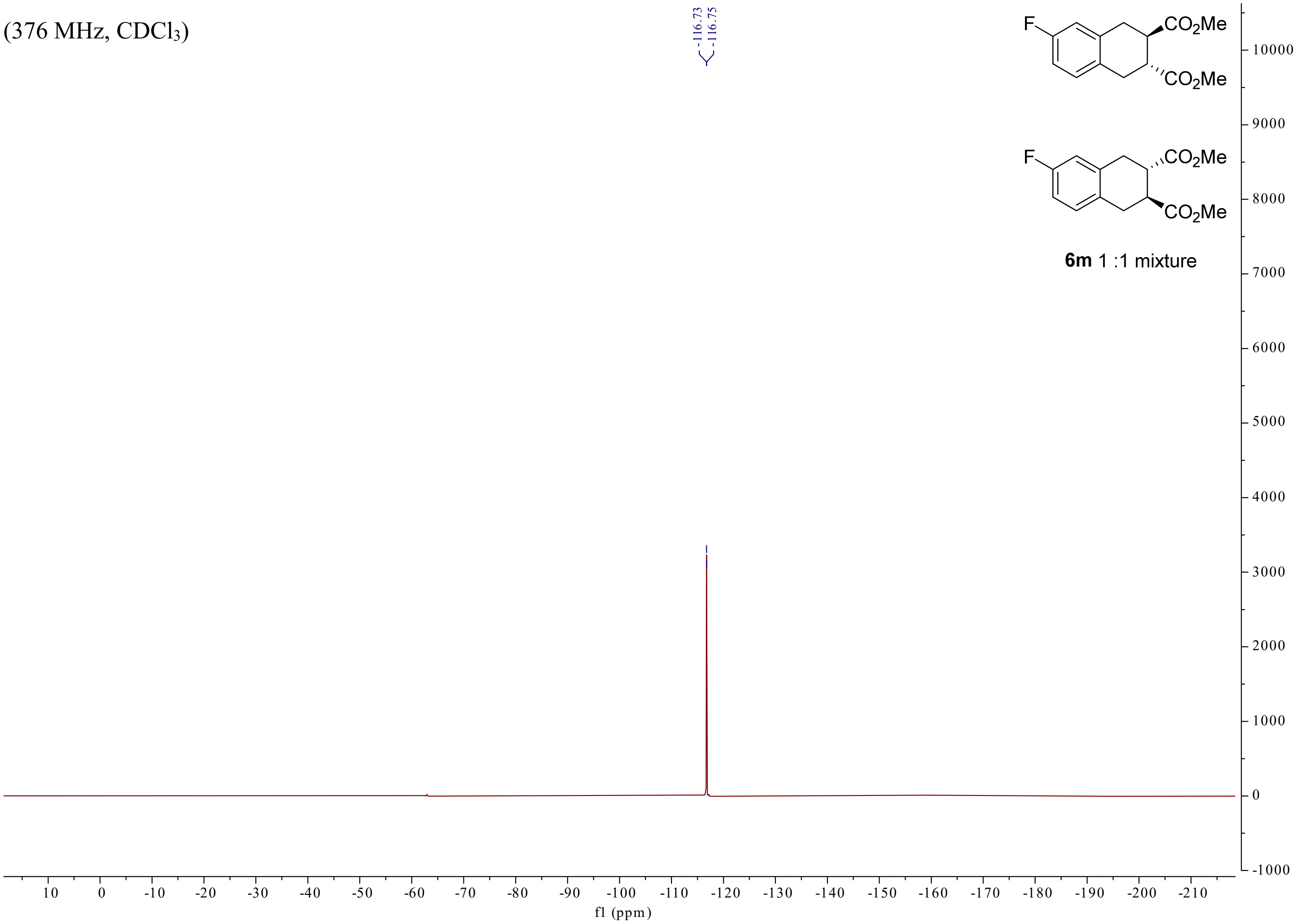

^{1}H NMR (400 MHz, $CDCl_3$)

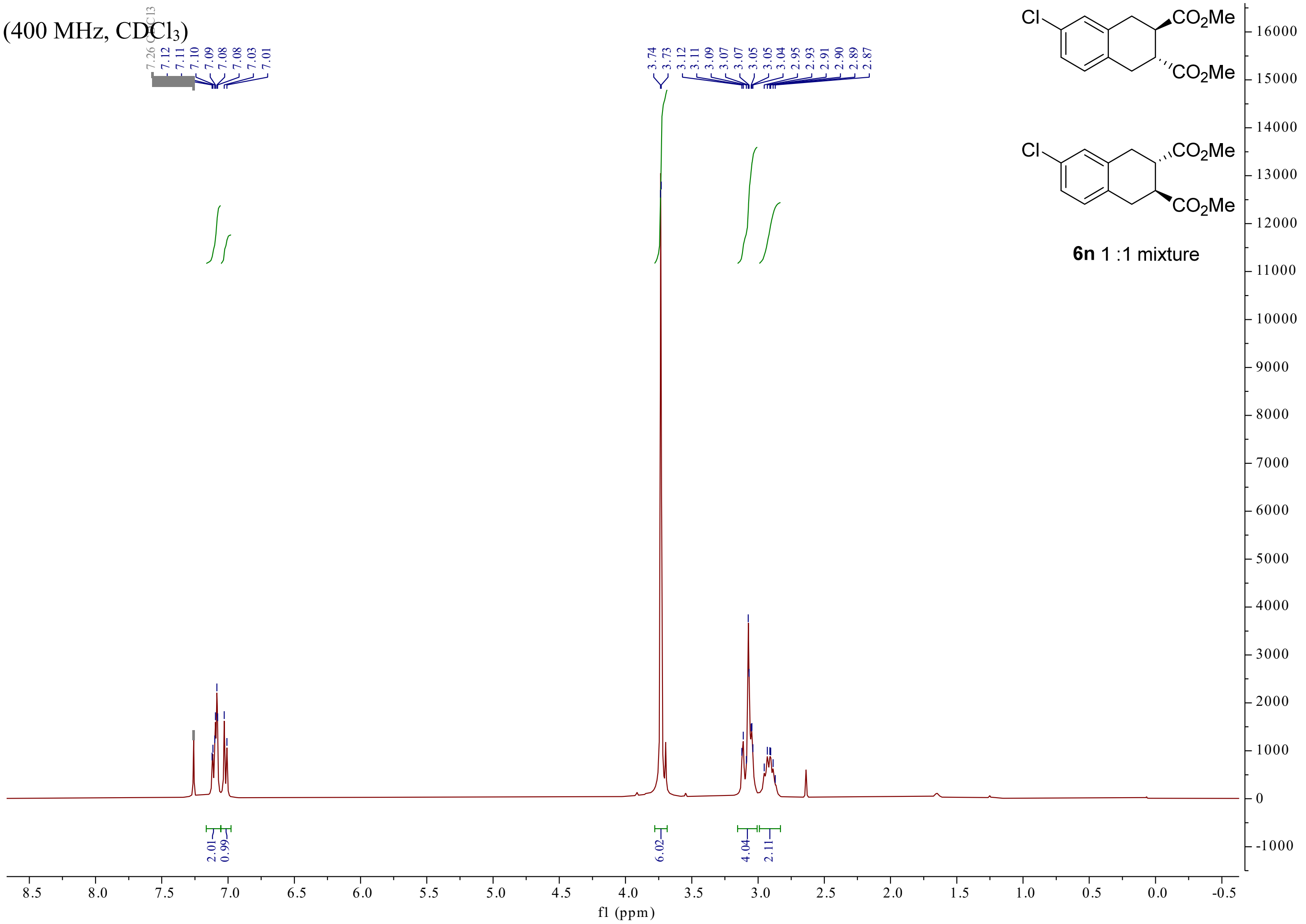

6n 1 :1 mixture

^{13}C NMR (101 MHz, $CDCl_3$)

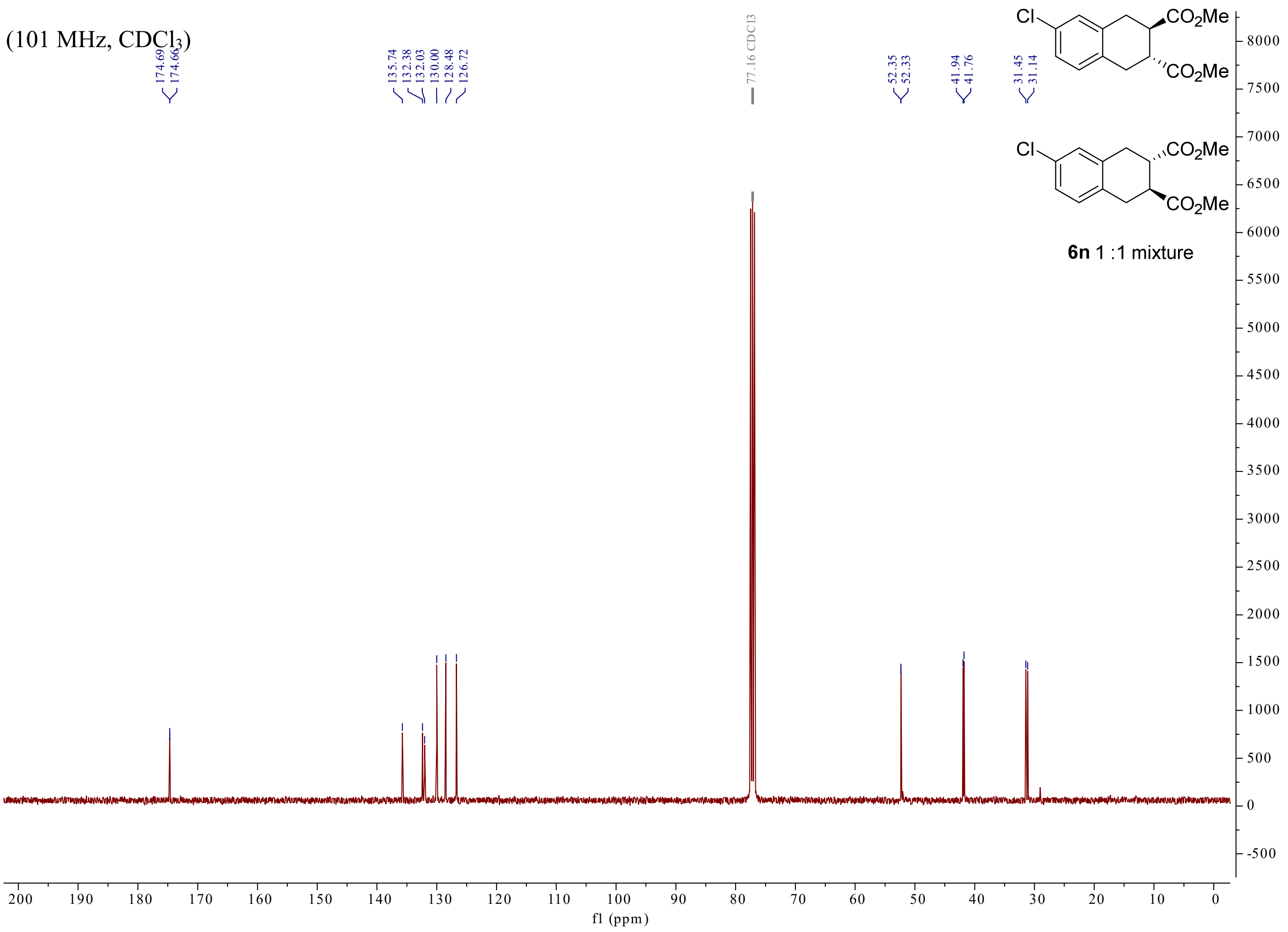

^1H NMR (400 MHz, $CDCl_3$)

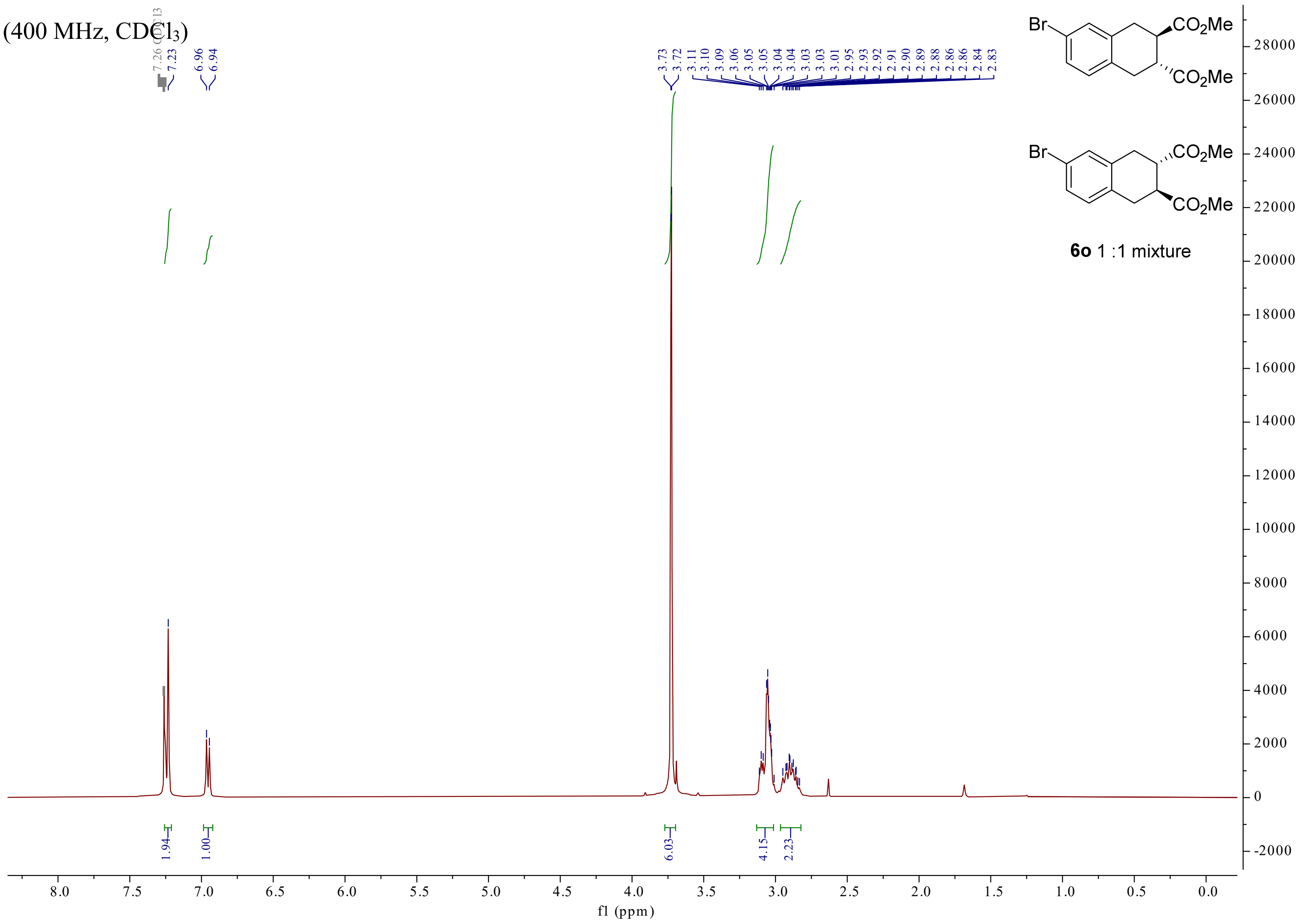

^{13}C NMR (101 MHz, $CDCl_3$)

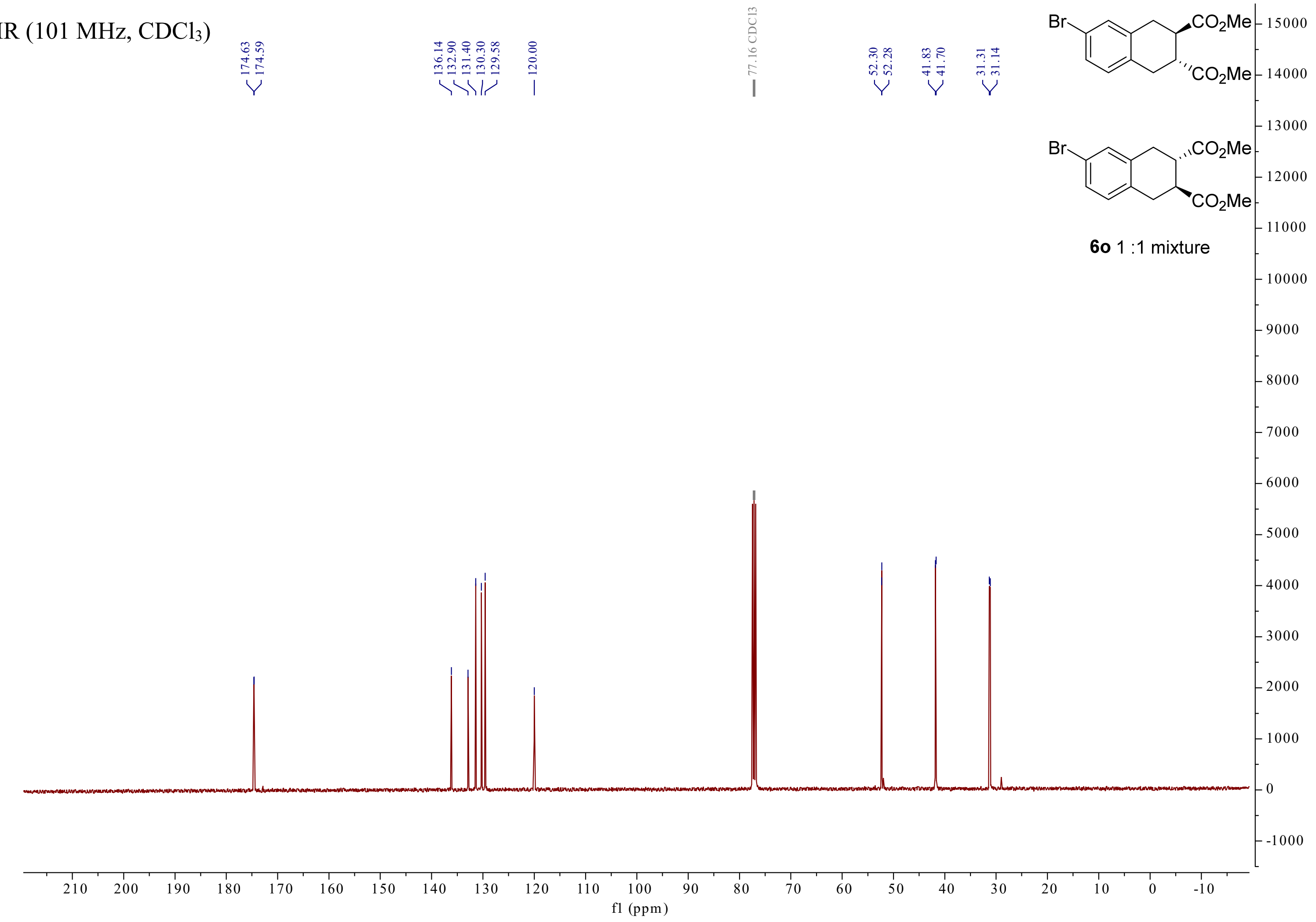

^{1}H NMR (400 MHz, $CDCl_3$)

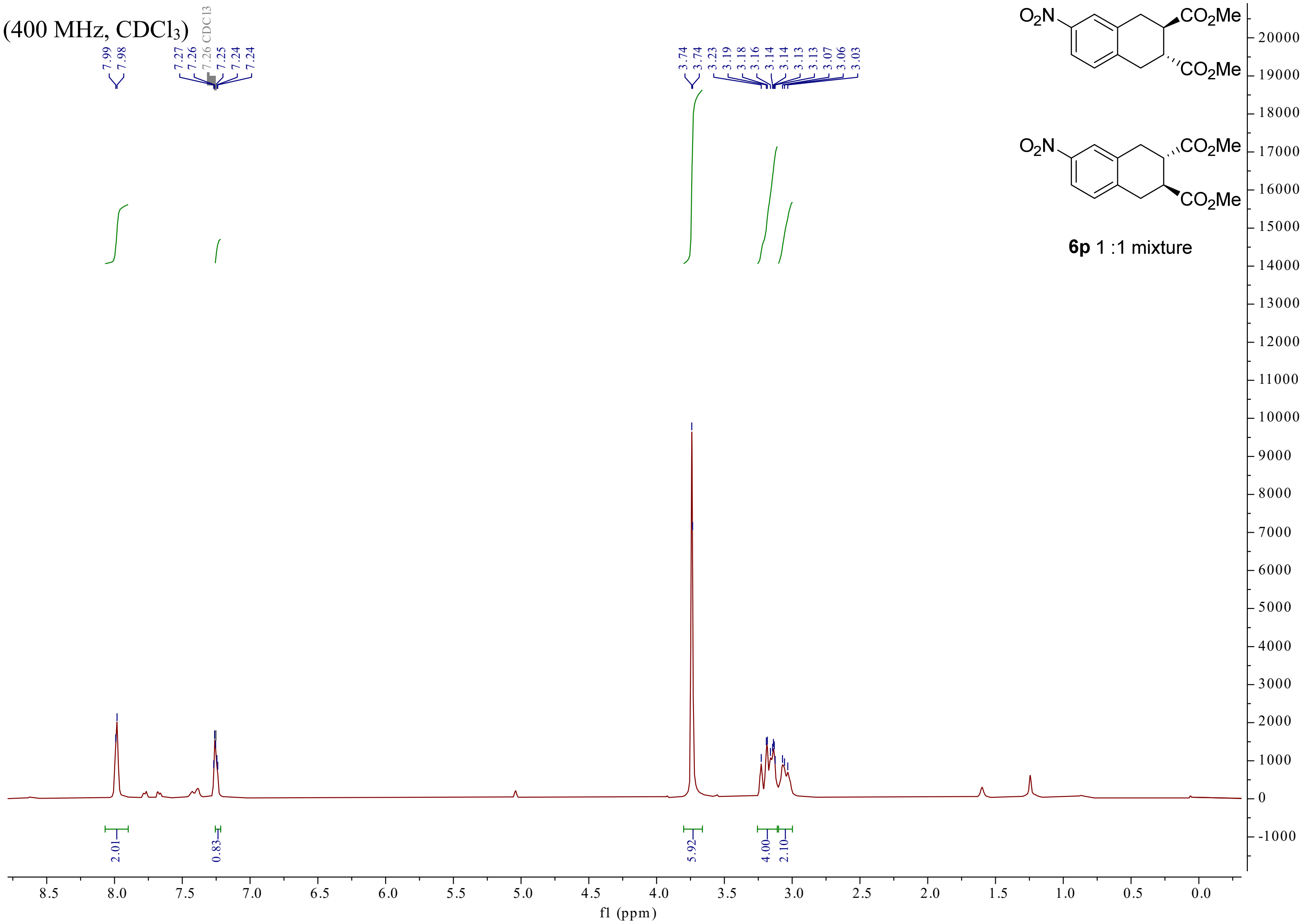

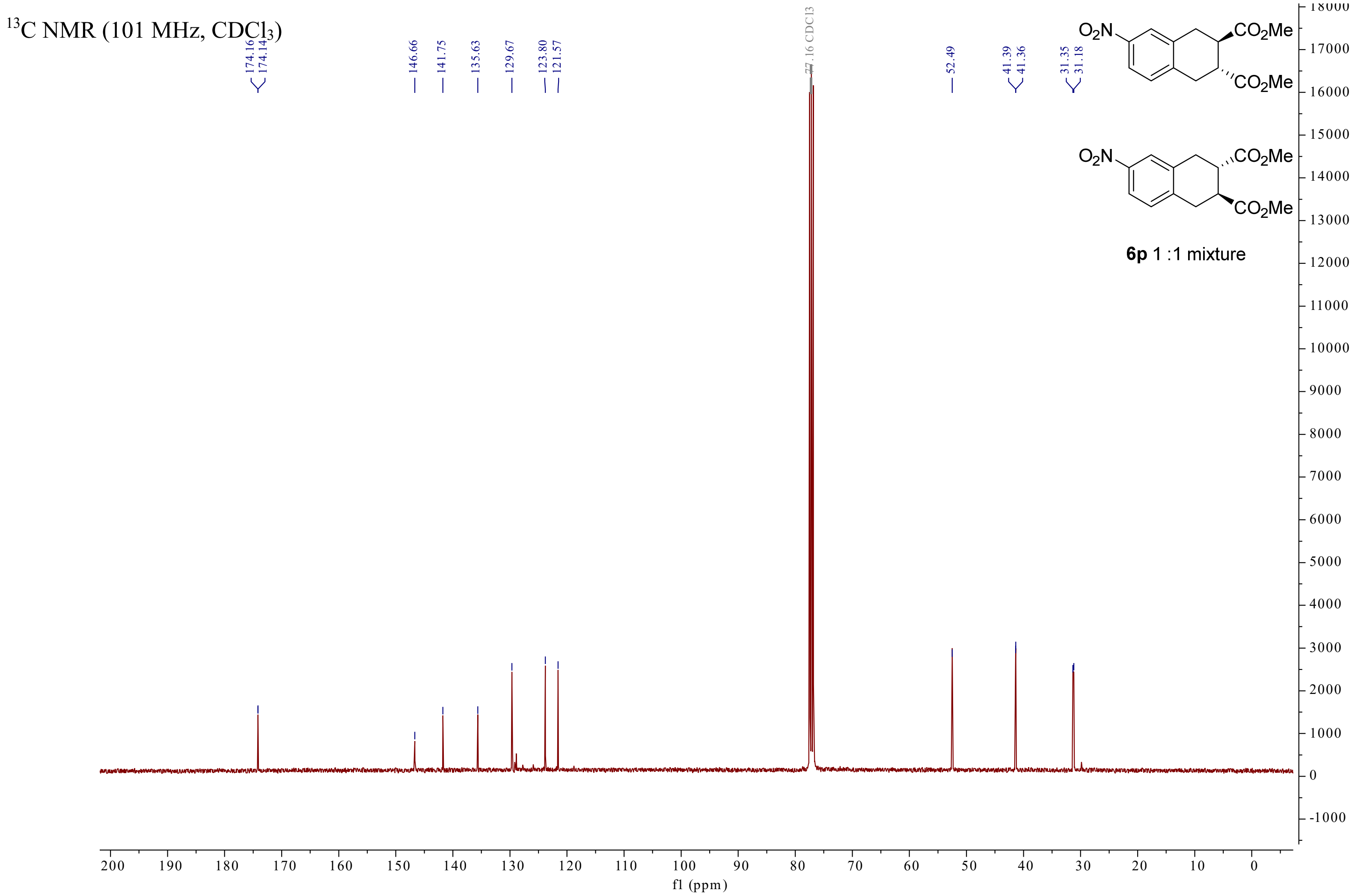
^{13}C NMR (101 MHz, CDCl$_3$)
174.16
174.14
146.66
141.75
135.63
129.67
123.80
121.57
77.16 CDCl3
52.49
41.39
41.36
31.35
31.18
O2N
CO2Me
CO2Me
O2N
CO2Me
CO2Me
6p 1 :1 mixture
fl (ppm)

^{1}H NMR (400 MHz, $CDCl_3$)

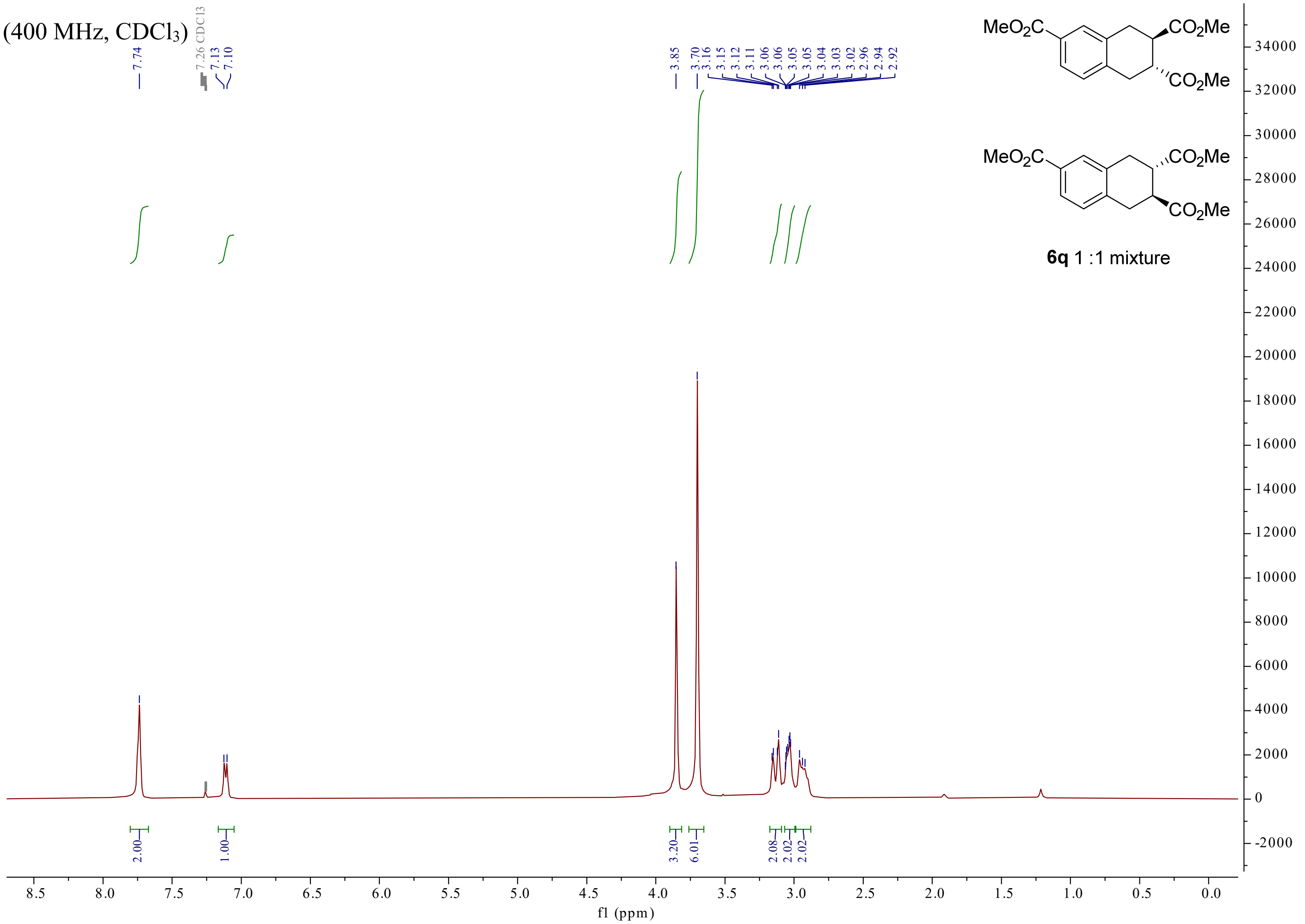

^{13}C NMR (101 MHz, $CDCl_3$)

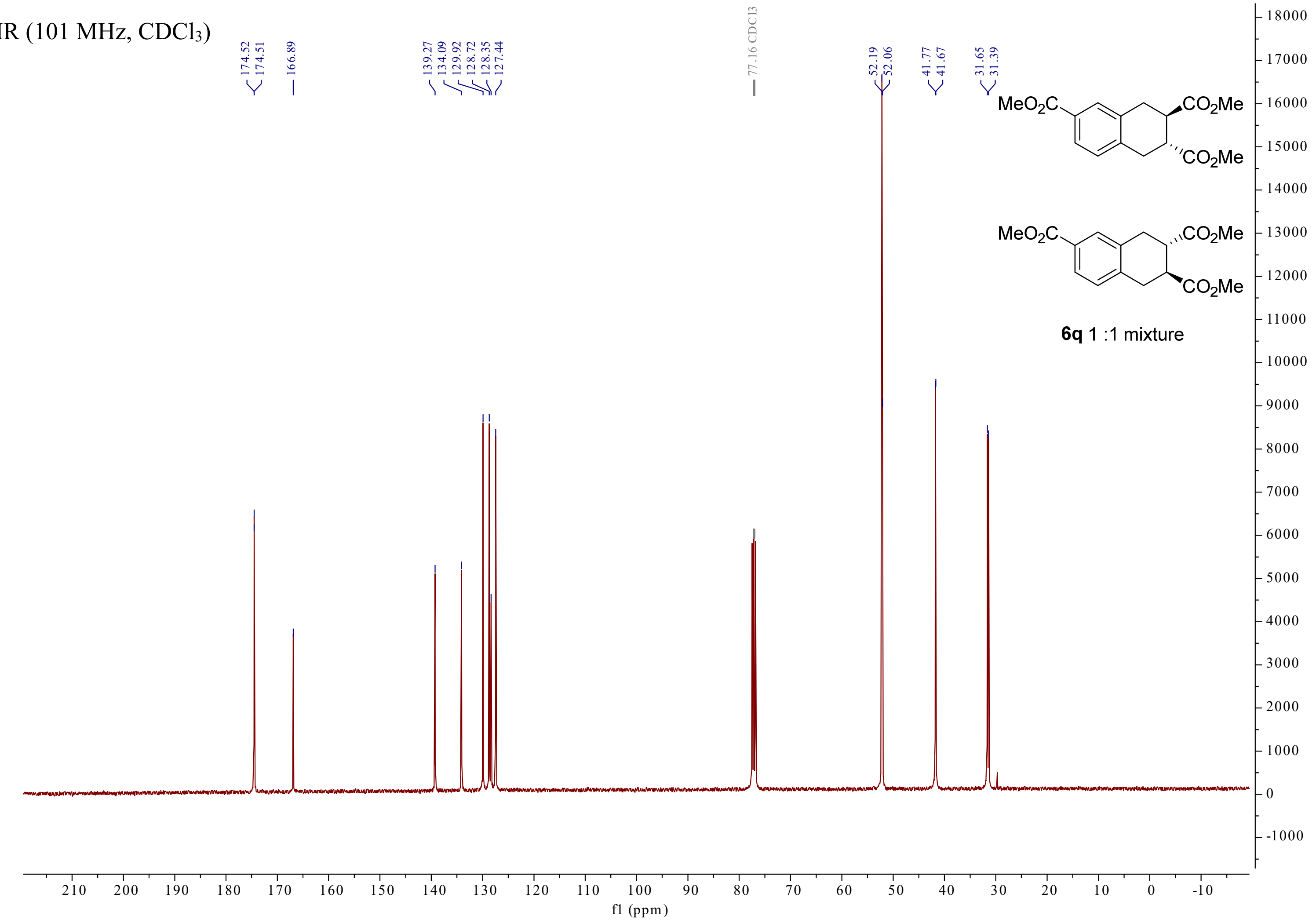

^{1}H NMR (400 MHz, $CDCl_3$)

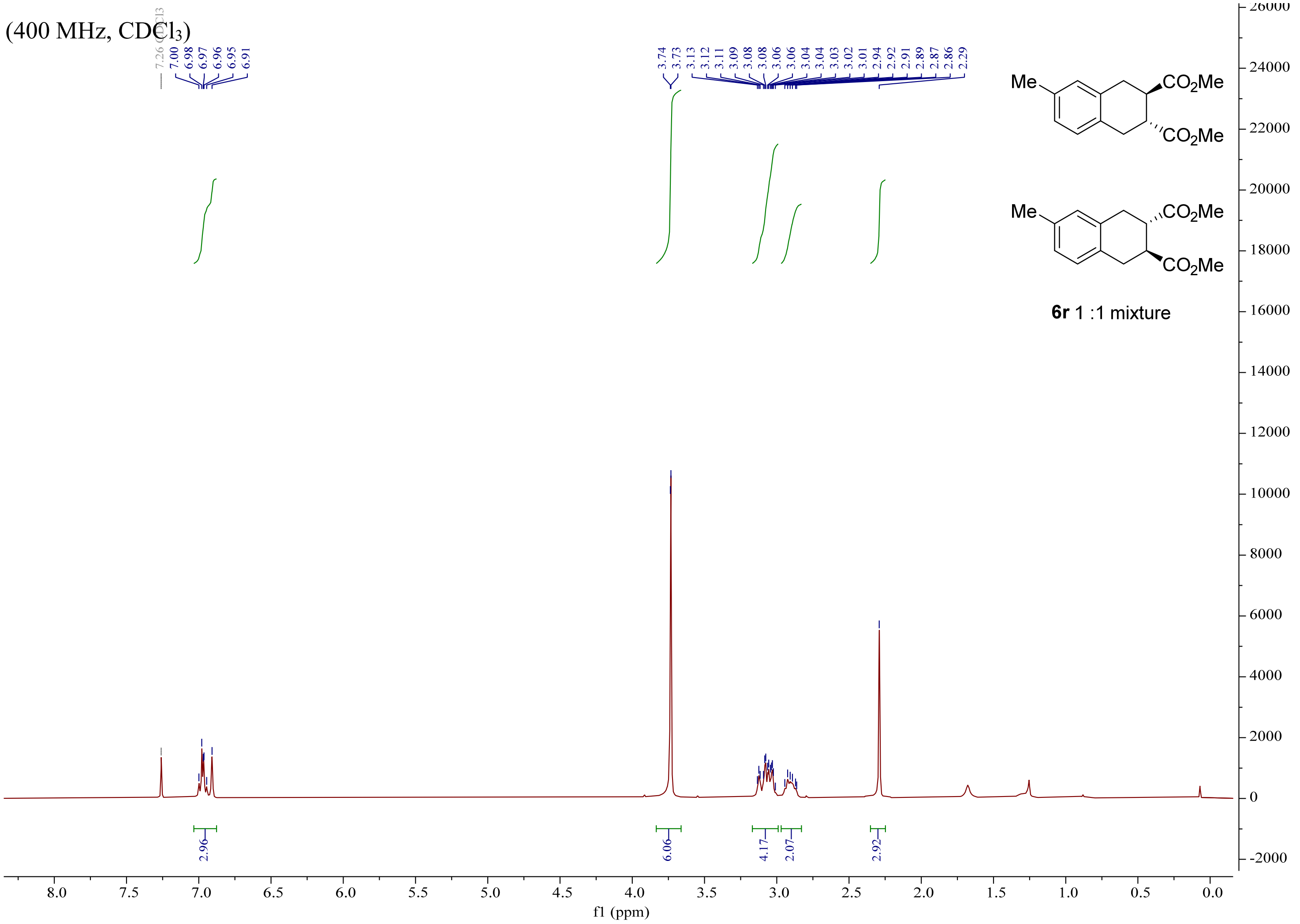

^{13}C NMR (101 MHz, $CDCl_3$)

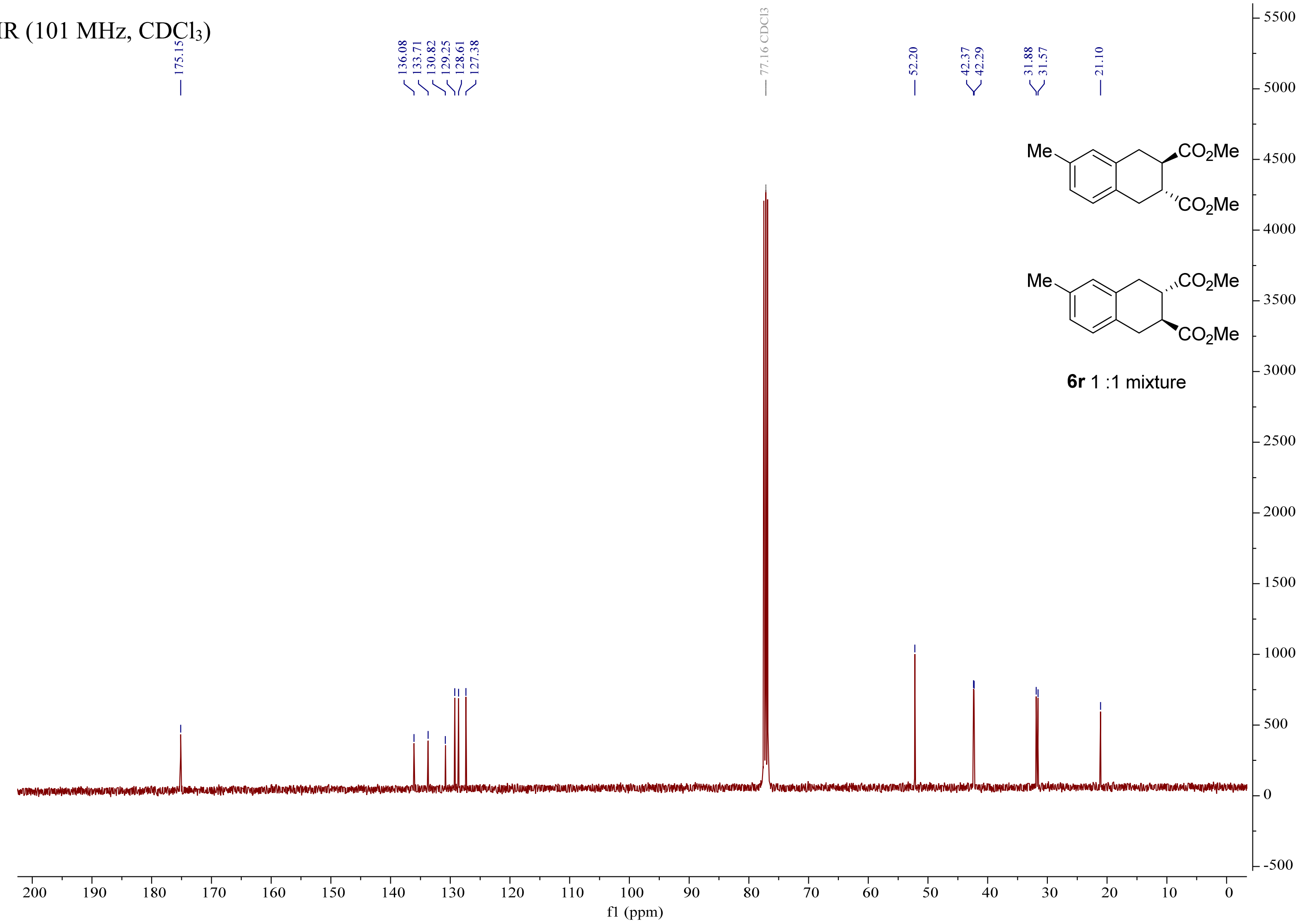

10. Single Crystal X-ray Diffraction of 6e

The colorless and transparent single crystals of **6e** was prepared by layering a deuterated chloroform with n-hexane at ambient temperature. A suitable crystal was selected and analyzed on a Bruker APEX-II CCD diffractometer. The crystal was kept at T = 100.00 K during data collection. Using Olex2, the structure was solved with the SHELXT structure solution program using Intrinsic Phasing and refined with the XL refinement package using Least Squares minimization.

Table S1. Crystal data and structure refinement for **6e**.

Data	**6e**
Empirical formula	$C_{13}H_{13}NO_2$
Formula Weight	215.24
Temperature / K	100.00
Crystal System	Monoclinic
Space group	$P2_1$
a/Å	6.188(3)
b/Å	7.968(4)
c/Å	21.356(10)
α/°	90
β/°	91.108(17)
γ/°	90
Volume/Å^3, Z	1052.8(9), 4
ρ_{calc}g/cm^3	1.358
Absorption coefficient / mm^{-1}	0.092
F(000)	456.0
Crystal size / mm^3	0.15 × 0.14 × 0.12
Radiation	MoKα (λ = 0.71073)
2θ range for data collection / °	5.458 to 51.36
Index ranges	$-7 \leq h \leq 7$, $-9 \leq k \leq 9$, $-26 \leq l \leq 25$
Independent reflections	3834 [R_{int} = 0.1019, R_{sigma} = 0.0929]
Reflections collected	8910
Max. and min. transmission	0.658 and 0.469
Data / restraints / parameters	3834/19/291
Goodness-of-fit on F^2	1.101
Final R indexes [I>=2σ (I)]	R_1 = 0.1060, wR_2 = 0.2730
Final R indexes [all data]	R_1 = 0.1167, wR_2 = 0.2783
Largest diff. peak, hole / eÅ^{-3}	0.66/-0.41
Flack parameter	-0.7(10)

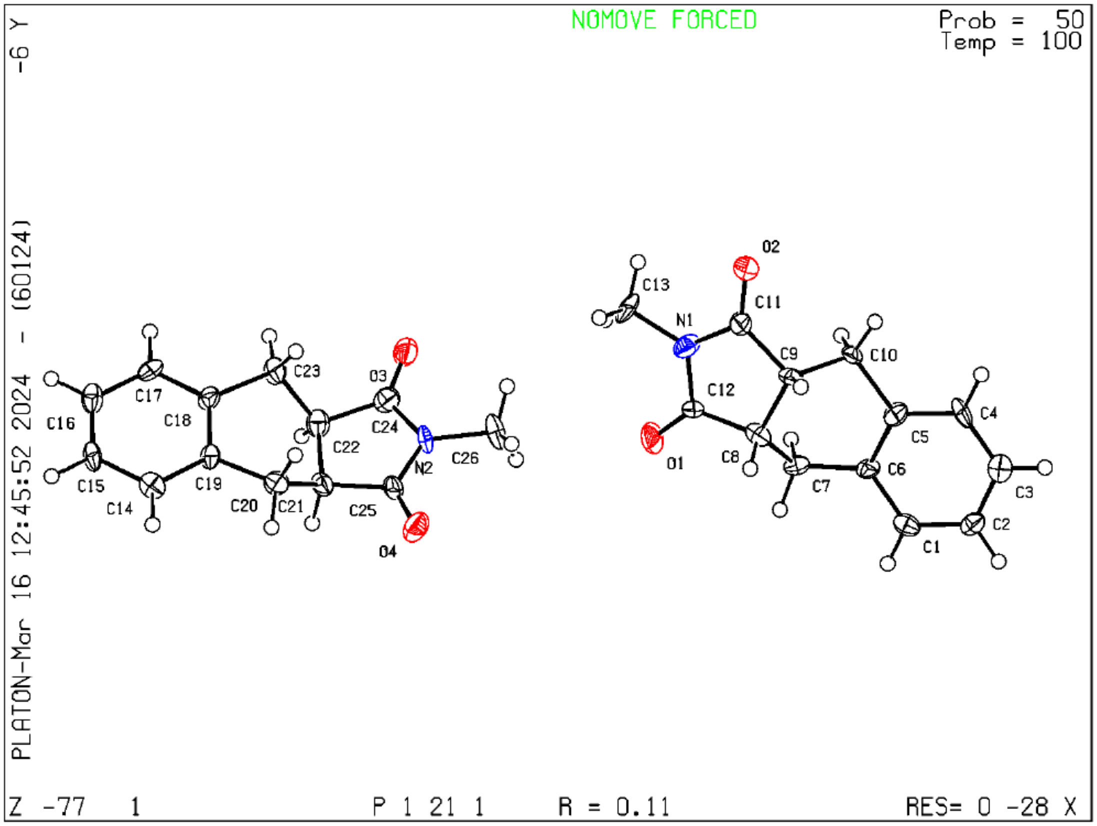

Figure S3. X-ray crystal structure of compound 6e with 50% ellipsoid probability.
